# Virome of *Pseudostellaria heterophylla*: Identification and characterization of three novel carlaviruses and one novel amalgavirus associated with viral diseases of *Pseudostellaria heterophylla*

**DOI:** 10.3389/fmicb.2022.955089

**Published:** 2022-09-29

**Authors:** Yong Li, Sai Liu, Kun Guo, Wanlong Ding, Rong Wang

**Affiliations:** Institute of Medicinal Plant Development, Chinese Academy of Medical Sciences and Peking Union Medical College, Beijing, China

**Keywords:** *Pseudostellaria heterophylla*, virome, next-generation sequencing, novel viruses, *Carlavirus*, *Amalgavirus*

## Abstract

*Pseudostellaria heterophylla* is a traditional Chinese herbal medicine, which has been cultivated for hundreds of years. Viral diseases of *P. heterophylla* occur widely and limit the yield and quality of this medicinal plant. In this study, five leaf samples of *P. heterophylla* with typical viral symptoms were collected from four main producing regions that are distributed in Fujian, Guizhou, and Anhui Provinces in China and analyzed by next-generation sequencing. Comprehensive bioinformatics analyses revealed that nine viruses in five genera *Carlavirus*, *Potyvirus*, *Fabavirus*, *Cucumovirus*, and *Amalgavirus* infected *P. heterophylla*. Among these viruses, three novel and two known carlaviruses, tentatively designated *Pseudostellaria heterophylla* carlavirus 1, 2, and 3 (PhCV1, PhCV2, and PhCV3), Jasmine virus C isolate Ph (Ph-JVC) and Stevia carlavirus 1 isolate Ph (Ph-StCV1), respectively, were first identified in *P. heterophylla*. PhCV1-3 share a similar genomic organization and clear sequence homology with members in the genus *Carlavirus* and could potentially be classified as new species of this genus. One novel amalgavirus, tentatively designated *P. heterophylla* amalgavirus 1 (PhAV1), was first identified in *P. heterophylla.* It had a typical genomic organization of the genus *Amalgavirus*. In PhAV1, the + 1 programmed ribosomal frameshifting, which is prevalent in most amalgaviruses, was identified and used in the expression of RNA-dependent RNA polymerase (RdRp). Combined with a phylogenetic analysis, PhAV1 could potentially be classified as new species of the genus *Amalgavirus*. In addition, multiple *Broad bean wilt virus 2* (BBWV2) variants, *Turnip mosaic virus* (TuMV), and *Cucumber mosaic virus* (CMV), which have been reported in *P. heterophylla*, were also detected in this study. The distribution of PhCV1-3, Ph-JVC, Ph-StCV1, TuMV, BBWV2, and CMV in four production regions in Fujian, Guizhou, and Anhui Provinces was determined. This study increased our understanding of *P. heterophylla* virome and provided valuable information for the development of a molecular diagnostic technique and control of viral diseases in *P. heterophylla*.

## Introduction

*Pseudostellaria heterophylla* (Miq.) Pax is also known as “Tai-zi-shen” or “Hai-er-shen” in China. It is a member of the family Caryophyllaceae and one of the most popular traditional Chinese herbal medicines. It can be used to treat spleen deficiency, anorexia, weakness after illness, and spontaneous perspiration symptoms (Pang et al., 2011; Sheng et al., 2011; Guo et al., 2014; Choi et al., 2017). *P. heterophylla* is widely distributed in northeastern Asia, including China, Japan, and Korea. In China, *P. heterophylla* has been cultivated for hundreds of years, and its commercial areas of production are primarily distributed in Guizhou, Fujian, Anhui, Shandong, and Jiangsu Provinces. Most of the *P. heterophylla* used in the medicinal commodity market is grown in Shibing County (in Guizhou Province), Zherong County (in Fujian Province), and Xuancheng City (in Anhui Province; Kang et al., 2016).

*Pseudostellaria heterophylla* primarily propagates asexually through root tubers, which facilitates the transmission of viral diseases across generations. The infections then spread to different regions through the transportation of seedlings. Owing to the long-term vegetative propagation of these plants, the incidence of viral diseases increases yearly, which can result in rates of infection as high as 90% or even 100% (Huang and Lin, 2004; Wang et al., 2022). Such severe infections reduce the yield and quality of *P. heterophylla*. *P. heterophylla* infected by virus(es) display symptoms, such as foliar mottles, mosaics, ringspots, and leaf malformations, as well as the dwarfing of plants. To date, TuMV (of the genus *Potyvirus,* family *Potyviridae*), BBWV2 (of the genus *Fabavirus*, family *Secoviridae*), CMV (of the genus *Cucumovirus*, family *Bromoviridae*), and *Tobacco mosaic virus* (TMV, of the genus *Tobamovirus*, family *Virgaviridae*) are the four viruses that have been reported to infect *P. heterophylla* (Song and Pu, 1991).

The genus *Carlavirus* of the family *Betaflexiviridae* comprises 61 confirmed species (ICTV).^1^ Carlaviruses have a monopartite, single-stranded, and positive-sense RNA genome that ranges from 8.3 to 8.7 kb long. Their genomic RNAs encode six open reading frames (ORFs). ORF1 encodes a viral replicase (Rep) and three overlapping ORFs, including ORF2–ORF4, encode the triple gene block (TGB) proteins 1, 2, and 3 that facilitate the movement of viruses. ORF5 encodes the coat protein (CP), and ORF6 encodes a cysteine-rich protein (CRP; Adams et al., 2012a). CRP serves as an RNA silencing suppressor and a determinant of symptoms and/or pathogenicity (Deng et al., 2015; Fujita et al., 2018). Most carlaviruses are transmitted naturally by aphids in a non-persistent manner, while some of them are transmitted by whiteflies (*Bemisia tabaci*; Adams et al., 2012a). All the carlaviruses are mechanically transmissible (Adams et al., 2012a).

*Amalgaviridae* is a recently reported family of double-stranded RNA viruses that is an amalgam of the families *Partitiviridae* and *Totiviridae*, which encode proteins phylogenetically related to former, but their structure is more closely related to the latter (Martin et al., 2011; Krupovic et al., 2015). It contains two genera, *Amalgavirus* and *Zybavirus*. *Amalgavirus* includes nine viral species that infect plants (ICTV).^1^ Putative amalgaviruses have also been reported in fungi and bryophytes (Koloniuk et al., 2015; Vendrell-Mir et al., 2021). These amalgaviruses are monopartite and have small dsRNA genomes that are 3.3–3.5 kb long and encode two partially overlapping ORFs. ORF1 encodes a putative CP with unclear function, which is similar to the nucleocapsid proteins of the genera *Phlebovirus* and *Tenuivirus* (Krupovic et al., 2015) or the replication factory matrix-like protein (Isogai et al., 2011). In most amalgaviruses, there is a + 1 programmed ribosomal frameshifting (PRF) in ORF1. When it occurs, the codon frame changes from UUU_ CGN (ORF1) to U_UUC_GNN (ORF2; underlining indicates the codon boundary; N, any nucleotide), which causes the fusion of ORF1 and ORF2, and encodes an RdRp (Nibert et al., 2016; Park et al., 2018). Members of this family are transmitted vertically by seeds and are not thought to be capable of efficient extracellular transmission unless mediated by an unknown vector (Martin et al., 2006; Sabanadzovic et al., 2009).

To identify the viral species that infect *P. heterophylla* and provide a reference for the diagnosis, prevention, and control of viral diseases of *P. heterophylla*, the virome of *P. heterophylla* was analyzed by next-generation sequencing (NGS), a rapidly developing technique for viral detection and diagnosis that is suitable for a variety of plants, animals, and fungi (Mu et al., 2018; Jo et al., 2020; Kondo et al., 2020; Zhang et al., 2020). The five libraries used for NGS were prepared with *P. heterophylla* leaf samples collected from four different geographic regions in Guizhou, Fujian, and Anhui Provinces in China. Three novel and two known carlaviruses, one novel amalgavirus, multiple BBWV2 variants, TuMV, and CMV that infect *P. heterophylla* were identified.

## Materials and methods

### Plant samples

Leaf samples were collected from symptomatic *P. heterophylla* and transported to the laboratory under conditions of low temperature and humidity. The samples were stored at -80°C and used for paired-end RNA-Seq.

### RNA extraction, NGS, and data processing

Total RNA was extracted from the leaf tissue using a mirVana^™^ microRNA (miRNA) Isolation kit (Ambion, Thermo Fisher Scientific, Waltham, MA, United States) and treated with an RNA Clean XP Kit (Beckman Coulter, Brea, CA, United States) and an RNase-Free DNase Set (QIAGEN GmbH, Hilden, Germany) according to the manufacturer’s instructions. The quality and quantity of total RNA were measured using a NanoDrop spectrophotometer (Thermo Fisher Scientific) and Agilent2100 (Agilent Technologies, Santa Clara, CA, United States). After the removal of ribosomal RNA using a Ribo-Zero Magnetic Kit (Epicentre, Lucigen, Middleton, WI, United States), the libraries were built using a TruSeq RNA Sample Prep Kit (Illumina, San Diego, CA, United States). Barcoded libraries were paired-end sequenced on an Illumina HiSeq X platform according to the manufacturer’s instructions.^2^ Sequences of adaptors and low-quality traits were trimmed using the FASTX-Toolkit software,^3^ and clean reads were assembled *de novo* using CLC Genomics Workbench 6.0.4 (Qiagen, Valencia, CA, United States) according to the scaffolding contig algorithm. The second assembly was then conducted using CAP3 software.^4^ The final contigs were compared against the NCBI non-redundant (Nr) database using BLASTX with an *E*-value < 1*e*^−5^.

### Recovery of viral genomes

The genome of new *P. heterophylla* viruses were obtained by overlapping reverse transcription (RT)-PCR and rapid amplification of cDNA ends-PCR (RACE-PCR). The primers used in RT-PCR and RACE-PCR were designed based on the viral contig sequences using Primer Premier 6 (PREMIER Biosoft, Palo Alto, CA, United States; Supplementary Table S1). A RACE-PCR assay was conducted using a SMARTer RACE 5′/3′ Kit (Clontech, Mountain View, CA, United States). The PCR amplicons were purified by a TIANgel Midi Purification Kit (Tiangen, Beijing, China) and cloned into the pMD18 or pUC19 Vector (TaKaRa, Dalian, China). The sequence of each amplicon was determined from both directions of more than five clones by the biotechnology company Tsingke (Beijing, China). The full-length genome of each virus was assembled from all amplicons of the virus using DNAMAN 6.0 (Lynnon Biosoft, Quebec City, Canada).

### Sequence analysis and read assembly

Viral genome organizations were analyzed using the ORF finder program.^5^ The conserved domains of ORFs were analyzed using the Conserved Domain Search Service on the NCBI website.^6^ The multiple sequence alignment, conserved domains analysis of the RdRps and CRPs of carlaviruses, and prediction of the PRF motif in RdRp of amalgaviruses were performed using CLC Genomics Workbench 21.0.5 (Qiagen). Pairwise comparisons between viruses were performed using MAFFT program^7^ and displayed by Sequence Demarcation Tool (SDT) software using a color-coded matrix (Muhire et al., 2014).

### Phylogenetic analysis

Each of the new viruses and its closely related viruses retrieved from the NCBI databases were aligned using the ClustalW program. Phylogenetic analyses were performed using the maximum-likelihood method (carlaviruses and amalgaviruses) or the neighbor-joining method (BBWV2) with 1,000 bootstrap replicates in MEGA X (Kumar et al., 2018).

## Results

### Sample collection and symptom description

**Figure 1:**
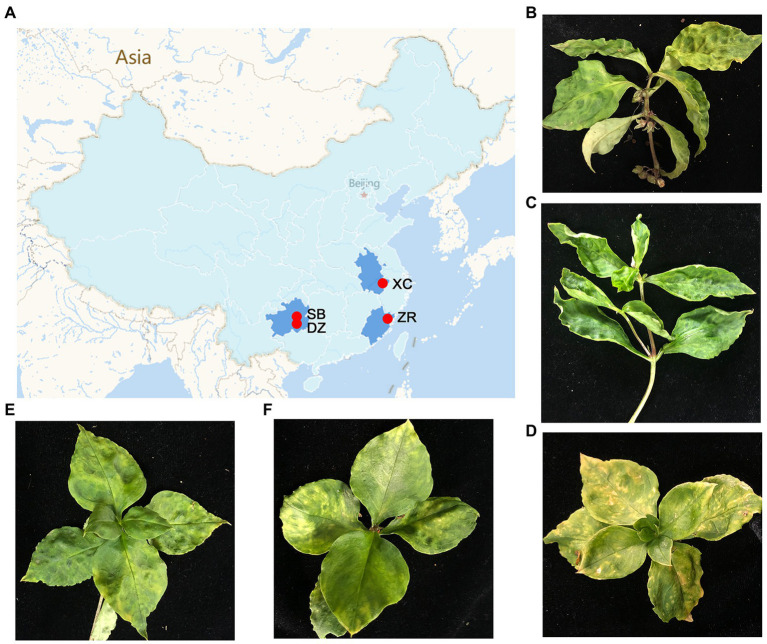
Geographical regions of the *Pseudostellaria heterophylla* samples collected in China and the leaf symptoms of *P. heterophylla* viral diseases. **(A)** A map that displays the four different geographical regions in China in which the *P. heterophylla* samples were collected. **(B–F)** Symptoms displayed on *P. heterophylla* leaves collected from SB, XC, ZR-HB, DZ, and ZR-GX, respectively. DZ and SB represent Danzhai County (DZ) and Shibing County (SB) in Guizhou Province, respectively. ZR-HB and ZR-GX represent Huangbai village (ZR-HB) and Gongxi village (ZR-GX) in Zherong County in Fujian Province, respectively. XC represents Xuancheng city (XC) in Anhui Province.

Five *P. heterophylla* leaf samples were collected from the four different main production regions of *P. heterophylla* in June of 2020 and 2021, including one sample in Shibing County (SB) and one sample in Danzhai (DZ) County in Guizhou Province, two samples in Zherong County (ZR-HB and ZR-GX) in Fujian Province and one sample in Xuancheng city (XC) in Anhui Province, in China (Figure 1A; Supplementary Table S2). The samples displayed typical viral symptoms, such as foliar mosaics, ringspots, mottles, and leaf malformations (Figures 1B–F). Each sample was used to generate a library for paired-end RNA-Seq. For simplicity, the libraries were named based on the geographical regions that the samples were collected from.

### Transcriptome assembly and virus identification

**Figure 2:**
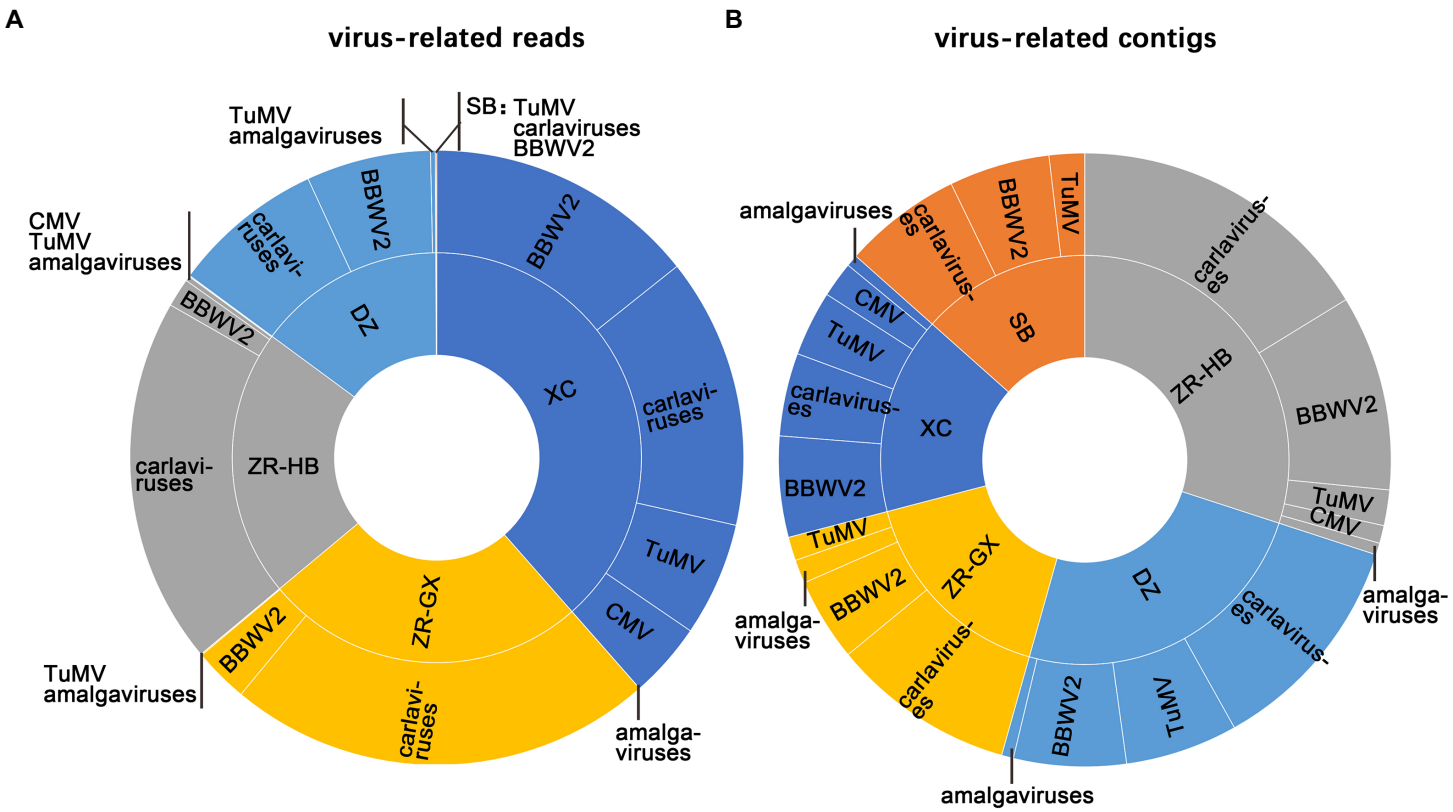
The identification of viruses in each sample. The proportion of identified viruses from all five libraries based on viral-associated reads **(A)**, and viral-associated contigs **(B)**. SB, DZ, ZR-GX, ZR-HB, and XC were library names designated based on geographical regions.

A total of 59,932,792–142,685,292 trimmed reads were individually generated from five independent libraries (Supplementary Table S3). Assembly of the clean reads generated 95,378 to 277,230 contigs that ranged from 200 to 10,252 nucleotides (nt) in size (Supplementary Table S3). A BLASTX analysis of the contigs revealed that 320 virus-related contigs representing five different viral taxa were obtained (Table 1). They included TuMV, BBWV2, carlaviruses, CMV, and the amalgaviruses. TuMV, BBWV2, and carlaviruses were identified in all five libraries. Approximately 94.06% (301 of 320) of the viral-related contigs, which were assembled from approximately 95.75% (150,587,101 of 157,276,057) viral-related reads, were homologous to the carlaviruses, TuMV or BBWV2 (Figures 2A,B; Table 1). Followed by CMV and the amalgaviruses, CMV was identified in the libraries ZR-HB and XC, and amalgaviruses were identified in the libraries ZR-HB, ZR-GX, DZ, and XC. Approximately 5.94% (19 of 320) of the viral-related contigs, which were assembled from approximately 4.25% (6,688,956 of 157,276,057) viral-related reads, were homologous to CMV or the amalgaviruses (Figure 2A,B; Table 1). TMV, a virus previously reported to infect *P. heterophylla* (Gao et al., 1993), was not identified in this study. Based on the viral-related reads, carlaviruses were the dominant viruses in the libraries DZ, ZR-HB, and ZR-GX; TuMV was the major virus in library SB, and BBWV2 was the major virus in library XC (Figure 2A; Table 1).

**Table 1:** Information of the viral reads and contigs for the viruses identified in *Pseudostellaria heterophylla* in China.

Library name^a^	Contigs-related viruses	Number of viral reads	Percentage of viral reads (%)	Number of viral contigs	Percentage of viral contigs (%)	Viral contig lengths
DZ	Carlaviruses	12,601,625	54.0389	38	48.72	257–8,133
	BBWV2	10,321,512	44.2612	19	24.36	212–5,313
	TuMV	395,647	1.6966	19	24.36	225–9,947
	Amalgaviruses	751	0.0032	2	2.56	1,143-2,018
SB	TuMV	121,299	95.9158	6	13.95	456–4,994
	Carlaviruses	4,417	3.4927	20	46.51	262–8,647
	BBWV2	748	0.5915	17	39.53	201–5,944
ZR-HB	Carlaviruses	30,387,069	91.3997	52	54.17	262–8,647
	BBWV2	2,367,422	7.1208	33	34.38	207–5,944
	CMV	411,973	1.2392	3	3.13	2,171–3,365
	TuMV	79,744	0.2399	6	6.25	456–4,994
	Amalgaviruses	155	0.0005	2	2.08	1,289-1,670
ZR-GX	Carlaviruses	35,399,063	88.4569	31	58.49	246–10,252
	BBWV2	4,524,414	11.3058	14	26.42	227–6,800
	TuMV	94,656	0.2365	4	7.55	300–9,857
	Amalgaviruses	282	0.0007	4	7.55	474–1,125
XC	BBWV2	22,491,422	37.1358	17	34.00	218–7,246
	Carlaviruses	22,380,694	36.9530	14	28.00	203–9,049
	TuMV	9,417,369	15.5491	11	22.00	218–9,845
	CMV	6,274,568	10.3600	6	12.00	259–3,140
	Amalgaviruses	1,227	0.0020	2	4.00	1,404-2,019
Total		157,276,057		320		

a Library names are named based on the sampling regions. DZ and SB represent Danzhai County (DZ) and Shibing County (SB) in Guizhou Province, respectively. ZR-HB and ZR-GX represent Huangbai village (ZR-HB) and Gongxi village (ZR-GX) in Zherong County in Fujian Province, respectively. XC represents Xuancheng city (XC) in Anhui Province.

### Turnip mosaic virus, CMV-II, and multiple BBWV2 variants were identified in *Pseudostellaria heterophylla*

The TuMV- and CMV-related contigs shared more than 96% nt sequence identity with TuMV and CMV in subgroup II (CMV-II). These results confirmed the presence of both viruses in *P. heterophylla*.

Six BBWV2 isolates were identified in *P. heterophylla*. One BBWV2 isolate was identified in libraries DZ and ZR-HB and designated BBWV2-DZ and -ZR-HB, respectively. Two BBWV2 isolates were identified in libraries XC and ZR-GX and designated BBWV2-XC1, -XC2, −ZR-GX1 and -ZR-GX2, respectively. Pairwise comparisons showed that the polyprotein encoded by RNA1 shared 73.41 to 98.15% nt sequence identities and 82.71 to 99.09% amino acid (aa) sequence identities with each other (Tables 2; Supplementary Table S4). The polyprotein encoded by RNA2 shared 70.12 to 98.18% nt sequence identities and 78.97 to 98.78% aa sequence identities with each other (Tables 2; Supplementary Table S4). Among them, RNA1 of ZR-GX1 and XC1 had lower nt identities (< 75.66%) compared with the other reported BBWV2 isolates. RNA2 of ZR-GX1 and XC2 had low nt similarities (< 74.19%) with the isolates reported (Table 2). The high proportion of nucleotide sequence changes did not result in significant changes in the aa sequences of the CP and Pro-Pol region, two indicators of the definition of new species (Sanfaçon et al., 2012). The Pro-Pol regions of ZR-GX1 and XC1 shared the highest aa sequence identities (89.81 and 87.04%, respectively) with that of the LNSY isolate (GenBank accession No. MN786954; Supplementary Table S4). The CP of ZR-GX1 and XC2 shared the highest aa sequence identities (85.31 and 85.81%, respectively) with that of the Yunnan (MW271032) and PC (MW939477) isolate, respectively (Supplementary Table S4). Since these sequence identity values are higher than the species demarcation threshold (80% aa sequence identity in Pro-Pol region or 75% aa sequence identity in CP) for the genus *Fabavirus* (Sanfaçon et al., 2012), these BBWV2 isolates cannot be defined as a new species in the genus *Fabavirus*. However, these results confirmed the variability of BBWV2 isolates in *P. heterophylla*.

**Table 2:** Pairwise nucleotide sequence identities of the BBWV2 isolates identified in *Pseudostellaria heterophylla.*

Isolates^a^	Nucleotide identity (%) Polyprotein/Pro-Pol region (RNA1); coat protein (RNA2)					Most similar isolate at the nucleotide level /GenBank accession number/nucleotide sequence identities (%)
	ZR-GX2	ZR-HB	DZ	XC1	XC2	
**BBWV2 RNA1**						
ZR-GX1	75.10/76.54	75.22/76.39	74.46/76.39	74.10/75.00	75.08/75.93	LN/MK116519/75.66
ZR-GX2		98.15/97.69	81.98/83.56	73.78/75.85	97.82/97.45	LN/MK116519/90.28
ZR-HB			82.23/83.84	73.59/75.64	97.81/97.45	LN/MK116519/90.46
DZ				73.41/75.46	82.27/83.56	LN/MK116519/82.70
XC1					73.70/75.93	LNSY/MN786954/74.58
XC2						LN/MK116519/90.36
**BBWV2 RNA 2**						
ZR-GX1	72.08/74.61	72.41/74.00	72.55/74.94	72.42/74.72	71.47/74.00	PAP1/KC634010/74.19
ZR-GX2		79.72/81.61	97.78/97.56	98.15/98.17	70.24/73.61	IP/AB018698/93.50
ZR-HB			79.84/81.61	79.66/81.50	70.12/73.56	PC/MW939477/89.95
DZ				98.18/98.17	70.24/73.44	IP/AB018698/93.38
XC1					70.49/73.78	IP/AB018698/93.50
XC2						PC/MW939477/73.44

a ZR-GX1, ZR-GX2, ZR-HB, DZ, XC1, and XC2 represent the BBWV2 isolates isolated from the samples collected from Gongxi village (ZR-GX) and Huangbai village (ZR-HB) in Zherong County in Fujian Province, Danzhai County (DZ) in Guizhou Province, and Xuancheng city (XC) in Anhui Province, respectively.

**Figure 3:**
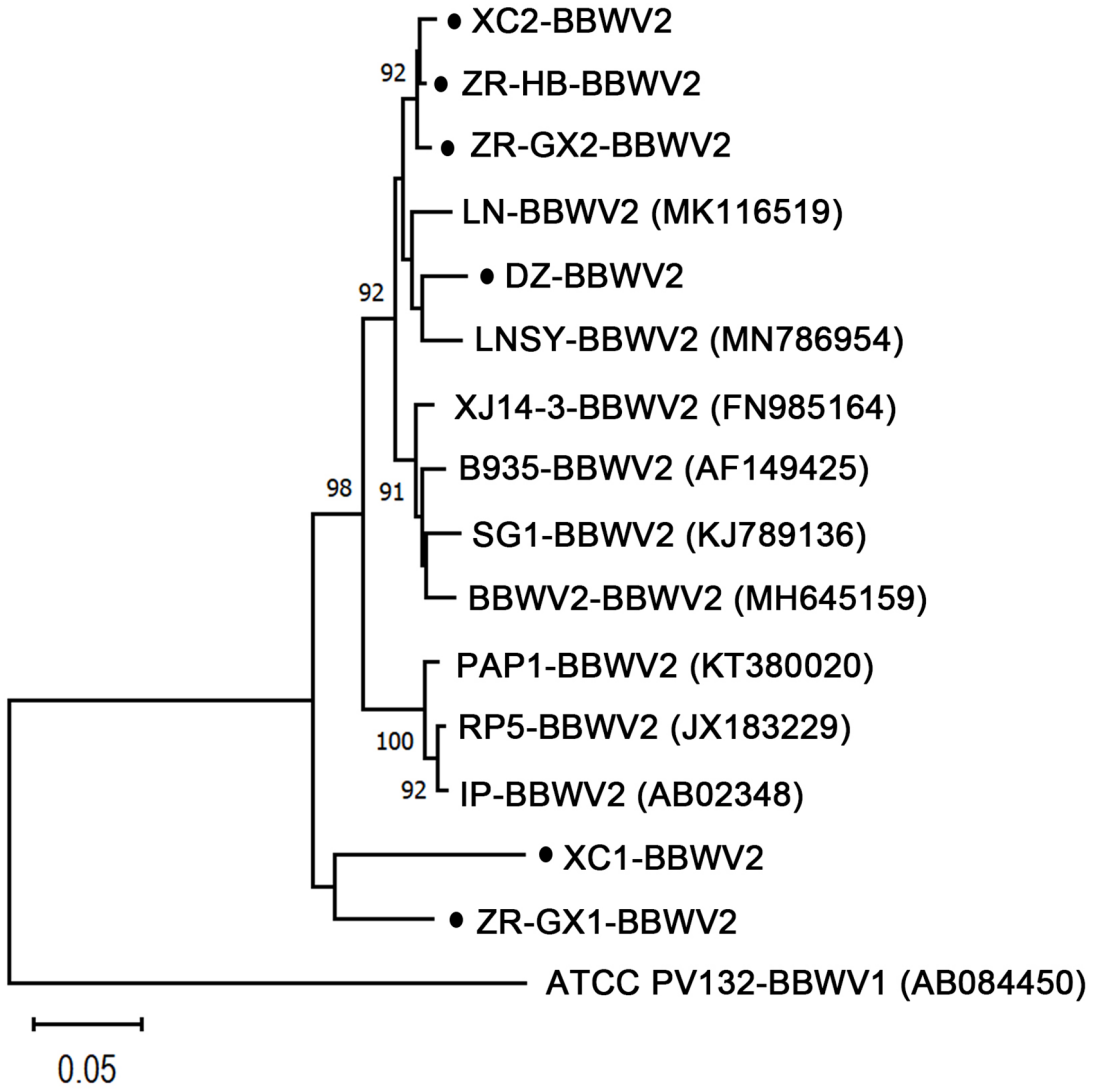
Phylogenetic relationships of BBWV2 isolates from *Pseudostellaria heterophylla* (ZR-GX1-, ZR-GX2-, ZR-HB-, DZ-, XC1-, and XC2-BBWV2) and the nine isolates that were the most similar to those of BBWV2 based on the amino acid sequences of the Pro-Pol region. The phylogenetic trees were constructed using the neighbor-joining method in MEGA X software with a bootstrap of 1,000 replicates. The black dots represent isolates identified in this study. ZR-GX1-, ZR-GX2-, ZR-HB-, DZ-, XC1- and XC2-BBWV2 represent the isolates isolated from the samples collected from Gongxi village (ZR-GX) and Huangbai village (ZR-HB) in Zherong County in Fujian Province, Danzhai County (DZ) in Guizhou Province, and Xuancheng city (XC) in Anhui Province, respectively.

A phylogenetic analysis based on the amino acid sequences of the Pro-Pol region of the polymerase showed that the BBWV2 isolates DZ, ZR-HB, ZR-GX2, and XC2 clustered closely with the isolates LN and LNSY, while the isolates ZR-GX1 and XC1 did not cluster (Figure 3).

### Three novel and two known carlaviruses were identified in *Pseudostellaria heterophylla*

**Figure 4:**
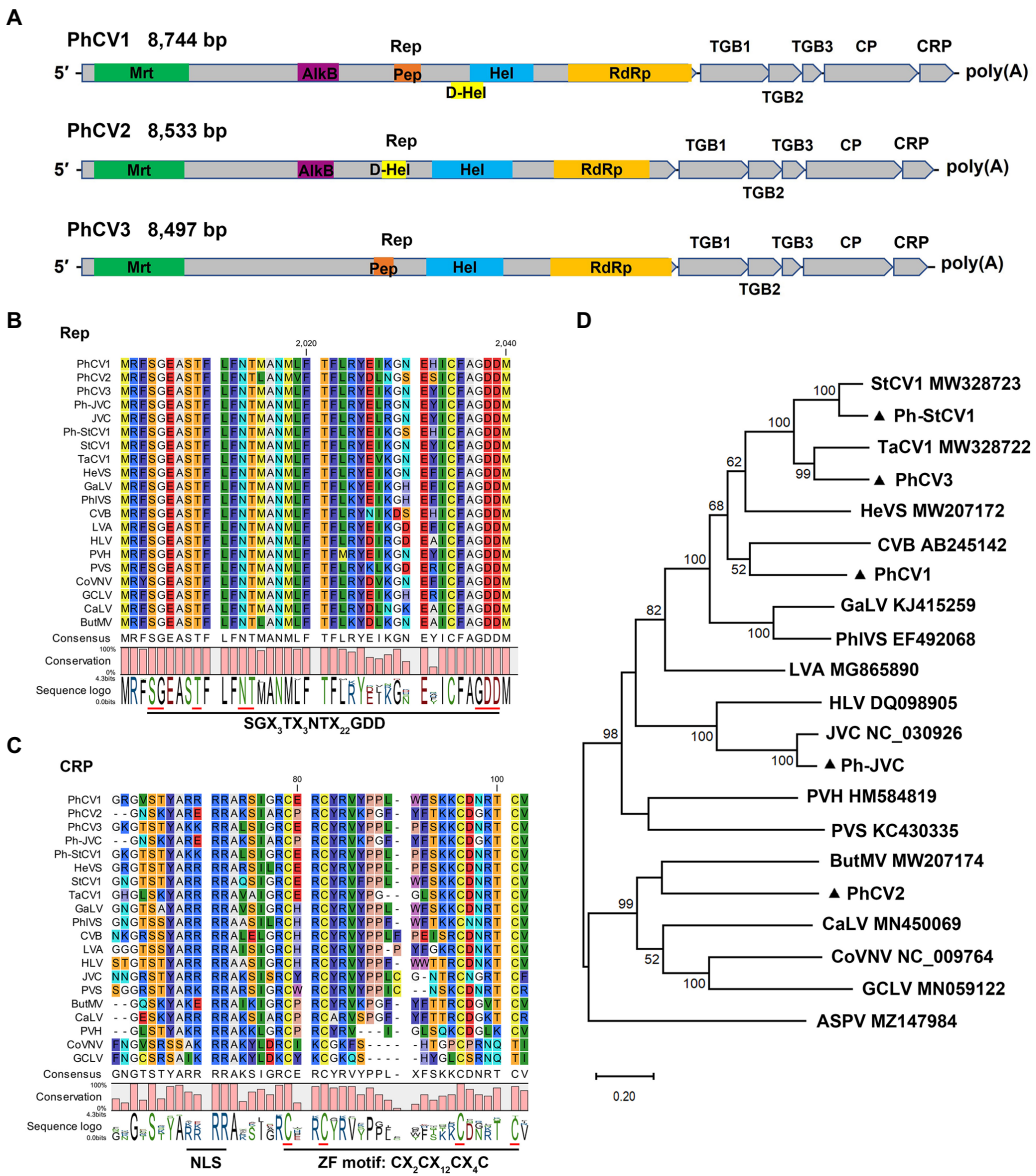
Characterization of three novel carlaviruses, pseudostellaria heterophylla carlavirus 1 (PhCV1), pseudostellaria heterophylla carlavirus 2 (PhCV2), and pseudostellaria heterophylla carlavirus 3 (PhCV3) isolated from *Pseudostellaria heterophylla* in China. **(A)** Genomic organization of PhCV1, PhCV2, and PhCV3. Rep, viral replicase; TGB, triple gene block; CP, coat protein; CRP, cysteine-rich protein; Mrt, methyltransferase; AlkB, alkylated DNA repair protein; Hel, viral helicase; D-Hel, DEAD-like helicase; Pep, Carlavirus endopeptidase; RdRp, RNA-dependent RNA polymerase. **(B)** Amino acid alignment between the conserved RdRp motifs of PhCV1, PhCV2, PhCV3, Ph-JVC, and Ph-StCV1 and 15 selected carlaviruses. **(C)** Amino acid alignment between NLS and ZF in the CRP of PhCV1, PhCV2, PhCV3, Ph-JVC, Ph-StCV1, and 15 selected carlaviruses. NLS, nuclear localization signal. ZF, zinc finger-like motif. **(D)** Phylogenetic analysis based on the amino acid sequences of the Rep of PhCV1, PhCV2, PhCV3, Ph-JVC, Ph-StCV1, and the 15 most similar carlaviruses, performed by the maximum-likelihood method in MEGA X software. Bootstrap values (1,000 replicates) are shown below the branches. The black triangles represent carlaviruses identified in this study.

Carlavirus-related contigs were detected in all five libraries, and carlavirus-related reads accounted for the high proportions (91.40, 88.46, and 54.04%, respectively) of the viral-related reads in the ZR-HB, ZR-GX, and DZ libraries (Table 1). Three novel carlaviruses, PhCV1, PhCV2, and PhCV3, and two known carlaviruses, Ph-JVC and Ph-StCV1, were identified in this study. The genomic sequences of PhCV1, PhCV2, and PhCV3 were 8,744, 8,533, and 8,497 bp long, respectively, excluding the poly(A) tail at the 3′ end. Their genome organizations are typical of carlaviruses and contain six open reading frames (ORFs) that encode an Rep, three TGB proteins, a CP, and a CRP (Figure 4A; Adams et al., 2012a).

Six, five, and four domains were identified in the Rep of PhCV1, PhCV2, and PhCV3, respectively (Figure 4A). Viral methyltransferase (Mtr; pfam01660), viral helicase (Hel; cl26263), and RdRp (cl03049) domains were identified in the Rep of these three viruses (Figure 4A). Alkylated DNA repair protein (AlkB; cl21496) and DEAD-like helicase (D-Hel; cl28899) domains were identified in the Rep of PhCV1 and PhCV2 but not PhCV3 (Figure 4A). The carlavirus endopeptidase (Pep; cl05111) domain was identified in the Rep of PhCV1 and PhCV3 but not PhCV2 (Figure 4A). A conserved motif, SGX_3_TX_3_NTX_22_GDD (X, any aa residue), was identified near the C-terminus of the RdRp domain of these three carlaviruses (Figure 4B). Zinc finger-like motifs (ZFs), CX_2_CX_12_CX_4_C, were identified in the CRPs of PhCV1, PhCV2, and PhCV3 (Figure 4C). Nuclear localization signals (NLSs), including RRRR or KKRR, were identified at aa 51–54 in the CRPs of PhCV1 and PhCV3 but not in the CRP of PhCV2 (Figure 4C).

**Figure 5:**
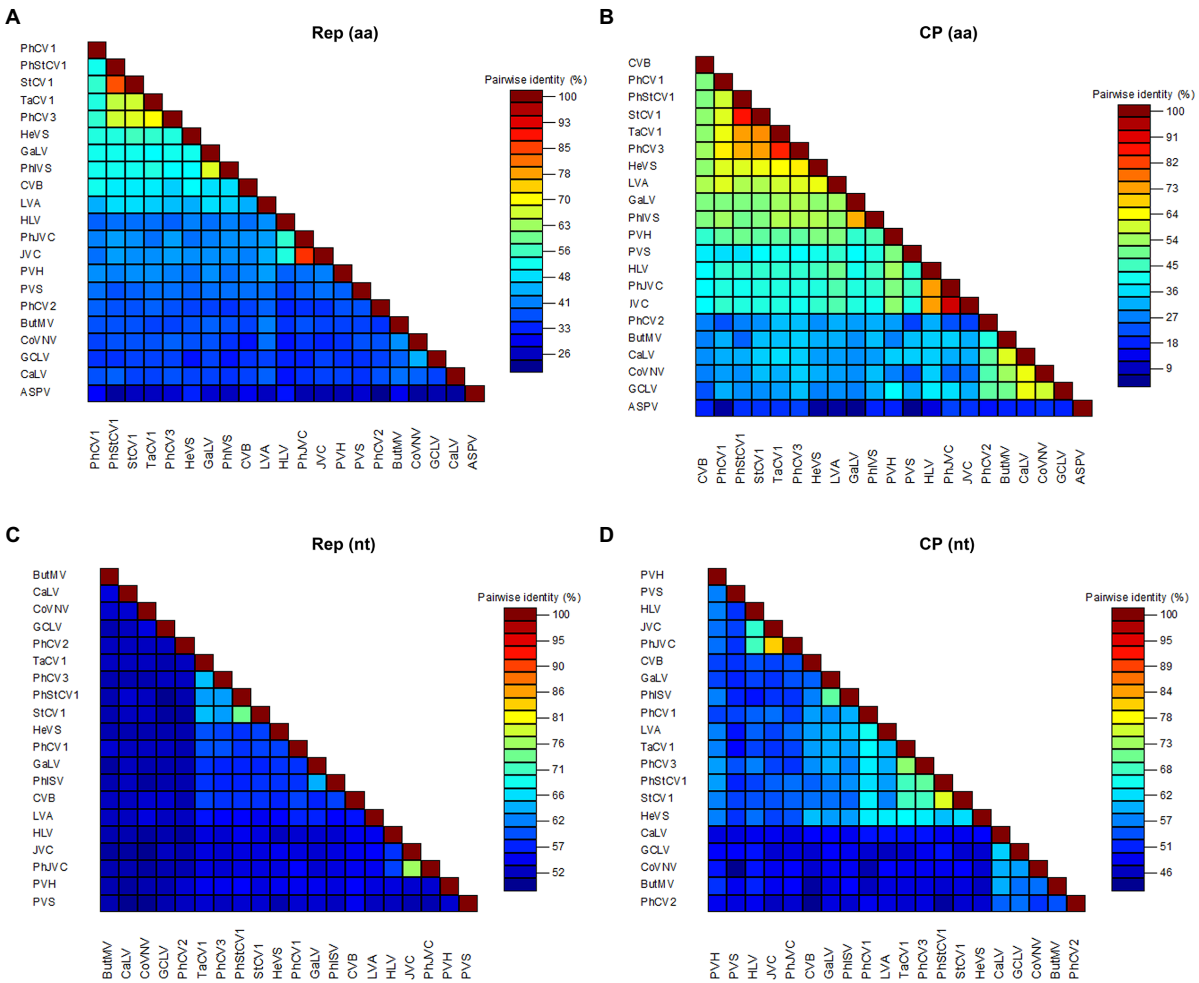
The pairwise identities plot of Reps and CPs in the genus of *Carlavirus* based on amino acid (aa) sequence **(A)** **(B)** and nucleotide (nt) sequence **(C)** **(D)** aligned by MAFFT and displayed by SDT software. Rep, viral replicase; CP, coat protein.

A BLASTN analysis with the nucleotide sequence of genome revealed that PhCV1, PhCV2, and PhCV3 were the most similar to StCV1 (MW328723), Potato virus S (PVS, KC430335), and Tagetes carlavirus 1 (TaCV1, MW328722) with nt identities of 69.95% (55% query coverage), 69.75% (19% query coverage), and 71.15% (87% query coverage), respectively. Pairwise comparisons between PhCV1, PhCV2, PhCV3, Ph-JVC, Ph-StCV1, and 15 of the most similar carlaviruses were performed based on the nt and aa sequences of Rep and CP using MAFFT program and SDT software. The Reps of PhCV1, PhCV2, and PhCV3 shared 53.50–61.30%, 52.80–54.90%, and 53.70–66.90% nt sequence identities and 38.50–56.40%, 36.70–41.90%, and 39.20–72.20% aa sequence identities with Reps in the other aligned carlaviruses (Figures 5A,C; Supplementary Tables S5, S6). The CPs of PhCV1, PhCV2, and PhCV3 shared 47.80–66.40%, 46.10–58.30%, and 50.60–73.50% nt sequence identities and 25.70–67.10%, 24.20–50.50%, and 35.60–85.70% aa sequence identities with CPs in the other aligned carlaviruses (Figures 5B, D; Supplementary Tables S7, S8). The threshold for species demarcation of the genus *Carlavirus* is < 72% nt sequence identity and < 80% aa sequence identity between their CP or Rep. Since the CP and Rep of PhCV1 and PhCV2, Rep of PhCV3 meet the threshold, they can be recognized as new species of the genus *Carlavirus*.

Phylogenetic trees were constructed based on the aa sequences of the Reps and CPs of PhCV1-3, Ph-JVC, and Ph-StCV1, and 15 of the most similar carlaviruses were reported. *Apple stem pitting virus* (ASPV; MZ147984) was used as an outgroup. The phylogenetic tree based on Rep revealed that PhCV1 clusters closely with *Chrysanthemum virus B* (CVB); PhCV2 clusters closely with *Butterbur mosaic virus* (ButMV), and PhCV3 clusters closely with TaCV1 (Figure 4D). A similar result was obtained in the phylogenetic tree based on CP sequences (Supplementary Figure S1). These results demonstrate that PhCV1, PhCV2, and PhCV3 meet the criteria to be new species of the genus *Carlavirus*.

### A novel double-strand RNA virus was identified in *Pseudostellaria heterophylla*

**Figure 6:**
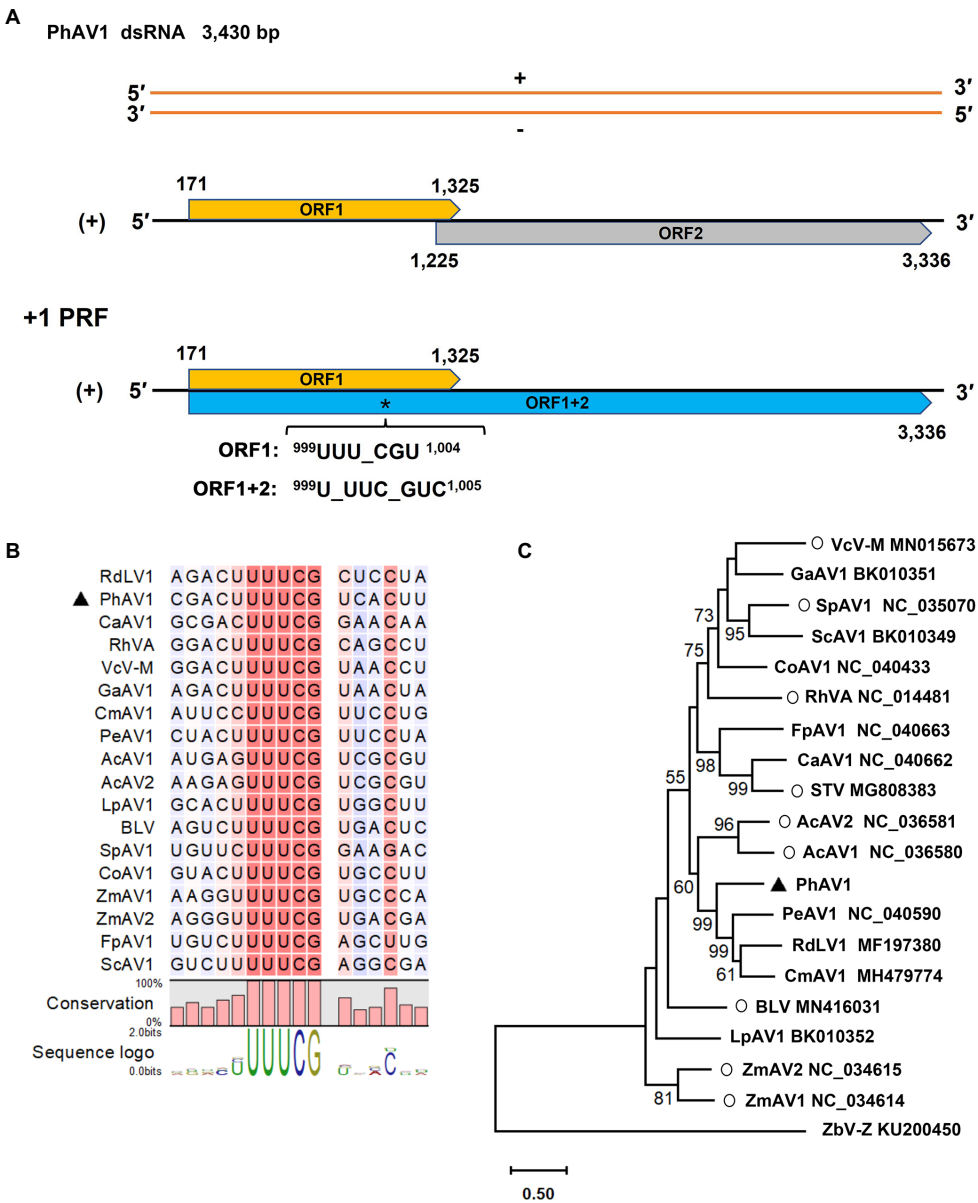
Characterization of a novel amalgavirus, pseudostellaria heterophylla amalgavirus 1 (PhAV1), isolated from *Pseudostellaria heterophylla* in China. Genomic organization of PhAV1 **(A)**, and the putative + 1 programmed ribosomal frameshifting (PRF) motif of PhAV1 **(B)**. **(C)** A phylogenetic analysis based on the amino acid sequences of the RNA-dependent RNA polymerase (RdRp) of PhAV1, nine confirmed (marked with a circle) and nine putative species in the genus *Amalgavirus*, performed by the maximum-likelihood method in MEGA X. The black triangle represents the novel amalgavirus identified in this study.

Amalgavirus-related contigs were identified in all five libraries except for SB. The genome of the novel amalgavirus, PhAV1, was 3,430 bp long and contained two partially overlapping ORFs in its positive strand (Figure 6A). ORF1 (nt 171–1,325) followed a short 5’-UTR of 170 nt and was predicted to encode a CP of 384 aa residues, with an estimated molecular weight of 43.3 kDa. A + 1 PRF motif sequence ^999^UUU_CGN^1,004^ (underline indicates the codon boundary; N, any nucleotide) was found in the ORF1 of PhAV1 (Figure 6B). When the + 1 PRF event occurs, the codon frame changes from ^999^UUU_CGU^1,004^ to ^999^U_UUC_GUC^1,005^, causing the fusion of ORF1 + 2 (nt 171–998, 1,000–3,336), which is predicted to encode RdRp with 1,054 aa residues, with an estimated molecular weight of 120.4 kDa. A BLASTP analysis showed that the CP of PhAV1 had the highest degree of identity (30.73% aa sequence) with that of *Phalaenopsis equestris* amalgavirus 1 (PeAV1; NC_040590), and RdRp of PhAV1 had the most identity (51.01% aa sequence) with that of *Cucumis melo* amalgavirus 1 (CmAV1; MH479774; Zhan et al., 2019). A phylogenetic analysis based on the aa sequences of the RdRp indicated that PhAV1 is in a clade with approved and putative amalgaviruses and in a different clade with *Zygosaccharomyces bailii virus Z* (ZbV-Z), the only species of the genus *Zybavirus* that infects fungi (Figure 6C). Within the amalgaviruses clade, PhAV1 clusters closely with a branch that includes three putative amalgaviruses, PeAV1 (Nibert et al., 2016), *Cucumis melon* amalgavirus 1 (CmAV1; Zhan et al., 2019), and rubber dandelion latent virus 1 (RdLV1; unpublished results; Figure 6C). These results demonstrate that PhAV1 could be a new species of the genus *Amalgavirus*.

### The distribution of PhCV1-3, Ph-JVC, Ph-StCV1, TuMV, BBWV2, and CMV in *Pseudostellaria heterophylla*

**Figure 7:**
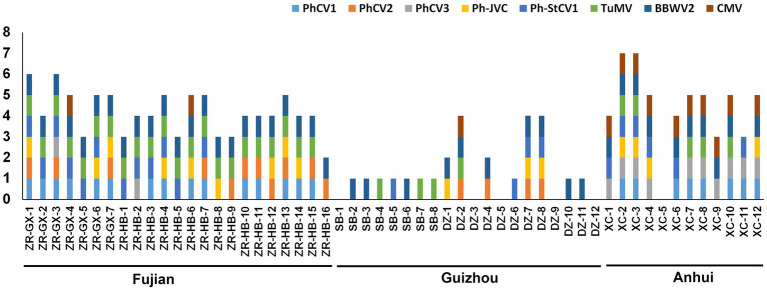
Distribution of PhCV1, PhCV2, PhCV3, Ph-JVC, Ph-StCV1, TuMV, BBWV2, and CMV in *Pseudostellaria heterophylla* collected from five independent locations, including ZR-GX, ZR-HB, SB, DZ, and XC.

To identify the incidence of these nine viruses in different *P. heterophylla* growing regions, 7, 16, 8, 12, and 12, *P. heterophylla* leaf samples were collected from ZR-GX, ZR-HB, SB, DZ, and XC, respectively. Primers that specifically detect PhCV1, PhCV2, PhCV3, Ph-JVC, Ph-StCV1, TuMV, CMV, and RNA1 of BBWV2 were designed and are shown in Supplementary Table S1. The results showed that the detection rate of BBWV2 was the highest (78.18%), followed by TuMV (56.36%), PhCV1 (41.82%), and Ph-StCV1 (40.00%; Table 3). All eight viruses were detected in ZR-GX and ZR-HB in Fujian Province. BBWV2 (100%), TuMV (95.65%), and PhCV1 (65.22%) were present at higher levels (Figure 7; Supplementary Figure S2; Table 3). Except for PhCV2, all the viruses were detected in XC in Anhui Province with BBWV2 (83.33%), CMV (83.33%), and PhCV3 (83.33%) present at higher levels (Figure 7; Supplementary Figure S2; Table 3). Except for PhCV1 and PhCV3, all the viruses were detected in SB or DZ in Guizhou Province with BBWV2 (50.0%), PhCV2 (20.0%), and Ph-StCV1 (20.0%) present at higher levels (Figure 7; Supplementary Figure S2; Table 3). Due to the low content of PhAV1 (Table 1) and the long-term frozen storage of the cDNA samples, this virus was not detected in this experiment.

**Table 3:** RT-PCR analysis of the incidence of PhCV1, PhCV2, PhCV3, Ph-JVC, Ph-StCV1, TuMV, BBWV2, and CMV in *Pseudostellaria heterophylla* collected from five independent locations, including ZR-GX, ZR-HB, SB, DZ, and XC.

Viruses	Regions^a^ (number of samples collected from this region)	ZR-GX (7)	ZR-HB (16)	ZR-GX+ ZR-HB (23)	SB (8)	DZ (12)	SB + DZ (20)	XC (12)	Total (55)
PhCV1	Number of positive samples (Positive ratio)	6 (85.71%)	9 (56.25%)	15 (65.22%)	0 (0)	0 (0)	0 (0)	8 (66.67%)	23 (41.82%)
PhCV2		3 (42.86%)	8 (50.00%)	11 (47.83%)	0 (0)	4 (33.33%)	4 (20.00%)	0 (0)	15 (27.27%)
PhCV3		1 (14.29%)	1 (6.25%)	2 (8.70%)	0 (0)	0 (0)	0 (0)	10 (83.33%)	12 (21.82%)
Ph-JVC		3 (42.86%)	6 (37.50%)	9 (39.13%)	0 (0)	3 (25.00%)	3 (15.00%)	4 (33.33%)	16 (29.09%)
Ph-StCV1		6 (85.71%)	6 (37.50%)	12 (52.17%)	1 (12.50%)	3 (25.00%)	4 (20.00%)	6 (50.00%)	22 (40.00%)
TuMV		7 (100%)	15 (93.75%)	22 (95.65%)	2 (25.00%)	1 (8.33%)	3 (15.00%)	5 (41.67%)	31 (56.36%)
BBWV2		7 (100%)	16 (100%)	23 (100%)	3 (37.50%)	7 (87.50)	10 (50.00%)	10 (83.33%)	43 (78.18%)
CMV		1 (14.28%)	1 (6.25%)	2 (8.70%)	0 (0)	1 (8.33%)	1 (5.00%)	10 (83.33%)	13(23.64%)

a Sample collection regions, DZ and SB represent Danzhai County (DZ) and Shibing County (SB) in Guizhou Province, respectively. ZR-HB and ZR-GX represent Huangbai village (ZR-HB) and Gongxi village (ZR-GX) in Zherong County in Fujian Province, respectively. XC represents Xuancheng city (XC) in Anhui Province.

## Discussion

*Pseudostellaria heterophylla* is one of the most popular traditional Chinese herbal medicines and has been cultivated for hundreds of years. Historically, viral infections have always resulted in important diseases that affect the production of *P. heterophylla*. In 1991, four viruses, including TuMV, BBWV2, CMV, and TMV, were reported to infect *P. heterophylla* (Song and Pu, 1991). To our knowledge, no other viruses have been reported to infect *P. heterophylla* in recent decades. Metagenomics based on NGS can be used to discover the biodiversity of plant viruses in nature (Roossinck et al., 2015). Therefore, we used NGS to detect viruses in *P. heterophylla*. Five samples collected from the different primary growing regions of *P. heterophylla* were analyzed, and the results revealed the presence of TuMV, CMV, BBWV2, five carlaviruses, including Ph-JVC, Ph-StCV1, PhCV1, PhCV2, and PhCV3, and one amalgavirus, PhAV1, in *P. heterophylla*. TuMV, CMV, and BBWV2 are three viruses known to infect *P. heterophylla*, and they have very wide host ranges (Song and Pu, 1991; Bujarski et al., 2012; Sanfaçon et al., 2012; Adams et al., 2012b). JVC and StCV1 are two known unclassified carlaviruses that were isolated from Arabian jasmine (*Jasminum sambac*) and stevia (*Stevia rebaudiana*), respectively. Based on informatic analyses of the genomic features and phylogeny, PhCV1, PhCV2, and PhCV3 were proposed to be new members of the genus *Carlavirus*, and PhAV1 was proposed to be a new member of the genus *Amalgavirus*. These data indicate that a rich diversity of viruses infect *P. heterophylla*.

The incidence of these nine viruses in different *P. heterophylla* growing regions was detected by RT-PCR (Supplementary Figure S2). The occurrence of *P. heterophylla* virus disease was very serious in Fujian, Anhui and Guizhou Provinces with incidences of 100, 91.67 and 75.0%, respectively. Of the 23 samples collected in Fujian Province, 22 were simultaneously infected with TuMV, BBWV2, and one or more carlavirus(es). Of the 12 samples collected in Anhui Province, 11 were simultaneously infected with CMV, BBWV2, and one or more carlavirus(es). A total of 20 samples were collected in Guizhou Province. One virus was detected in 10 samples, two in two samples, four in three samples, and no viruses were detected in five samples. This showed that severe viral infections in *P. heterophylla* were present in all three provinces, particularly in Fujian and Anhui Provinces. BBWV2 was the most common virus in the three provinces with an incidence of 100% in Fujian Province, 83.33% in Anhui Province, and 50.0% in Guizhou Province (Table 3). In addition, TuMV (95.65%) and PhCV1 (65.22%) were also common in Fujian Province, and CMV (83.33%) and PhCV3 (83.33%) were common in Anhui Province. The difference and similarity of pathogens of *P. heterophylla* virus disease in different planting regions may be related to regional differences and/or the long-distance transportation of seedlings.

Like other perennial herbs, *P. heterophylla* can germinate the following year through its seeds and root tubers. To preserve unique characters and the high yield of *P. heterophylla*, root tubers are mostly used for propagation and cultivation. Vegetative propagation using root tubers facilitates the transmission of viruses and viral-plant symbiosis and symbiogenesis (Roossinck, 2008). Viruses can also be transmitted from one plant to another through vectors, such as aphids, which often results in mixed infections. Most of the extant viruses, except for the amalgavirus, related to those identified from *P. heterophylla* in this study have specific vectors for their dispersal. TuMV and CMV can be transmitted by more than 80 species of aphids, including *Myzus persicae* and *Aphis gossypii* which are two efficient and important vectors (Shattuck, 1992; Wang et al., 1998; Palukaitis and García-Arenal, 2003); most fabaviruses and carlaviruses have also been demonstrated to be transmitted by *M. persicae* (Machado Caballero et al., 2009; Belliure et al., 2011; Sanfaçon et al., 2012; Adams et al., 2012a; Wang et al., 2019). As a result, the viral diseases of *P. heterophylla* are serious, and multiple virus infection is widespread. Amalgaviruses are not thought to be capable of efficient extracellular transmission; they are primarily transmitted vertically through seeds to maintain their levels and spread among host plants (Martin et al., 2011; Fukuhara et al., 2020). However, this did not affect the widespread existence of amalgaviruses in *P. heterophylla*. Long-term vegetative propagation could be responsible for the widespread existence of amalgaviruses in *P. heterophylla*.

Three new (PhCV1, PhCV2, and PhCV3) and two known (Ph-JVC and Ph-StCV1) carlaviruses were identified in *P. heterophylla* for the first time. Carlavirus-related reads accounted for high proportions (91.37, 88.45, and 54.03% respectively) in the ZR-HB, ZR-GX, and DZ libraries (Figure 2; Table 1), suggesting that carlaviruses are major viruses that infect *P. heterophylla*. Leaf malformation and whole plant stunting are the types of symptoms caused by carlaviruses (Fujita et al., 2018). The symptoms caused by carlaviruses in *P. heterophylla* could not be confirmed because such viruses usually combine with TuMV or BBWV2 to infect *P. heterophylla*, and they were identified in all five libraries. Previous studies have shown that the CRPs encoded by carlaviruses play critical roles in determining symptoms and pathogenicity (Lukhovitskaya et al., 2013; Deng et al., 2015). The N-terminal region of CRPs, which contains conserved NLS and ZF motifs, determined the types of symptoms exhibited (Fujita et al., 2018). The CRPs of PhCV1, PhCV3, and Ph-StCV1 all contain NLS and ZF motifs, while the CRPs of PhCV2 and Ph-JVC contain the ZF motif but not the NLS one (Figure 4C). The association between symptoms and these conserved motifs merits further research.

Most of the putative amalgaviruses that have been recently identified were discovered by the analyses of a transcriptome dataset. Similarly, we identified PhAV1 in *P. heterophylla* by exploring its transcriptome. BLASTP and phylogenetic analyses based on the aa sequences of PhAV1 RdRp showed that PhAV1 clusters in the amalgaviruses clade. It displayed <51.01% aa sequence identity with the other amalgaviruses, indicating that PhAV1 could be a new species of the genus *Amalgavirus*. The +1 PRF is used in the expression of many genes of viruses, such as amalgaviruses, fijiviruses, and the influenza A virus (Firth and Atkins, 2009; Firth et al., 2012; Nibert et al., 2016). The shift site (^999^UUUCG^1,003^) of +1 PRF was identified in the ORF1 of PhAV1. This could result in the direction (+1) of the frameshifting that is involved in the expression of RdRp.

“BBWV2 exhibits a high genetic variation with sequence variants that are continuously distributed in a wide sequence space compared with most plant viruses. This sequence distribution (genetic structure) is not associated with geographic locations, suggesting long-distance gene flow, and it has been primarily shaped by negative selection and recombination (Ferrer et al., 2011).” Six isolates of BBWV2 were identified in *P. heterophylla,* and the RNA1 of isolates ZR-GX1 and XC1, RNA2 of isolates ZR-GX1 and XC2 shared a low degree of nt similarity (RNA1 < 75.66% and RNA2 < 74.19%) with other isolates in *P. heterophylla* and other isolates in the NCBI database (Table 2). Although these BBWV2 isolates did not meet the criteria for definition as new species, they confirmed the high genetic variation of BBWV2. Alternatively, they provide new genomic references for the molecular detection of BBWV2 in *P. heterophylla*.

In conclusion, this study is the first report of the complete nucleotide sequences of new viruses, including PhCV1, PhCV2, PhCV3, and PhAV1, that infect *P. heterophylla*. It is also the first report of the infection of *P. heterophylla* by JVC, StCV1, and multiple BBWV2 variants. The findings of this study provide useful information for the development of a molecular diagnostic technique to establish a more effective *P. heterophylla* viral disease control strategy.

## Data availability statement

The datasets presented in this study can be found in online repositories. The names of the repository/repositories and accession number(s) can be found at: https://www.ncbi.nlm.nih.gov/genbank/, ON241319, ON241320, ON241321, ON241322, ON241323, ON241324, ON241325, ON241326, ON241327, ON241328, ON241329, ON241330, ON241331, ON241332, ON241333, ON241334, ON241335, ON241336.

## Author contributions

RW and WD contributed to conception and design of the study, collected the samples and conducted the experiments. RW, YL, and SL performed the statistical analysis. RW, KG, YL, and SL discussed the results, drafted and revised the manuscript. All authors contributed to the article and approved the submitted version.

## Funding

This work was supported by the Key project at central government level: The ability establishment of sustainable use for valuable Chinese medicine resources (2060302), CAMS Innovation Fund for Medical Sciences (CIFMS) (2021-I2M-1-032), and the National Natural Science Foundation of China (81873095).

## Conflict of interest

The authors declare that the research was conducted in the absence of any commercial or financial relationships that could be construed as a potential conflict of interest.

## Publisher’s note

All claims expressed in this article are solely those of the authors and do not necessarily represent those of their affiliated organizations, or those of the publisher, the editors and the reviewers. Any product that may be evaluated in this article, or claim that may be made by its manufacturer, is not guaranteed or endorsed by the publisher.

## Footnotes

^1^ https://talk.ictvonline.org/taxonomy/

^2^ www.illumina.com

^3^ http://hannonlab.cshl.edu/fastx_toolkit/index.html

^4^ http://doua.prabi.fr/software/cap3

^5^ https://www.ncbi.nlm.nih.gov/orffinder

^6^ www.ncbi.nlm.nih.gov/Structure/cdd/wrpsb.cgi

^7^ https://mafft.cbrc.jp/alignment/server/

## Supplementary material

SUPPLEMENTARY TABLE S5 Pairwise nucleotide identities of the Rep of PhCV1, PhCV2, PhCV3, Ph-JVC, Ph-StCV1, and the 15 most similar carlaviruses.

SUPPLEMENTARY TABLE S6 Pairwise amino acid identities of the Rep of PhCV1, PhCV2, PhCV3, Ph-JVC, Ph-StCV1, and the 15 most similar carlaviruses.

SUPPLEMENTARY TABLE S7 Pairwise nucleotide identities of the CP of PhCV1, PhCV2, PhCV3, Ph-JVC, Ph-StCV1, and the 15 most similar carlaviruses.

SUPPLEMENTARY TABLE S8 Pairwise amino acid identities of the CP of PhCV1, PhCV2, PhCV3, Ph-JVC, Ph-StCV1, and the 15 most similar carlaviruses.
