# Supplementary material for: Virome of Pseudostellaria heterophylla: Identification and characterization of three novel carlaviruses and one novel amalgavirus associated with viral diseases of Pseudostellaria heterophylla
Source: Front Microbiol. 2022 Sep 29;13:955089. doi: 10.3389/fmicb.2022.955089 (PMC9559581; doi:10.3389/fmicb.2022.955089)
Supplement: Supplementary file 2 [file Data_Sheet_1.docx]

Supplementary Material


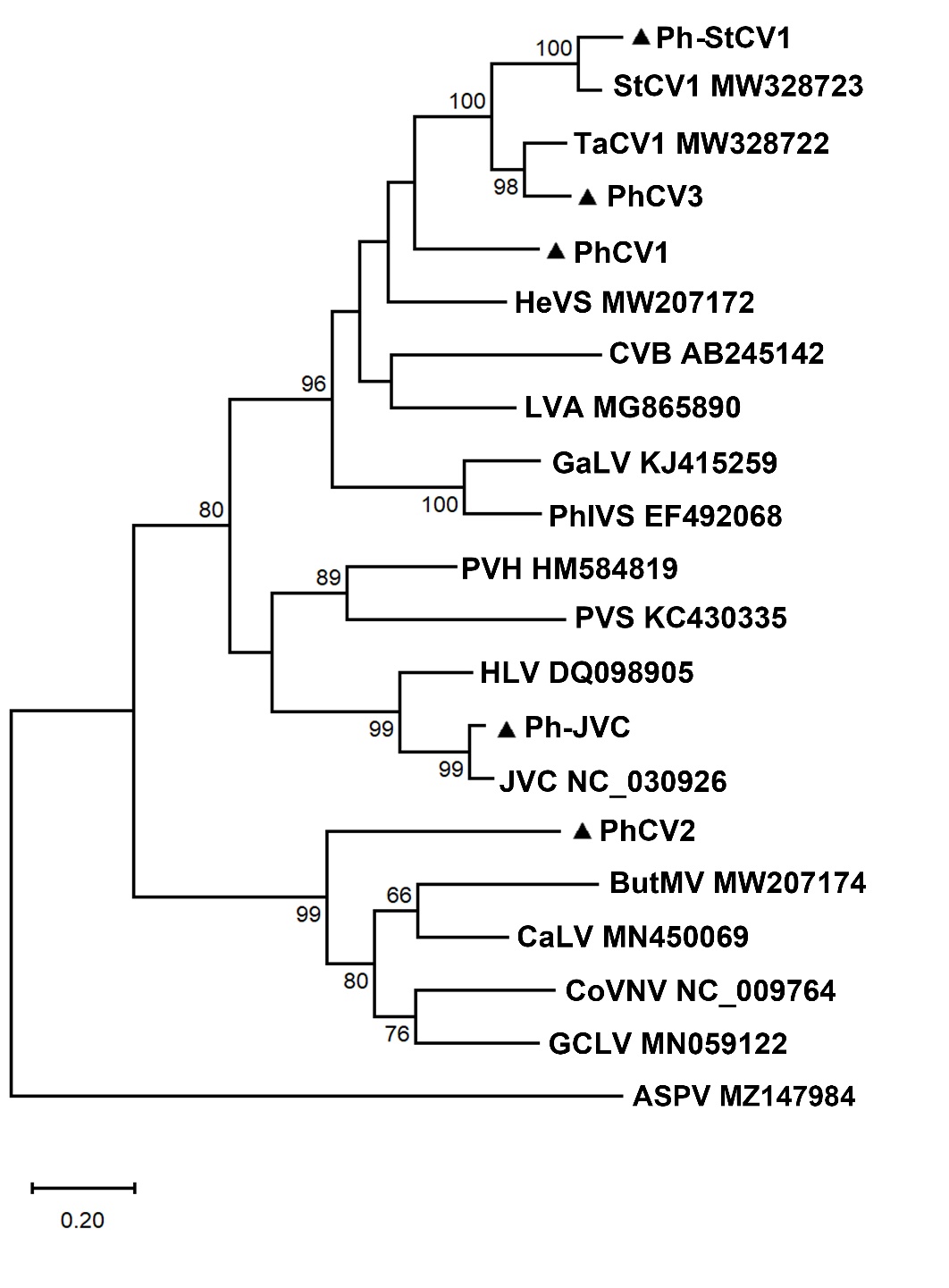


**Supplementary Figure 1.** Phylogenetic analysis based on the amino acid sequences of the coat protein of PhCV1, PhCV2, PhCV3, Ph-JVC, Ph-StCV1, and the 16 most similar carlaviruses, performed by the maximum-likelihood method in MEGA X software. Bootstrap values (1,000 replicates) are shown below the branches. The black triangles represent carlaviruses identified in this study.


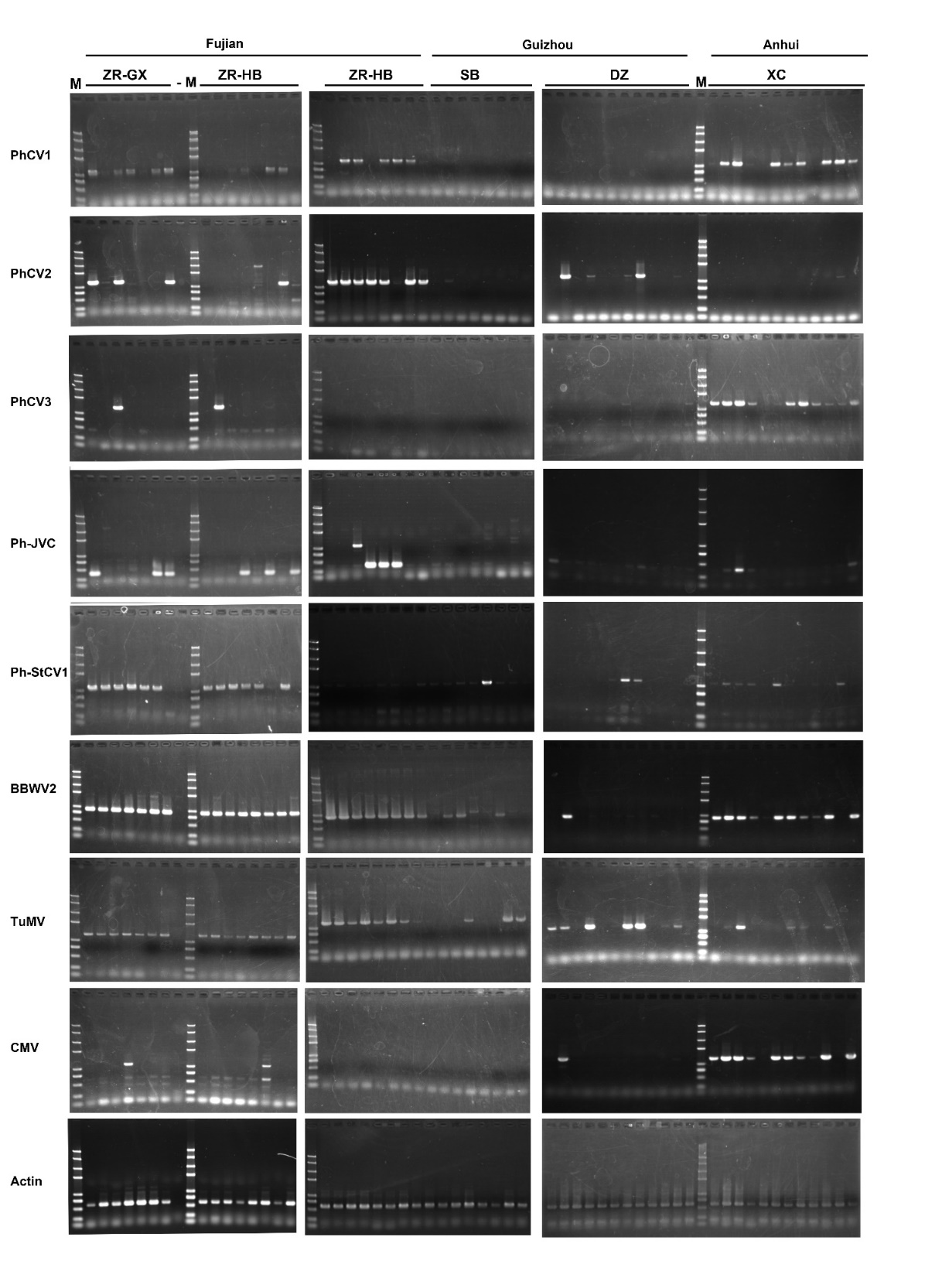


**Supplementary Figure 2.** Agarose gel electrophoresis diagram of the detection of PhCV1, PhCV2, PhCV3, Ph-JVC, Ph-StCV1, TuMV, BBWV2 and CMV in *Pseudostellaria heterophylla* collected from five independent locations, including ZR-GX, ZR-HB, SB, DZ and XC. DZ and SB represent Danzhai County (DZ) and Shibing County (SB) in Guizhou Province, respectively. ZR-HB and ZR-GX represent Huangbai village (ZR-HB) and Gongxi village (ZR-GX) in Zherong County in Fujian Province, respectively. XC represents Xuancheng city (XC) in Anhui Province.

**Supplementary Table 1.** Primers used for PCR amplification, RACE and RT-PCR detection

| Primer name | sequence (5′-3′) | amplifying regions |  |
| --- | --- | --- | --- |
| **carlaviruses** | | |  |
| PhCV1-388R | GATTACGCCAAGCTTCCCGGAATTGGAGGGCTAGACCGAACT | 5′ RACE of PhCV1 |  |
| PhCV1-8058F2 | GATTACGCCAAGCTTCCACTAGAGCTGAGTACGTCGCATACCA | 3′ RACE of PhCV1 |  |
| PhCV1-92F | CCAGTGGCTAAGAAGAAGTTGA | 208-2099 nt of PhCV1 |  |
| PhCV1-1983R | GCTTGTTCTAACGCACCTGAA |  |  |
| PhCV1-1731F | ACACCGAAGGCAAGCACTT | 1847-4116 nt of PhCV1 |  |
| PhCV1-4000R | GTCCTGTTCTTCCTCCACCAA |  |  |
| PhCV1-3757F | CATCAAGGCATCGCAGAGG | 3873-6049 nt of PhCV1 |  |
| PhCV1-5933R | CCTCCTCCTCATCCATTCTGTT |  |  |
| PhCV1-5785F | TTACAAGAAGCCGCAGTTAGTG | 5901-8279 nt of PhCV1 |  |
| PhCV1-8163R | CCACCAGTTATCGCAGAATCAT |  |  |
| PhCV2-721R2 | GATTACGCCAAGCTTACCACATAGTAATTCCGGCGGATACACA | 5′ RACE of PhCV2 |  |
| PhCV2-8047F | GATTACGCCAAGCTTGCTGCCAATGTCAATCGCCGCTTG | 3′ RACE of PhCV2 |  |
| PhCV2-543F | TGTGATCTTGTTCCAGCTTGTA | 543-2463 nt of PhCV2 |  |
| PhCV2-2463R | AACCGCAAGGACCTGTGA |  |  |
| PhCV2-2206F | ATGAACACAGAGCAGAACCTTG | 2206-3848 nt of PhCV2 |  |
| PhCV2-3848R | AGCAACAACCAGCCTCACA |  |  |
| PhCV2-3491F | TGAGCAGCAAGTAATTGTGGTT | 3491-4588 nt of PhCV2 |  |
| PhCV2-4588R | TGCGTCTTCATAATTGGCTGTT |  |  |
| PhCV2-4235F | TGCTGTTGGTGAGGATAGGT | 4235-6570 nt of PhCV2 |  |
| PhCV2-6570R | TGATCGTGTTGTAGTCCGTGAT |  |  |
| PhCV2-6334F | TTGGCTCTCGTACTTGCTCAT | 6334-8146 nt of PhCV2 |  |
| PhCV2-8146R | AGTCAGTCACCACATCTCTACC |  |  |
| PhCV3-488R | GATTACGCCAAGCTTGCCCGTACCTGGTCCTATCCAAACTGGT | 5′ RACE of PhCV3 |  |
| PhCV3-8150F | GATTACGCCAAGCTTAGGTCGTTGTGAGCGTTGCTACCGTGTT | 3′ RACE of PhCV3 |  |
| PhCV3-2660R | TAGCCAGTCCATTCAACCC | 195-2767 nt of PhCV3 |  |
| PhCV3-214F | CAACTTCTATGTCCGCCCT |  |  |
| PhCV3-2492F | ACATTCAGGCGTTTAGCG | 2545-5167 nt of PhCV3 |  |
| PhCV3-5059R | CGAATCTACACAATACGGCGT |  |  |
| PhCV3-7255R | GCATCTCTGTCAACGCATCTA | 4661-7363 nt of PhCV3 |  |
| PhCV3-4553F | AATTCCGAGAAGTGCGTATGG |  |  |
| PhCV3-6766F | TACGAAATCAACCCTGGGC | 6874-8323 nt of PhCV3 |  |
| PhCV3-8215R | CGGTTATCGCACTTCTTGGAA |  |  |
| PhJVC -151R | GATTACGCCAAGCTTCCTCCATTGGCGTTCTGTATGTGAGAGC | 5′ RACE of Ph-JVC |  |
| PhJVC-8322F | GATTACGCCAAGCTTCCGTTGTTACCGAGTGTACCCACCCTTG | 3′ RACE of Ph-JVC |  |
| Ph-JVC-60F | TGTACCTCATTATAACGCACGA | 60-1495 nt of Ph-JVC |  |
| Ph-JVC-1495R | ACGCCATATTCCTCAATCACAT |  |  |
| Ph-JVC-1258F | TTGATGCGGAGCGTGTGA | 1258-2882 nt of Ph-JVC |  |
| Ph-JVC-2882R | TGATGGCAGATCCTGGCATT |  |  |
| Ph-JVC-2705F2 | GCAGGTGGTTGATTGGAAGTT | 2705-4629 nt of Ph-JVC |  |
| Ph-JVC4629R3 | CCACGATCTCTTCAACATCCTC |  |  |
| Ph-JVC-4554F2 | GCAGCAAGAGGATGTTGAAGA | 4554-6390 nt of Ph-JVC |  |
| Ph-JVC6390R2 | TAAGGCGGCGGTGTTTGA |  |  |
| Ph-JVC-6229F | ACCTGATTGGCAACTACATTCG | 6229-8384 nt of Ph-JVC |  |
| Ph-JVC8384R | GAAACAAGTTCTCCCGTCACA |  |  |
| Ph-StCV1-480R | GATTACGCCAAGCTTGGTTTGGTCGCTCGCACAACGAAATCAG | 5′ RACE of Ph-StCV1 |  |
| Ph-StCV1-8236F | GATTACGCCAAGCTTGCTGTGAGCGATGCTACCGAGTGTACC | 3′ RACE of Ph-StCV1 |  |
| PhStCV1-308F | CTGCCACCACTCGTAGACAA | 308-2679 nt of Ph-StCV1 |  |
| PhStCV1-2679R | TCCTTCGCAATGTACCTCCAA |  |  |
| PhStCV1-2490F2 | AAGAATCGCACAAGCACAGT | 2490-4273 nt of Ph-StCV1 |  |
| PhStCV1-4273R | ACTCAAGGCGGTTATCCATCT |  |  |
| PhStCV1-4119F | AGGAGAAGAAGATCGTGGAGTC | 4119-5851 nt of Ph-StCV1 |  |
| PhStCV1-5851R | CGCTTCCACCTCCTCTTCA |  |  |
| PhStCV1-5311F | CGATGCTTCTCAGGACCAGTA | 5311-6943 nt of Ph-StCV1 |  |
| PhStCV1-6943R2 | ATCCTCCGCCTGTAGAATACTT |  |  |
| PhStCV1-6456F | GCTTACTGAGGAGGCACTGT | 6456-8296 nt of Ph-StCV1 |  |
| PhStCV1-8296R | CGGTTATCGCACTTCTTGGAA |  |  |
| **PhAV1** |  | |  |
| PhAV1-247R | GATTACGCCAAGCTTCTGCTCAGCGAGGGAGTCAAGAACATCT | 5′ RACE of PhAV1 |  |
| PhAV1-2878F | GATTACGCCAAGCTTCGGAGTGGCGTCGGATGAGTGGA TATG | 3′ RACE of PhAV1 |  |
| PhAV1-170F | TCATCTGCGTGGAGACCTAC | 186-1807 nt of PhAV1 |  |
| PhAV1-1791R | TCATAACACATCTGCCGATTGG |  |  |
| PhAV1-1434F | GTGTGACGCAACTGAAGAGG | 1431-3215 nt of PhAV1 |  |
| PhAV1-3178R | TTGAAGTCTTGGTTGACCTGTG |  |  |
| **BBWV2** |  |  |  |
| BBWV31-5628F | GATTACGCCAAGCTTCCGCCTATGTGTTCTCCGTCATGCTTCT | 3′ RACE of RNA1 of GX1-BBWV2 |  |
| BBWV31-507R | GATTACGCCAAGCTTGCATTCTCACTTGCCACGAAGCCACAC | 5′ RACE of RNA1 of GX1-BBWV2 |  |
| BBGX1-RNA1-347F | GCATGGACATGGAGCAGATG | 347-2709 nt of RNA1 of GX1-BBWV2 |  |
| BBGX1-RNA1-2709R | AAGATATGCTCGCTGCCACT |  |  |
| BBGX1-RNA1-2602F | GTGGTGAAGCGTTGGATTGG | 2602-5733 nt of RNA1 of GX1-BBWV2 |  |
| BBGX1-RNA1-5733R | TTGCATGGCTTGTGTCAGAG |  |  |
| BBWV32-320R | GATTACGCCAAGCTTGGGCTACGTGTTCTGGGTGCATAACGA | 5′ RACE of RNA2 of GX1-BBWV2 |  |
| BBWV32-2862F | GATTACGCCAAGCTTGATTACGCCAAGCTTACCACTGCGATACATGATGGGTCAGTCC | 3′ RACE of RNA2 of GX1-BBWV2 |  |
| BBGX1-RNA2-1125R | CCGACTAACCATTGACAACTCA | 100-1125 nt of RNA2 of GX1-BBWV2 |  |
| BBGX1-RNA2-100F | ACGAAATTGCTTTCTGGCTACT |  |  |
| BBGX1-RNA2-2891R | TGTATGTCCTCATCAGCCTTAC | 931-2891 nt of RNA2 of GX1-BBWV2 |  |
| BBGX1-RNA2-931F | GCTTCTGTCAATTCGGATGTCA |  |  |
| GX2-15RACE-652R2 | GATTACGCCAAGCTTTTTCCTGTAGACGCTTGCCCAGCTTCC | 5′ RACE of RNA1 of GX2- and XC2-BBWV2 |  |
| HB-15RACE-590R2 | GATTACGCCAAGCTTACCTGATATGTCCGTGGCTGTCTCCTCT | 5′ RACE of RNA1 of HB-BBWV2 |  |
| GX2-13RACE-5538F | GATTACGCCAAGCTTAGAGGAGGTTTGCCAACAACGCAGTTCA | 3′ RACE of RNA1 of GX2-, HB- and XC2-BBWV2 |  |
| BBGX2-RNA1-544F2 | ATACAGCCACGGACACATCA | 544-2369 nt of RNA1 of GX2-, HB- and XC2-BBWV2 |  |
| BBGX2-RNA1-2369R2 | AGAGGTGGCTATCACTGTAAGG |  |  |
| BBGX2-RNA1-2266F2 | ATGAGGAGCAAGAGGAGTTGT | 2266-4475 nt of RNA1 of GX2- and HB-BBWV2 |  |
| BBGX2-RNA1-4475R2 | AATGCCAACTTGAGCTGGTAA |  |  |
| BBGX2-RNA1-2148F | GATCCGTGTGCCAATCAGAC | 2148-4641 nt of RNA1 of XC2-BBWV2 |  |
| BBGX2-RNA1-4641R | TAGACTCCTCATTCGCCTTCAT |  |  |
| BBGX2-RNA1-4318F | ATGAGAGGTTGCCGATTAGGAA | 4318-5733 nt of RNA1 of GX2-, HB- and XC2-BBWV2 |  |
| BBGX2-RNA1-5733R | TGCTTGTCAACCTGTTAATTGC |  |  |
| XC1-13RACE-5309F | GATTACGCCAAGCTTCGAGTCTTCGTTGCGGCGGAGAAACA | 3′ RACE of RNA1 of XC1-BBWV2 |  |
| XC1-15RACE-564R | GATTACGCCAAGCTTGCTATTCTGGACTTGGAGCAACCGACCC | 5′ RACE of RNA1 of XC1-BBWV2 |  |
| BBXC1-RNA1-179F | CGGAAGGCACGCATAGATG | 179-2429 nt of RNA1 of XC1-BBWV2 |  |
| BBXC1-RNA1-2429R | TCAGTTGAGCCACATCCAGTT |  |  |
| BBXC1-RNA1-2118F | CGTATGCCGAATTGTGGTCTTA | 2118-4185 nt of RNA1 of XC1-BBWV2 |  |
| BBXC1-RNA1-4185R | GGCACCTGGCTCTTACAAGT |  |  |
| BBXC1-RNA1-4084F | GAGTAGGTATGTGTCTGGTGAA | 4084-5758 nt of RNA1 of XC1-BBWV2 |  |
| BBXC1-RNA1-5758R | GGCATAGTACACACTACACACA |  |  |
| DZ-15RACE-610R2 | GATTACGCCAAGCTTTCCTCTTGGGCTCCCTCATCCGGGTTT | 5′ RACE of RNA1 of DZ-BBWV2 |  |
| DZ-13RACE-4707F | GATTACGCCAAGCTTAGCCGTTATGCCATGTGCAATCGTGTCT | 3′ RACE of RNA1 of DZ-BBWV2 |  |
| BBDZ-RNA1-386F | GCGAGATTGGAATGAAGGTATG | 386-2200 nt of RNA1 of DZ-BBWV2 |  |
| BBDZ-RNA1-2200R | TATGTCTGATTGGCACAAGGAT |  |  |
| BBDZ-RNA1-2092F | GAGTTCACGATTCCGAAGCATA | 2092-3198 nt of RNA1 of DZ-BBWV2 |  |
| BBDZ-RNA1-3198R | ACACTTGCGTCCTAACATTGG |  |  |
| BBDZ-RNA1-2905F | GCTCGCTTATCGCCATCATAG | 2905-4237 nt of RNA1 of DZ-BBWV2 |  |
| BBDZ-RNA1-4237R | TCGCCTGTCACATACCTACTC |  |  |
| BBDZ-RNA1-3837F | GCTGCTAGTGGTAAGACAACAT | 3837-5011 nt of RNA1 of DZ-BBWV2 |  |
| BBDZ-RNA1-5011R | GTCTTGTCAATGCCATCTGTCA |  |  |
| GX2-25RACE-285R | GATTACGCCAAGCTTAGAAATGCGGCCCACAGCAAGAAACAAC | 5′ RACE of RNA2 of GX2-, DZ-, and XC1-BBWV2 |  |
| GX2-23RACE-2816F | GATTACGCCAAGCTTATGGGCCGCTGGAAATACAAAGCTGGTT | 3′ RACE of RNA2 of GX2-, DZ-, and XC1-BBWV2 |  |
| BBGX2-RNA2-169F | TGTGATGAATCCTGAGTTAGCA | 201-1876 nt of RNA2 of GX2-, DZ-, and XC1-BBWV2 |  |
| BBGX2-RNA2-1844R | AACTGACTGCCAATCCAATCC |  |  |
| BBGX2-RNA2-1529F | TTATCAGACGAGGATGGCAAGG | 1561-3162 nt of RNA2 of GX2-, DZ-, and XC1-BBWV2 |  |
| BBGX2-RNA2-3130R | TTCGCCACTAGGACTTGTCAA |  |  |
| HB2-25RACE-750R | GATTACGCCAAGCTTGCTCCTCAGAGTGTGCCACTTGTCCATC | 5′ RACE of RNA2 of HB-BBWV2 |  |
| HB2-23RACE-3183F | GATTACGCCAAGCTTGGAAGTGGTTGGTCCAGTTGACGGCTT | 3′ RACE of RNA2 of HB-BBWV2 |  |
| BBHB-RNA2-321F2 | AGCCGTTCCTAAAGGATTGT | 486-2161 nt of RNA2 of HB-BBWV2 |  |
| BBHB-RNA2-2020R2 | TGGAGGGCTCATAGTAAACT |  |  |
| HB-25RACE-490R | GATTACGCCAAGCTTCCTTGGGAATGGTATGCACAGGCGTGAT | 5′ RACE of RNA2 of XC2-BBWV2 |  |
| HB-23RACE-3089F | GATTACGCCAAGCTTTGGGTTGGAATGTGCGTGGAAGCAAGAA | 3′ RACE of RNA2 of XC2-BBWV2 |  |
| BBHB-RNA2-426F | CACGCTTGCTGAGTTAATGGT | 548-2013 nt of RNA2 of XC2-BBWV2 |  |
| BBHB-RNA2-1891R | TCCACCAATCAACACAAGAGAA |  |  |
| BBHB-RNA2-1725F | GACATGCGGAATAGGCTTGG | 1872-3342 nt of RNA2 of HB-BBWV2; 1847-3317 nt of RNA2 of XC2-BBWV2 |  |
| BBHB-RNA2-3195R | ATCTGGACCGAATTGACCGTAA |  |  |
| **Viruses detection** | |  |  |
| PhCV1-1564F | TTCTGCTGCTCTGTGCTTATG | PhCV1 detecting |  |
| PhCV1-2696R | GACTCAGCACCACCATCCA |  |  |
| PhCV2-1015F2 | GCATACCAATGAGCCACATCC | PhCV2 detecting |  |
| PhCV2-2228R | CCAAGGTTCTGCTCTGTGTTC |  |  |
| PhCV3-1740F | ACGCACAGCCAATAGTAGATTC | PhCV3 detecting |  |
| PhCV3-3036R | CGCAGAGACAAGACAACCATT |  |  |
| PhJVC-3093F3 | GGATGTGCTGGCTGTGATTG | PhJCV detecting |  |
| PhJVC-3477R3 | ACCTGTGCTACCGCTATTGAA |  |  |
| PhStCV1-6646F | TGGCTCTTACACCTCCTCCT | PhStCV1 detecting |  |
| PhStCV1-7751R | TACAGACGGCACACTCTACG |  |  |
| TuMV8435F | TBGARCCAGARCGRATAGTRTC | TuMV detecting |  |
| TuMV9715R | ACACTGGCTGCTTTAACAAACT |  |  |
| BB-RNA1-1521F | TGGTTGWCWGAGTGYGAYTG | BBWV2 detecting |  |
| BB-RNA1-2420R | TTNGGWGCAAAMGADGTGGC |  |  |
| CMVII931F | CTTCAGATCGCAGGTGGTTAA | CMV detecting |  |
| CMVII2044R | CCGTAAGCTGGATGGACAAC |  |  |

**Supplementary Table 2**  Detailed information of sampling sites of *Pseudostellaria heterophylla.*

| **Sample name** | **Geographical region** | | | **Longitude and latitude** | **Altitude** |
| --- | --- | --- | --- | --- | --- |
|  | **Province** | **County** | **Village** |  |  |
| SB | Guizhou | Shibing | Niudachang | E107.95; N27.13 | 936 m |
| DZ | Guizhou | Danzhai | Zhongying | E107.82; N26.33 | 966 m |
| ZR-HB | Fujian | Zherong | Huangbai | E119.79; N27.19 | 695 m |
| ZR-GX | Fujian | Zherong | Gongxi | E119.83; N27,19 | 501 m |
| XC | Anhui | Xuancheng | Wubian | E118.83; N30.83 | 62 m |

**Supplementary Table** **3.** Next-generation sequencing analysis of five samples collected from *Pseudostellaria heterophylla* grown regions in Guizhou, Fujian and Anhui Provinces.

| **Sample** | **Raw reads (M)** | **No. of clean reads** | **GC (%)** | **No. of contigs** | **Contigs length (nt)** |
| --- | --- | --- | --- | --- | --- |
| DZ | 75.80 | 73,344,192 | 39.5 | 122,037 | 202-9,947 |
| SB | 127.30 | 123,686,642 | 41.0 | 133,562 | 200-8,647 |
| ZR-GX | 61.80 | 59,932,792 | 40.3 | 95,378 | 227-10,252 |
| ZR-HB | 145.12 | 142,685,292 | 41.6 | 277,230 | 200-8,647 |
| XC | 85.60 | 97,108,436 | 39.9 | 97,426 | 203-9,845 |

**Supplementary Table 4.** Pairwise amino acid identities of the BBWV2 isolates identified in *Pseudostellaria heterophylla*

| Isolates | Amino acid identity (%)  Polyprotein/Pro-Pol region (RNA1); coat protein (RNA2) | | | | | Most similar isolate (GenBank accession No.) | Amino acid sequence identity (%) |
| --- | --- | --- | --- | --- | --- | --- | --- |
|  | ZR-GX2 | ZR-HB | DZ | XC1 | XC2 |  |  |
| **BBWV2 RNA1** | | |  |  |  |  | Polyprotein/ Pro-Pol |
| ZR-GX1 | 85.34/89.35 | 85.61/89.58 | 84.48/88.89 | 84.97/87.96 | 85.66/89.35 | LNSY (MN786954) | 85.61/89.81 |
| ZR-GX2 |  | 99.04/99.07 | 92.08/95.83 | 82.71/85.65 | 98.77/98.61 | LN/ (MK116519) | 94.55/96.53 |
| ZR-HB |  |  | 92.65/96.06 | 83.03/85.88 | 99.09/99.07 | LN/ (MK116519) | 94.92/97.22 |
| DZ |  |  |  | 82.97/85.19 | 92.40/95.37 | LNSY (MN786954) | 91.87/96.30 |
| XC1 |  |  |  |  | 82.82/85.65 | LNSY (MN786954) | 80.30/87.04 |
| XC2 |  |  |  |  |  | LN/ (MK116519) | 94.82/96.76 |
| **BBWV2 RNA 2** | | |  |  |  |  | Polyprotein/ CP |
| ZR-GX1 | 80.45/84.64 | 81.30/84.47 | 80.83/84.97 | 80.73/84.97 | 79.68/85.48 | Yunnan (MW271032) | 82.33/85.31 |
| ZR-GX2 |  | 89.57/93.16 | 98.68/98.33 | 98.78/99.00 | 79.07/85.14 | IP (AB018698) | 96.99/98.00 |
| ZR-HB |  |  | 89.57/92.82 | 89.29/92.82 | 79.63/85.64 | PC (MW939477) | 96.05/98.50 |
| DZ |  |  |  | 98.59/99.00 | 79.16/85.14 | IP (AB018698) | 96.99/98.00 |
| XC1 |  |  |  |  | 78.97/85.14 | IP (AB018698) | 96.80/98.33 |
| XC2 |  |  |  |  |  | PC (MW939477) | 81.21/85.81 |

ZR-GX1, ZR-GX2, ZR-HB, DZ, XC1 and XC2 represent the BBWV2 isolates isolated from the samples collected from Gongxi village (ZR-GX) and Huangbai village (ZR-HB) in Zherong County in Fujian Province, Danzhai County (DZ) in Guizhou Province, and Xuancheng city (XC) in Anhui Province, respectively.
