# Supplementary material for: Virome of Pseudostellaria heterophylla: Identification and characterization of three novel carlaviruses and one novel amalgavirus associated with viral diseases of Pseudostellaria heterophylla
Source: Front Microbiol. 2022 Sep 29;13:955089. doi: 10.3389/fmicb.2022.955089 (PMC9559581; doi:10.3389/fmicb.2022.955089)
Supplement: Supplementary file 3 [file Table_3.DOCX]

>PhCV1 [organism= Pseudostellaria heterophylla carlavirus 1] strain TZS, complete genome

GGATAAACAACATACATTTCCTTAATATAACGTACAGTAATTTACAATCACTTACGTTACATTATGGCCCTAACTTATAGGAGTCCACTTGAGGAAAATGTTGCAGCTTATGATTCAAGTGTGCAAGGGGTGATTGCTAGCACTTCAGCTAACTACTACCGGGACGTAGAAAGTAATAATTTCCAATTCTTCAACTTCCATCTAAGCCCAGTGGCTAAGAAGAAGTTGATAGAGGCCGGAATTTATTTAAGTCCGTACTCGGCCCTACCGCATTCGCACCCTGTTTGCAAAACACTTGAGAACAACATCCTGTACAATGTACTGCCGGGTCTTGTAGATAATTCCTTTTATTTCGTAGGTATTAAGAATTTTAAGTTAGATTTGCTTAAACTTAGGAATTCCAAATTGAGTATGTTGAACAAAATCTGTAGATATGTCACCAGTCTAGATAAAGTTAGATACGGGAGTGAATTCGTAGTTCGGTCTAGCCCTCCAATTCCGGGTTTTAAACGCCATTGTGGCCACATAGATGGCCCCACATTAAAGGAATTGGTCCCCGAGGTAGTTGCTAGATCGGCAAAAAAACTCTTCCTACATGACGAGTTACATTATTGGGACCACAGGGATCTCATTAGTTTTCTGGAGGTAGTGAAACCGGAGGTACTGTACGCCTCTGTGGTCTACCCCCCTGAGATTCTTCAGGGGGCGACTAGTAGTCTCAATAAGTGGTGTTACACTTTTGAGATTGTGGGGAGAGATTTCGTGTTTTACCCTGATGGCGTCCATGGTGAAGGTTACCAGCAGCCGCTTAAATGCGGGTACATTCTGAAAGCCAAGACGATCACATTAGGCGATGGTGAGGTCTATCATGTGGATGTTGTGAGTAGTAAATTTGCACACCATTTGGTAGCAATCACCAAAGGGAAAAGTTTAGGACCCACAGTGAGGTCTTTCGGGCCCTTTGAGGCGACTTCATGTCGAGGCTTGGAACCAGTGGTGAGGGATGTGCAACACTGCTTCCCAATATCTTATGAGGTAGTCTCAAGAGTGTACAGGTACCTCCGCACTCTCAAGAAGCCAGATGCGCAATCAGCTATGGCCAAGTTAAGCCAGCTGCTTCCAGAGCCCACGGGCATAGAGATAAAGTTCCTTCAGGACTTTGCTGATTTAGTGATTGGAACCAGCACAATTAACACAATGATAAAGCCGGCTAGGGCTAAGATCTTCTTTGGCGGTTGGCTTCGGAAATTGCCGAAAATCTTGGTGTCGAGATTCAGTGTTGTTAAAGAGCTTAGTCTTGATGAATTCGTCAGTTATATGGAGCCTTATCACTTTCAGGTTGAACTGATGGACATTGGCTGGAACTACGCACACTCTCTTGACCTCTATTCCATGGCTGAGGCTGATTTCGGGGTTGATGTGGTGAAGCTTATTGATGATCGCTTCATTTACGGGACATCATCTGAACTCGTTGACCGAACGCCACAGCCATACACAAGCCTGTTTGCTTGCGTTGAGCGGCGTAGGGAACTTATCGCAATTCCAACCGCGGCTCTGGAGAACTTCTGCTGCTCTGTGCTTATGAGTGAGATCTTCAATCCCTTCATTAGGGAAATTAGCTTAACCAGAGCAAAAGAGGCCATCCTTAGGAAAATATCGCATTCCTGGTGCTTAATAATGCCAAAGAAACTCCTGATGAAGCACATTGTCAGTGGGAATTTCATGGTTTGCCTAATTCGTAGGCTTCGGTCCAGGTACTGCAAAAACCTGAGACTGGACTACGCAAGCTACCCCCTACTGTGGTTCTTGGATCAAGGTAGGGGGTATCAGAAGTACCTTACGGGGTACACCGAAGGCAAGCACTTGATACCAAAGTGGCACGATATTTATGGGGCTTGCGTTTCATCCGTTATTCGAGGGCCACTACCTGCTTTGAGAAAACGGCTCGTGCCTATTGAATATGCTGGCAGGGATTATTTCGCGTGGCCCCACGGTTTGGGAGCCTCTGTAGGGCCTCAAACACCTGCACCTTTAGTGGAGGAGGAATTAAGCCCCATGCCTGATGAGCCATTGTCTGATTCAGGTGCGTTAGAACAAGCCTGCGGTAGCTGCGAAAAGATGCTAGATTGCACCTGTGGCCTACGAATTGAAGTTCGAAATCTGCCACTTGTAAGCATGCATAACTTCAAAACACTAGAGAAGCTACACAATAGGCAGGTTGGCTGGTATTCAATTGATTCAGAGGGGTACTCCTATAACGGGGGCTCGCATGAGAGTTTAGGTTGGCCTGAATGGCTGCCGGTGTGGATGGAGATAAATGGGATTGACCATCAATATTATAATTGCTGCCTGTACCAAGTTTATGCCAGAAATGGGAAGATCGGGTTTCATGCTGATGATGAAGACATCTTCGCCACTGGGAGCAGTGTATGCACTGTAAATTTGGAGGGCAATGCCGTTTTTTCCATTAGGTGCAGCTCAGGTTGCGGACAGATTGATTTGAGCCCTGGTGTGATTTTCACCATGCCAGAGGGCTTCCAGGAGTCCCATAAGCATGCAGTGGGTCACGCTTCCGTCGGGCGTAGCTCACTCACATTCAGGAGAAAGAAAACCACTCAGGAAATTAAAGCTACACCGGTGAGTGAGGAAGAGCCTGCAGCTAGCGGGGATGGCGACGTGGATGGTGGTGCTGAGTCGAACCCCGACTCATCTAGTGACAATGAGTCGGGGAGCTTTTCTGGGAGTTCTAGTGATAGCTCTGGAACCCCTTTGGTTGAACGGTCTTACGGTAGCTTGAGTTACAGCAAGTTGCCTGAGAATGTGTTCTTTAGCACCGTGGATGTGCGGGGCGATGGCAATTGTTTCTGGAGAGTGCTGGAACTGGCCACGGGAATGGCCTGGGTGGAAATTAAAAGGTTGGTCGCGAGGAATCCGCCCTTGGATAAAATCTACCAGGAGAGATTCTCAGAGCAAATGCAAAATGGTGTGTATGCTGAAAGTGAGAGTATGCTATCGGCCGCCTATACTTTGCACGCAAAGTTGATCATTTATGATCAGGGTACCCGGCAGTTGGTAACAATCATGCCCACCTGCGAGGTGACAAGAGAGGTGCATATGCATCTAGACAATGAACATTTCCAATATCTTAAACCCTCGAATGATTGTGTCCTTCAGGCCGTGTCAAAGGCTGTTAAGAGGCAATATAATGAAGTATTTGCGGTATACACCAAAACCTGTGGTGAGGAGGGACTTGACGCCGACATTTGGAGGAAAGATGGTGTTAACCTGGAGGACTTGGATTCGATCTTCAAAACGTTTGATATTGAAGCCACAGTTGTCTGCAATGGGGAAGCACACGTTTACAATGCCGGTGGGAGTTTCAAAGCTTGCTTCACACTGGAAAAAGGGCATCTTGAGTACAACAAGAAAAACCCCGTTGTTGATGTGCCTATCCTGAGGGGGGAAATTGATGCTTTGAGCGTGTCATCTGCATCAATTTGCGAGTTGCGAGCCAGGGGCTCTACAGTGCCATACGTTGCCCGAAAGGCTAGAGGAGAGATCCTTGCTAGGAGTTTCCTTAGCGGGTGTACTGGCGTGCTTAACTCTCAACTGTTCAATGATGAAAGCAGCTTGACAGGGAAATTTCTACATGAAGGGCAAGTGCTGCCTTCGGAAGTTATATGTTTACTAGGCACTTTCGGTGCTGGGAAAAGTACTGTTTTCGCTGACTTCTTCGGAAGGAATCGAGCGAAGGGTATTTTTTACGTTTCACCCAGGCGCGCACTTGCTGATGAGTTCCGGAGAAGGATTGGCCTGTCAAGCAGTTTAGGGAAAAAAGGCGCCAAGAAGGATGAAGTTGCTACCGCTAGCGGCATCAAGGCATCGCAGAGGCATTGGCATGTGCTAACGTTCGAGGTGTTCCTCAAGAAGATGCACCAGGTTAAACCTGGTATGGCACTGTTAATAGATGAGGTGCAGCTGTACCCGCCAGGTTATCTTGATTTAGTACTCTACTGCTTACCAGATGGTGTACACGTGATAGTTGGTGGAGATCCTTGCCAAAGTAATTATGACAATGAAAGCGATAGGGCAGTTTTGGTGGAGGAAGAACAGGACGCGATCAAATTACTCAATGGCCAGAAGTATGACTTCAATGTGCTTAGCAAGCGTTTCAAAAATGCCAATTTCATTGGTCGCTTACCTTGTGAAATAGAGCCAACTAGTTGTGCCGTGATGAATGAGGAACATCTGTTGTTCACAGGTCTTGATGAAATGTGCCAGGCCCCGAATGATTATAGCCGAGTGTTCCTGGTGTCCTCATTCGAAGAGAAGAAAATTGTAGAGTCCTACATAAATGATTGCAAGCCCAAGATTCTCACTTTTGGTGAGTCAACTGGTCTGAACTTCCGGAAAGGTGTGATTCTTATCACAAACGTCTCCAGCGCTGCCGATGATAGGAGGTGGGTGACCGCACTCAGCAGGTTTAGTGAGAACATATGCTTTGTTAATCTCGTAGGGACGTCCTGGGAATTTCTGGCTAAATCATATCGTGGGCGGCCTTTAGGACAGTTCTTGAGCAGGAAAGCTTGCGTGGAAGATCTGAAAAACTTCCTGCCTGGTAACCCTACTTTCAACATTGGTTTCCGCGATAGAGTTGGTAAGGATGAGGGTTTAAAAGAAGACAAGGCGGTAGGTGATCCCTGGCTCAAAGGTATGATCGATTTGTTCCAGATTGAGGATGTCGAGGAAGTCGAGGAGCAGTTAGAGGAGATACAGGAGGAGTGGTTCAAGACGCACCTCCCTGTAGCTGAGCTTGAATCTGTACGTGCGAGCTGGGTTCATAAACTGCTTGCCAAAGAGTTCCGCGAGGTGCGTATGGGCTACAATGTGTCAGAACAATTCACTGATGAGTATGCCAAAGAGAAAGGAAAAGTGCTAACGAATGCTGCTGAGCGTTTTGAAGCCATATATCCTCGGCATAGAGCCAATGACACGGTTACTTTCCTCATGGCCGTAAAGAAACGCTTAAGATTTTCAAGACCAGCCACAGAGAAAGCAAAACTAGTGGAAGCGCAAAATTATGGGGCGTTCCTGCTTAAAGAGTTCCTAGCTAAAGTGCCTATCAAGAAGGGGCACAATGAAGTGATGATGAGGCAAGCCAAGGCGGATTTTGAGGATAAGAAAATGAGCAAGTCGGCAGCCACTATTGAGAACCATTCCGGGAGGTCCTGCAGGGATTGGTTGATAGACATTGGTCTCATCTTCTCAAAAAGCCAGCTATGTACCAAATTTGACAACAGATTCCGGGTAGCTAAAGCTGCACAAAGTATTGTGTGCTTCCAGCATGAAGTGCTCTGCCGTTTCGCTCCGTACATGAGGTATATTGAGAAGAAATTGCATGAAGCCCTCCCATCGAATTACTACATTCACTCTGGAAAAGGCCTTGAGGAGTTAAATGCTTGGGTTATTAAAGGCCGATTTGATGGTGTGTGTACTGAGTCTGACTACGAGGCTTTCGATGCGTCACAGGACCAGTACATCGTCGCATTTGAGTTGGCGATGATGAACTACCTGGGGCTGCCGAGGGACTTGATTGAAGATTACAAGTTTATCAAAACACACCTCGGTTCCAAACTTGGCAATTTCGCCATCATGAGATTCTCAGGTGAAGCTAGCACTTTTCTGTTTAATACTATGGCCAACATGCTCTTCACTTTTTTGAGGTATGAGATCAAAGGGAATGAGCACATTTGCTTTGCTGGGGACGATATGTGTGCTTCCAAGCAATTGATTATCAAGAGGGCACATGAAGGCTTCCTCAAGAAGTTGAAACTGAAAGCTAAAGTCTTCATGGTTGAAAAGCCTACTTTTTGTGGTTGGCACCTCTCGCCTGATGGGATTTACAAGAAGCCGCAGTTAGTGCTTGAACGGATGTGTATAGCTAAAGAAAAGGCTAATTTGGCTAATTGCATTGACAATTACGCCATTGAAGTCTCTTTCGCTTACAAAATGGGCGAGCGGGCATTAAACAGAATGGATGAGGAGGAGGCCGCTGCATTCTACAACTGTGTCCGAATAATCATCAAGAATAAGCACCTTCTGAAGTCGGACATCAAAAATCTGTACTCCGATTTAGAATAGATAGGCTTAGGTTGTAGCCTTAGTATGTGATATATGGAAATAATTATAGAATTAGCTTGTAAATATAAGTTTGAACGCCTAAATAGCAAGTTGGGTAGACCTATCGTATTTCACTGTGTGCCTGGAGCTGGTAAGAGCAGCTTAATTAGGGAATTACTGTGTAAGGATAGCAGGTTCAGGGCTTATACCCTAGGTATTGCAGACCCACCAAATCTGAGAGGCTGTCTCATACATAAGTGGGAGGGTTCCCTCGATTCCAGTAAATTGAATATACTGGACGAGTATAATCTCGAAGCCACTGAAACCAGCCAATTTATAGCAGTTTTTGGTGACCCTATTCAGAGCCAACAGAGTTACACCCTGCGTGCCGATTTTATCTGTAAGTTCAGCCAACGGTTTGGCCGCTGCACAGCAGAGTTGCTAAAGGACCTAGGTTTTGAAGTAGAGGCTTCTGGGGACGACGTGGTGCAGATAGTGGATCTATACAGTGTAGATCCAAAAGATACCATACTCTACTACGAAGAGAACGTTGGTTGCCTACTCCGCAGACACAATCTCCCTGCATTGCACATCAGTGAGGCTATTGGGCAAACTTTCGGTAGTGTTACTTTTGTAACCGCGGAGAGTAAAATCCCAGCTGATAATCGTGCCACCGTCTTCCAGTGCTTGAGTCGCCATAGGACTTCGTTAATGATTATGAGTCCAGATGGCACTTACACCTCCGCCTGATTACACTAGAGTCTTCTTATTTCTTGCTATAGGTGTGTGTTTAGTTGCTTTAGTCCTTGTGTACTCGCGAAGCACCCTGCCAACTGTTGGAGACCCGTGGCATAATCTACCTCACGGTGGTCTGTATCGGGACGGTACAAAACAAATATACTACGGAGCGCCCAGCAAGCTGAATTCATTGGAGAGTAGGTACCGCCTAGTCCACCAGCCTTGGGCTTACGTGCTAGGATTATCCTTAGTAATAGCTGTGATCCATATATATGAAAGTGGTAGGGTTTGCCGGTGTGGTAGAGTGCATTAATGTTGGGCAATAGCCATTTAGTAGGTATCTTGTGTGTCTTAATTTGTGTGTTATTATATACCTTAGTACCTATAGGAAATAATTGCGTTGTAATCATTACAGGGGAGTCTGTAAGAATAAGCTCTTGCCTTTTCGACGAACACTTTGTGGAATTCGCAAAGACTGTGAAGCCTGCCGGTTCCTGCTGATCCCTTAGGTTGTTTCGGGTTTATGTTGAATATCAAAAGAACAGGGATATGCCGCCCAAGGAGCAACCCATTAGCAGTGAAGCGCAAGCTCAAAGTTCTTCCACTGCACCACCAGTGCCGCCAATACCACCGCAGCCTCAGCCACGCCCAAGGCCTCCACCAGAGAGAATTGAGGATGGTCGCACACCAAGTGAATCCGGCAGTGAGGATGAGAGCAGGAATGTTAGGAGGTTAAACGTTCTTGCCAATATGCTAAGGAGGGAGCGCAGTGCCATTCGCGTGACCAACGCCAGCTTTGAAACTGGGAGGCCACCACTACAGCCAACAGAGGACATGAGGGGTGATGTCACAAACATGTACCATCGACCGAGCACAGACATGTTGTGGGGCATCAAGCCAAAGAAAGTGTCGAATAACATGGCCACCACTGAGGACGTTGTGAAGATACAGGTCACGCTAGAAGGGATGGGAGTACCGACTGAGATGGTTCGAGGAGTGCTTACTCAATTATGCATTCGTTGTGCCAGTACTAGTAGCTCATCATACCAGGACCCACATGGTACTTTTGAGTGGGATGGGGGTGCAATCATGTCTGACGATGTAGTCGGAACTGTGAACGATATTGCGGGACTGAGGAGGTTGTGCCGGTTATTTGCGCCCGTTACTTGGAACTACATGCATGTGCATAAAACTCCTCCCTCAGACTGGGCAGCCATGGGCTTTTCGTACAACACAAGATATGCTGCTTTCGATTGCTTTGACTATGTGGAGAATGAAGCTGCTATTAAGCCCGCTGGCGGTGTTGTGCCACGCCCCACTAGAGCTGAGTACGTCGCATACCAAACCTACAAGCGAATGGCTGTTGACAGAGCGAACAATAATGACACATATGGTAATTATGATTCTGCGATAACTGGTGGGAGGCAAGGCCCTGACATTGAGCGCAATATGAACAATGCGAATAACAGGAGGTAATGAAGGAAGCAAGGGAAGCTAGATTTGCACGTGTTGTGCTTGTTACCTTATGTAATAATAGCGGTGTGTATTTGCCTAGGGAAATATTAGATAATATTTGTGTGCGAGCCACACCTTTTTACGTGGGTAGGGGTGTGTCGACCTATGCACGGCGTAGACGTGCTAGAAGTATAGGTAGGTGTGAACGGTGCTACCGTGTGTACCCCCCTTTATGGTTTTCCAAGAAGTGCGATAATCGCACATGTGTGCCTGGTATTTTTTATAATGTTAAGGTGCATGATTACATCCGTTGGGGAGTAACTGAGGTGATACCCCACCCCGGATATAATTTTTAAATGCTGCCATTAAAACCTAATTAATGTATGAGTGTGTAGCTACAAATAAATAAAAAGTTTTAAATATTTTTCCTT

>PhCV2 [organism= Pseudostellaria heterophylla carlavirus 2] strain BL, complete genome

GGATAAACCAAACCAACTCAAAATATTACAGCTTTAATCTAAACAATCAAATCTGTAACATCATGGCACTAACTTACCGTAGTGCAATTGAGGAGGTTTTGACCAAATTCACGACTGCTGAGCAGAGTCTAATTGCTGAACCGGCTATAAAGAATTATCGGGATATCGAAGCTAATGAGCACAGATTTTTCAATTTTTCTATGAGCCCGCTAGCAACGGAAAAACTATTCAAAGCAGGTATTTACCTTAGTCCATTCTCTGGGGTGCCGCACTCTCACCCAGTTTGCAAAACCCTGGAAAATTATTTTTTATATAAAGTGCTACCTAATCTTGTTGATAATACATTTTATTTTGTAGGTATAAAAGATAGTAAGCTTAACTTTTTAAAAGAGAGGCACAAAAAATTAGATTTAGTTGGATTAATTAATAGGTATGTTACAAGTGCTGATAAGATAAGATATAGCTCTGATTTTGTGTGTAGTAAGGCAAAGCTTAATGCTGATCCTAGAGTTTTCGAGGTCACAAGTAAAGAAGCAACTTTATGTGATCTTGTTCCAGCTTGTATACTGCAGAAGGCCCGTAATTTGTTCATACATGATGAACTCCACTATTGGAGTGTTGGAGCTTTAAAGAAATTCCTGGGCGCAACAAAACCGAACAAAGTCTTGTGCACTATTGTGTATCCGCCGGAGTTGCTGTGTGGTGCAAAAGAAAGTTTGAATAAGTGGTGCTATGAGTTTAATGTCAGCGGAAAGAATTTTGACTTCTTTCCTGATGGCGTGCGAGCTGAAGGGTATAACCAACCCGTGCGTGGCGGTTTTCTCTTGCAGTGCTCAAAAATACGTTTGGATGATGGTAGCATATACTGCGTTGATTTAGTCCACAGCAAATTTGCTCACCACTTGATATCTATCACCAAAGGGGATGCAATAGTGAGAAGTGCTAACCATTTCAGCAATTTCGATGCATGCACATCTAATGGAATTGCAAACTTGGGTTATGGTTTAAGGCCTTGCATACCAATGAGCCACATCTTAATCAGTCGAATCTATAGGTATTTGAGGACTTTACTAAAACCTGATTTACAGTCTGCAATGGCCAAACTCTCGCAGTTGAAAGCAGACCCGACTGGTTTTGAAATTAAATTTACGCAAGAATTTGCAAGCCTAGTCATTAACACTCCCAATGTTAAAACAATGCTAGATGCATCATATCTAGATAAAATGCAAGAGTGGATAGCTGGGAATGTGCCAAATTTGGTGAACCGGAAATTCAAAATACTGCGAAGAATGTGCTTGGACACATTCATAGGTTCTCTTGAGGAATTCAACTTCTGCGTACCCCTAAGGGAGATGGCTTCGGATGTTGATTTCATCACGGGCGAGCTTTTCCCTTTTCTGCCCGAGGAAGGGACTATCACACCAGAATTGATAATGGATAGATTCATGACTGGCTTGCCTAATTTATGCAACCTAGAACGTGCACCAAGAGGGTACTATCTAGAGCCTTACGTTGACTATGTTGAGTGCGATAGACCTTTACTCATAAAGACGCTGACAAGAATCTATGTCTCAGCAATGGGTAGGCAATCACAATTGATAATGGATTTGAAAATGCTCGAGGGTGATTTTGGCCACTACCTTGATCTTAAAATTGCTGGTGTGATAAGTATAAGAGCTCTTCTAGGCCCCGAGGGTTTGGCTTCCCTTGCTAATAGAATTAGGCCTTGCTGTTTGAGGCTAAGAGTTAAGCACTTCGCAGATGCCTCAATCTCTTGGTTTACTTCACGAGTGCGGAGTAACCAGTTGTATTTATGTACTGCACCGGATGGAACTAGTGCTTCTAATTCTTTCAAAACAGGGTGGATGTCCGTTGTTTGTGAAATTTCAAGTATGGGAGCAGAGGGTAGCAGCCTAATGATAAAGCCCGAGTTTAATGAAACAGGAAATTTATCCAATTCTGGGGAGCAGCAAGGTGAAGCTGCTAAGATGATTGGGGAATTAATTGAAAGTCCGCATTCTGATTTGGTCTTTCCCCAGCCCCCTAAAGTTTTTGATGAATTCTCACTGGCTTGTGGTATTAGTATACCAATACAAGAGATCGTTGGCGGTGAATATCTGTCTTTTGATTATAAAGATCAGTTGAAAGGTCGAAGGGCTGTGTTGTATGCTAAAACGGCTGGTGCAATTTATACTTATCCGGGCTATGAACACAGAGCAGAACCTTGGAACGAAGGATTTTCCATTTTCCTTGAGCTTAACGGTTATGATTCAGATTACTACAATAGCTGCCTGGTTCAGGAGTATGATCAAGGGGCATCCATCGGCTTTCATTCGGATGCGGAAGCATGCTTAGTCGCCGGTAGTAAAGTTCTAACTGTTGTGTTGAAGGGTAGCTGCAAGTTCAAGTTTAAAGGATCCTGCTGCCAGTTGACCTCAAAGAAGATTACAGGTCCTTGCGGCTTTGAGCAAGGTTTGGGCTTTCAAGAGAACCACATGCATTGCATATCTGAGTGCACTCAAGGTCGGCTAACGTTGACTTTTAGAAGGATGATTGAGGTCGTCTCAATTGAAACTTCCATAGTTGATACTGAGGCTGCACCTGATTGTTGTGTTAATAAGGACGAGGTTATATCATACGCCATCAATGAAGTTAATGTGCAGATCTTGAAGGGCAGGCCTGACGACAAATGGAGGGAAATACCAGTGGAGGGTGATGGTGATTGTTTCTACCACTGCCTAGGCTTAGTGCTGGACATGGATGCTACAACAGTTCGGAAGATCCTAAGGGCGAAATTTTTGGTCCACCCCACCGATTTAGCATTTAATTTGGATATGTTAGATGATGGTGTTTATGCCGAGACTGAGCAAATAGCCTTTTGTGTCCAAATCTTCGGAGTGGATCTGAATATTTATGAAGAAAGTGGCTTTTTGCATATCTACAAACCGAACATTAGCAATGCGTCTCTATCATTATCACTTGTGGGGGAGCACTTTACGGTTCTTGTTAGAAGTGGCGATTGCTTGGCAAAGGCGATTGCTAAAGCTACAGGAAAGGACATTGAACTTGTTGTCAGAGCTATGTGCAAGTTCGGCCTGGAATCTGAGGTAGCGCTTTTGTCGAACTTGGGTGCCTTAAGCGAAATCTTTGAGCGTTTTGGCATAGCTGCCACAGTACGGACCGAGGGTGAAACTGTATTTCTAAATGAAAACGGCAGCATCCATGCAGTTTTCTTTCTGGGCCCTAACCACATTGAGTTTGAAGAGAGTAGCTCTGGCCTTTTAAGATCAGGTAATGTTCCTGGAACGTTACTGCCTAAAGGGAGGGCAACCATTGTTGCCTTAGAGGCTGCTGGCACAAAGCTTCCTTATGTTGCCTCTAAGGAAAGAGCTACAAACCTCGCAAAGGCCTTGCACGAAGGTAGGACGGGAGCTATATGCTCCGAACTGTACTTCAATAGGAAAATGATTGAAATTGAAAGGGATGTTGGTGAGCAGCAAGTAATTGTGATTGCCGGAGTTTATGGTTCTGGCAAGAGCTATTTACTTAAAAAAGTTTTTGAGTCGAATATTGGTTGCAGGCTCTTCTATGTATCACCGAGGAAAATCTTAGCTCAGGACTTTTCTGTTGACGTTGGCTGTTCAGTCAGAGACGAGAGCGGGCAGATTGTTAAAATTAAGGGTAAACACCCTGAATGGAATATCTTGACCTTCGAGTCTTTCCTTTTAACCACTAGCAAGGTGCGAGATTACGACGTCGTGGTAATCGATGAAATTCAGCTCTATCCACCTGGTTACTTGGATCTTGTAATACTGAATATGCCTAGACATGTGAGGTTGGTTGTTGCTGGGGATCCGGCCCAAAGTAGGTATGATAATGCAGGGGACCGGAATTATTTCACTGGTGTTGACCCTGATTTTATAAAGCTGTTAGAGGACTCTACTTACCGGTATGTCATTCAGAGTAAGCGCTTTCTCAATGGTAATTTTAAGGGTAGATTGAACTGTGCGCTTTCTAACTCCTTCAATGGTAACGATGAAGAGTTCATCTTCTTGAATAACTTCATGTCTCTCTGCGAGTTGGACTCCAGGGTTGAGGTCGTACTAGTTTCCAGTTTTGTGGAAAAGAAGGCTGTTCGCGCTGTGGTTAATGAAAAGGTCAAAATTTTGACTTTTGGAGAAAGTACGGGTTGCACCTTCAACCGTGCAGCACTCATTGTCTCGAATCCATCACTTGCGGTTGGCGAGGATAGGTGGATCACTGCGCTATCAAGGGCTAGGATGCAACTCATAATCGTAAATTGTCTTGATACGAGTAATGAGATACTGCCTGAAGTGTTTAAAGGGAGATCACTTGGCCATTTCCTTGATAGAACAGCTGGACCTGATGTGCTGCTAAAGTTATTGCCTGGTAATCCTATTTTCTCGAATGAATTTCATCAAAAAACGGGCCGCAATGTTGGCAAGGTTGAAGAGAAGGTTACTGGTGACCCCTGGTTAAAGAGTGTTCTTTTTCTAGGTCAGGAATCTGATGTCTGCGAGTATGAAGAAGCACTCGAGATTATGCAACAACCAATTATGAAGACGCATCTGCCAAAGTGTGAGATGGAGGGGGTTAGAGCAGAATGGGCTCATAAGATATTGGCGAAAGAGTTGAGAGAAAAGAGGTATGGTATGATGGTCTCAAATCAATTTACTGACGATCACTCTAGGAATAATGGATTTAAGTTAACAAATGCTGCTGAGAGGTTTGAAGCTATTTATCCGAGGCATAGGGGATCAGATTCTGTCACTTTTCTAATGGCAGCGCGAAAGAGATTACGGTTCTCAAATCCGGCTAAGGAGTGCGGAAAGTTGAACAGTGCTAGAAAGTATGGCCCGTTCCTCTTGAAGGAATTCCTCAAGAATGTACCGATTGTGGCTGCCCATAATAAGCAGTTCATGGCTGACAGCGTGCGAGATTTTGAGGAGAAAAAGACGTCGAAAAGCGCAGCTATAATTGCTAACCATGCTGGCAGATCTTGTAGGGATTGGCTGATTGACACTGGCTTGGTTTTTATGAAGTCACAACACTGCACTAAATTTGACAACCGATTTAGGGATGCCAAAGCTGCCCAAACTATCGTTTGCTTTCAGCACTCTGTTTTGTGCAGATTCGCACCCTATATGAGGTATATTGAACGCAAAGTCATGGAGGTTTTAAAGCCTAACTATTACATCCACAGTGGAAAAGGTCTTGAAGAATTAAACAAATGGGTTATTAGTAGTAAATTCAACGGATTAAGCACGGAATCAGATTATGAGGCTTTTGATGCTTCACAGGACCAGTATATCATGGCTTTTGAACTTGAACTTATGTCATATTTGGGTCTGCCTGCTGACCTGATTCATGATTATATCTTCATTAAAACGCACCTAGGGTCTAAGTTGGGAAACTTTGCTATCATGAGGTTTTCAGGGGAGGCGAGCACTTTCCTATTCAACACATTGGCTAATATGGTGTTTACTTTCTTAAGATATGATCTAAATGGATCAGAGAGCATATGCTTCGCCGGTGATGATATGAGCTCCTCAACTGCGCTCAAAAAGAAAACCGAGCATGAGGACTTTTTAGGCTTGTTAAAATTAAAGGCTAAAGTGCAGATAACCAAAAACCCAACATTCTGCGGGTGGAACTTGACGCCTATAGGTATTTACAAAAAACCTCAATTGGTCTATGAACGTATGTGCATCGCTAAAGAAACCGGTAATCTCGCAAACTGCATTGATAACTATGCGATAGAGGTATCATTTGCCTATCGATTGGGGGAATTGGCTGTGAACACAATGACTGAGGAGGAGGTCAAAAATTACTATAATTGCGTTCGGGTCATTGTTAAAAACAAGCATTTGATGAAGTCGGACATAGCCTTACTTTTCAAGCAAAGTGATTTGGCTTAGGTTTTTGCCTAGTGTTTGATATTTATGGATGTGCTTATTAAACGATTGTTAGATAGTGGTTTCGAACGTCATGTTGTAGAAAGCCGTGGTTACTTGATAGTTAGCTGTGTGCCTGGTGCTGGTAAGAGTACTTTTATTAGAGACCTGATAGCTAGTGATTCCAGATTTGTGGCTTGCACTTTTGGAAAGCCTGACAAGATATCCAATGCAGGCAATAGAATTTTAGACGTTAGAGCCGTGGAAGATTATAACGGGGCGTACCTAATTTGTGACGAGTTTCAAGAAGGCGATTGGAAAGCTTTGAAAGCCTGCTGCTTCTTCGGGGATCTTGAACAATCATTTCTAGATCAAGGCAATATAAAGCCAAATTTTACTCTTACAAAGACACTGAGATTTGGCTCTAGTACTTGCACATTATTGCAATCTTTTGGCTTTAAGATTACTTCCGAAAAACAAGACGAGGTGTTAATTAACAGTCCGGAGAACGCAATTATTCATCCAAATATCATTGCCATAGGAACGCAAGCTGAAAAATTGTTGTGCTATTACAGATTGGAGTTCAAACGGCCTGGAGAAGTTAGGGGTTGCACTTTTGACAAAGTTACACTTCTCACGGACTACAACACGATCACTGAAGAATTGAGGAGTGAATTCTACGTTGCACTCACGAGGCATCGTGCATGCTTAGAGATTGTTTGTCCTGATGCCACTTTTGCCCCCGCCTGACAATACTAAGGCCTTCTTTGCATTGGCCGTTGGAGTATCTGGAGCTCTGTGTCTACATTTTCTGACGCGATCAAACTTACCCTTTTCTGGGGATAATATTCATAGCTTGCCGCACGGGGGGAATTACGTTGACGGCACAAAAGCTATTCGTTACAATAGAGCTAGCGAACAACAACCAAAGTCACCTTTGCATTTCGAATTAAGCAAAGTACTAATTGTCGCTTTCATTCTAGCTTCAGTTTGCTTACTAAAATACCTGCATAATCCAAGAAGTAGATGCAACCGGTGTGGAAGAGTCCACTGTTAATCGGAGTTTGCACGTTCATCTTTTGCTTCATCGTACTACGTGTTTTAGATTCTGGTGGCCAAGCCGATATTTGCCAGATAATCTTGACAGGTGAATCTTTAAAGATTTTGAACTGCCAAGTTAACGAGCACTTAGTACAGATAACGAAAAACCTCAAAATACCTCCATTACCTGAAAGTTTGGAGCTTTAGGTTTACAGCATTTGTAGTTGATATACAGAAAGAGTTCACCACATAATCATTCAAGATGAGCAATGCGAGCGACTTTGACAAGACTGGCATGAACCCGGCGCAGATAGAGAACGAGACAAAGCGGAGGCAAGCACGAATGAATGCCGGGCTCCCACCTGATAGTCCTAAACCTGCTGTTGAAGCTGTGAAGGGTAATTTTGTCAATGATGTTGATCTTAGAACACTAGAGAATTTATCCGCTGAAAATGAGAAGTACAAAAGCAGGTTTGAGCAGTTAGTGGAATTTCAGAAGAAAAATCTCACCTCCAGTGAAGTTAAAAATATGGGCTTTGAGAGTGGTAGGCCGCCAGCCAAACTGTCAGACAAGTTGAAGGGTGATAAGTCTGATATGTTCACTAGACCTAGTCTAGACGCTCTTTACGCGCTCAATCTAAAGGTTGAGAGTGTGGCTATTGCTACTGCTGAAGACTTTGCTAAAATATCTGCTAAAATACAAGGGATGGGCTGGCCGGCTGAAGAGATGGCTCCACTTTTCTGGAGGATTGCAAGACATTGCGTTGATAATAGCTCTTCCCAGTATCTTGATCCCAATGGGACTTTTGAGGTGCAAGGCTGCACTCTTACCAAAGATGCTGTATTGGCCATCATAAAAGATCAAGTTACTTTAAGGGCTTTCTGCAGGGCGTTCGCACCAGTGATGTGGAATCAAATGATCAGCAACAAGATGCCACCGCAGAACTGGCAGAAAAAGGGCTACACGGAGAATACCAAATATGCTGCTTTTGACACTTTTGATTATGTGCTAAATCAAGCAGCTATCCAGCCTTTAGAGGGGCTCATAAGGGTCCCAACACCTGAAGAGCATTTAGCTGCTAATGTTAATCGTCGTTTGGCTATCAATGGATCAAGGAGGAATTCTCGTTATGCCTCTTACAACAGTGAGATCACTGGTGGCTTGAATGGTAGAGATGTGGTGACTGACTTCTCAACTGCAAATAGTACTGATTGATGAAGATTCGCAAGAGAAGATTGGAGAAAATATTAAGTATTGTATTCCGAACAAAGACTCGATCACCGAGTCATGTCTCGTTTATAGATGTTATAAGTGTGATTATAAGTAAAATGGGTGTTGAACCTGGCAACTCTAAGTATGCTAGAGAACGTAGAGCTAAATCCATAGCCCGATGTCCTAGGTGTTACAGAGTTAAACCGGGGTTTTATTTTACTAAAAAGTGTGATGGTAAGACTTGTGTGCCTGGGTTAAACCATAATTCTAAAGTCGAACTATATATTAAAGTTGCTTCTAAGACCTAAATAATAAATAAAGAAGAACGTATAAAATATTTCCATGTTTTAATATATTTTTAAGG

>PhCV3 [organism= Pseudostellaria heterophylla carlavirus 3] strain TZS, complete genome

GGATAATCCAAACAAACTTCCTCTAACATACAGTAACTAAACATCCGTTCAAATACTGTACGTACCATGGCACTCACTTACAGGAGTCCCTTAGAGGAAAACTTTGCATCGTATGATTCAAGTGTGCAGGCTGCCATTGCAAGCACATCAGCAACTTACTACAAGGAGCTAGAACAGGAAAATTTCCGCTTTTTCAACTTCTATGTCCGCCCTGAGGTCAAGAAGCATTTGATAGATGCGGGGATTTACATTAGCCCAAACGCTGCGGTGCCCCACTCACATCCAGCTTGCAAAACACTTGAAAATCACTTTCTGTATATAGTTCTGCCACCCTTAATAGATAACTCTTTTTTTTTTATAGGTATCAAAGACACAAAGATCAATCTACTCAAGACTAGGAAGACGTCTTTGACTATGGTAAACAAACTAAATAGATATGTAACCAGTTTGGATAGGACCAGGTACGGGCCAGAATTTGTCATACGGAAGTCAGGGCCTATACCTGGCATGAAAAGACACCAGCCAGCGCTGCAAGGGGTAACATTGCGGGACCTAGTGCCGCCTCTAATGGAACAGAGTGCAAAGAATTTGTTTCTGCATGATGAACTTCATTATTGGAGCCACAAGGATCTCATAACTTTTCTAGAAGTGCTGAAACCGAGCATAATCTACGCGACCTTTGTTTACCCTCCAGAGATTTTAACTGGCTCCACTTCAAGTCTGAATAAGTGGTGCTACACCTTTGAATTGGTGGGTAAGGATATTATGTTCTACCCCGATGGAGTTAGAACTGAAGGCTATCAGCAGCCCCTCGCATGTGGCTATCTTTTAAGAACAAGACGACTAGTGCTGAATGATGGTAGTTACTACAACGTGGATGTGGTCCAAAGCAAATTTGCCCACCATTTGATTAGCATCTCGCGAGGCATGGAAGCAGGGCCTTCAATCAGATCCTTTGGGCCCTTTCAAGCAACCTCGTGTAAGGGGCTTGAGCCGCTTTTGAGGAACGTTTCAAATTGTTTCCCAATTTCATTTGAGGTCGTCTCTAGGGTGTACAGGTACTTGAGAACTTTAAAAAAACCTGATGCACAATCTGCAATGGCCAAGTTAAGCCAGCTGCTCCCCGAGCCTAGCGGCATCGAAATCAAATTCTTGCAAGATTTTGCCGACTTGGTTATCAATACCAAGACAGTACACACCATGATACAAGTAGATCACCTGACTCTTTTTTTTACCAAACGGTTAAAGAAATTACCGGCAATTGTTGCATCGAAATTTCGCTCGGTGCAGAGTCTGAGTCTCGATGATTTTGTGTCTATGCTAGAGCCTTATACTTTCCACGTGGAGCTTATTGATGTTAATTGGTCCTACAACAGCTTGGCTGACCAATTTACCGCCGAGGCTGAGCCAGAAGTAGACCTGGTTAACCTGATTGATGGGAAATTCGTCATGGGGCTCTTACCGGAATTTGCGGCTAGAACTTCTGCGCCATACACTCTATTAAAAAAAGGGCCATTTCCCTTTTATCGCCAGGGCTTAGAGATCAGTCATCGTGCGATGCAGTGCTTCGCCGTCAGTGTCCTTACAAATGAAGTTTACGACCCGGCTACCAGAAGAGTGAGTAGGGAGGAATTCATTAGTAGATTGTGCAGTAAGCTGGTGGCATCAAAATCCTGCCTATTCCCAAAGAGCAAAGTCAGAATGCTCATGGCTGATCTAAATTGGGTCAAGAGTTGCATACGCACAGCCAATAGTAGATTCGCAAGAAAGTTACGAGCGACAATTGCAGAGGGAAGGATCATGTGGCTCTTGAATAAAGACAGGTTTTACACCCCGGCCCTAGAGTGGCGTGCTGAAGGTTTGGGTACGTTTAAAGATTTGAGTGCCAGATGGGCCGGTGTCACCAGTGAGGTGATCTCACTACGGCCAAAATTGGCTAGAAGAGCAGTCCCACAAGAGTACGCTAGAGAGGTGGTCTACGAAAAGCCTGCACCTCAAGTAGGTGACGAGACAAAACCTAGCAAGGGAGAGCACACAGAGGAGCAAAGGGTGGAAGAGCAGGATCTCGGCAGTCTTGATTGTACTTGTGGCCTCAAAATTCCTTTAGGTAAAATGCTTGTTCCAGGAGAACATGGTTTCAAATGCCCTGATAGGCTGAAGGGAAGAAATGCTGGTTGGTACTCAAAGGATAACTGCAGTTATGAGTACAACGGGGGGCAACACTTGAGTTTGGGCCGGCCCCAGTGGATCGAGACATGGATGAGCTTGAATGATATAGACACTGATTATTACAATTGCTGTCTTTACCAAGTGTATGATGATAATTCCTCCATTGGTTTCCATCAAGATAATGAAGCCATATTTGAGATAGGGGGTAAAGTGTTCACCGGCAATCTGGTTGGGGAAGCATTGTTCAAGATCAAGTGTGCTTCTGGTTGCGGAGAAGTCGATCTAAGCGCTGGTGATTGTTTTACCATGCCTGTGGGATTTCAAGTTACCCACAAACATAGTGTGATGGGTACCACTCCTCAAAGAGCATCTGTGACATTCAGGCGTTTAGCGTCGGAAATAACCGGGGGGGGTGAGTCGACATCTAGCAGTGGAGCTACTGAGCCTGGGGAAGAATGTGTGTCTAACCTCTGCATTGGCTCTGTGCATTACAATGAATTACCTCCGGCGCTCAAGTACAGTACCGTGGAGGTGCCCGGCGATGGTTCCTGTTTCTGGCATGCTCTAGAGGTCCACACTGGGTTGAATGGACTGGCTATTAAAAAAATTTGCAAGGATGTAAATTTCCCTGATAAGGATTTGCAGAAGTCATTTGTGGCTCAATTAGGTGACGGAGTCTATGCCGAGGAATTGGCGATCATGGCCGCCTCTCTTGTTCTAGGGGCCGTTATTGCTATCCACAATCAAGAAACAAGATTACTGGCTAGGTTCATACCGAATGGGGAAGTAACGAGGGTGATAAACATTGAGCTAGAGCGGCAGCATTTTAGACCCATATTTCTGATAAATGGTTGTCTTGTCTCTGCGCTTGCGATTGGATTAGGACGCAGGGAATGTGACATAATCAAGGTCCTGGAAGATCAGACTGATCATGGCTTAGCTAGCATGTGGAGAGGCGAAGGAGTCGTGCTGGAAGAGTTAGAGTTCTACTTCAGAATTTTTGACATATGTGCTCATATAGATACAGAAGTGGGACCACGGGTTCTAAATGGTGACGGGAGGTTCCCAATGTGCTTTAAACTACGGGATCAGCACATTGAGTATATCAAGAAAGACCCGCAGGCCAAGATTGAACTGAACCTTGGGGAACAGATAGGACTGAGTGCCACTGAATCTTCACTCCTGTTCGTTGAGGGTGTCGGCAGTAAATTAACCTACATGGCAACAAAACAGCGTGCAGCAGTTCTGTCAGGGAGCCTTGAAAGCGGTGGAACAGGGGTAATTAGCTCAAAACTTTTCAATGATATGGATAACCTCATCTCAGATGAACATATGGAAAGTAGGGAAATTGAACTGGTTGGGGTTTTTGGGACTTTTGGTGCAGGGAAGAGTACAATTTTTAGAAGGTTCTTTGAGCTGAATGAGGGGAAGTGTGTTTTTTACATCTCCCCCAGAAAAGCGCTCGCCGAAGAATTCAAGGGGAAGCTTGACCTAAAGAGCAAAATGGGGAAATTAAAGTCCAAGTACTGGAGAGTGATGACTTTTGAATTATTCCTCAAGCAGATTCATCTAGCAAAGCCTGGCTCCGCCGTGATAATAGATGAGATTCAGTTGTACCCACCCGGGTATCTAGATTTAGTTGGTCTATTAATGAATGATAGGGTGAGAATCATAGTAGGTGGAGACCCTTGCCAGAGTGAGTATGACAATGAGAAAGATCGGGCCTGGCTAGGCATGATAAAGAGGGACCTCGATAGACTCTTAGAGGGTGCAACGTATAAGTACAACATACTGAGCCACAGATTCACCAACTCCAACTTTGTTGGTCGACTGCCTTGCGACTTTCCAACAGAGTTGCGGGCAGCTAAGGCCAAGGAACATTATCTGCTGAATGACCTCGAGGCACTGCGGGCACTAGAGGGGAGTTATTGCAAGACCTTCCTTGTTTCTTCGTTTGAGGAGAAGAAGATAATAGAGACTCACTTCTTTGATTTCCAACCACGAGTGTTAACTTTTGGAGAGTCAACTGGACTTAATTTTAAGAAAGGATCAATACTAATCACAAACATTGCCACATTCACCTCAGAGAATCGCTGGGTGACTGCGATTAGTAGATTCAGCAAAAACATCTGTTTTGTGAATCTCACCGGGACCACTTGGGATAATCTGCTTATGAGTTATAAAGGTCGGGTCCTTGCTAGGTTCCTCTCCAAAACAGCTTCTAAGGTTGACTTGCAGGAATTAATTCCGGGGAGCCCCTCTCTCACTGAGGGTTTTGGAAACATGGTCGGGAAGAATGAGGGGGTGAAGGAGGAGAAGGTGCAGGGTGATCCCTGGCTCAAAGCCATGCTGGACCTCTTCCAATTAGAAGATGTGGAAGAAGAAGAGGAGTTGATTGAAGAATGCGAAGAGGAATGGTTCAAAACTCACTTGCCTCAAGCTGAGTTGGAGGGGGTGCGCGCACGATGGGTTCACAGAATCCTAGCCAAGGAATTCCGAGAAGTGCGTATGGGCTATAATGTATCTGAGCAGTTCACCGATGATTACGCAAAAGAGGATGGAAAGGTGCTCACCAATGCTGCAGAGAGATTTGAGGCGATTTACCCCAGGCATAGAGCTAACGATACTGTAACTTTCTTTATGGCTGTAAAGAAAAGGCTTAGATTTTCCAAGCCATCCATTGAAAAAGCAAAGTTAATAGAGGCGCAAACGTATGGTAAATTTCTCTTAGGGGAATTTTTGAAAAGGGTACCGCTGAAAGGTAAACATGAACCTGCTCTAATGGCCAAAGCGAGGGCTGATTTCGAGGAGAAAAAAGTGAGTAAAAGTGCGGCAACAATTGAGAATCACGCTGGACGTTCCTGCAGGGACTGGCTAGTTGATATTGGCTTGATCTTTTCTAAGAGTCAATTGTGCACCAAATTCGATAATAGGTTCCGCGTAGCCAAAGCCGCCCAAAGTATTGTGTGCTTCCAACACGCCGTATTGTGTAGATTCGCTCCGTACATGAGATATATAGAGATGAAATTGCAGCAAGCGTTACCGAGCAACTACTACATACACTCCGGGAAGGGTCTTGAGGAATTGAATGCATGGGTGAAGAGGGGCAGGTTTGATGGTATATGCACGGAATCGGACTATGAAGCATTTGATGCCTCGCAAGATCAGTATATGGTAGCTTTTGAGGTCGAGGTGATGAAGTACTTGGGGCTTCCAGGTGATCTAATCGAAGATTACAAGTTTATCAAAACCCACCTCGGGTCCAAGCTCGGTAACTTTGCAATTATGAGATTTTCCGGTGAGGCCAGCACATTCCTGTTTAATACCATGGCGAACATGCTTTTCACCTTCCTGCGTTACGAGATCAAAGGTAATGAATATATTTGCTTCGCAGGTGATGATATGTGTGCTTCCCAGAGGTTGGCCACCAGGACCACTCACACTGGGTTCTTGAACAAGTTAAAGTTGAAGGCAAAAGTATTTATGGTGGACAAGCCCACATTCTGCGGTTGGCATTTGAGCCCAGATGGTATTTACAAGAAACCGCAACTTGTAATGGAGCGTATGTGCATAGCAAAGGAAAAGAACAATTTGGCCAACTGTATTGATAATTACGCTATAGAGGTTTCATTCGCTTACCGCTTGGGTGAACGTGCTCTAAATCGGATGGATGAAGAGGAGGCAGAGGCCTTTTACAACTGCGTGCGCATCATTGTTAAAAACAAACATTTGCTCAAGTCAGACATTGCATCCCTATATTCTAGAGCTTTGGAAACTTAGTAAGCTTAGGTTATTGCTTAATATATTGTATATGGATGTGTTAATTAAATTTGCGGTTAAATATAAGTTTGAAAGGTTGTATAGTAAATTAGATAGCCCCATTGTTTTCCACTGTGTACCTGGTGCTGGCAAGAGTAGTTGCATTAGAGAGATTCTCGCGTTTGACTCTAGATTTGCAGCATACACCCTAGGAGTTGAGGATCCTGCCAATCTCACAAACAACCGGATTTTATCCTACAAGGGTTCTGTTGATAGTACGAAGTTCAACTTGCTTGACGAGTACAACCTTGCCCCTGCAGAGAAGCGGGATTTCTTCGCAGTTTTCGGAGACCCCATTCAGGCAGTTCTGGAATACAGCTTACGTGCCCATTTCATCTGCAAATATAGCTTGAGGTTCGGCACCTGCACTTCACAATTCCTGCAGTCATTGGGTTACGAGGTTGAAGCTGAGGGCCCTGATGCTGTGCAATTAGGCGGTTTGTACGAAGTTGATCCGCGGGACAAGATAGTATATTACGAGGACGAAGTGGGCTGCTTACTGAGAAGACATTGCCTAGAAGCATTCGATATCTCCGAGGTCGTGGGTCAGACTTTTGACAGTGTGACATTTGTCACCAGCCATTCATCGCCACCTGTGCGAGATAGAGCTAAAGTATTCCAGTGCTTAACCAGACACCGACGAAACCTACTGGTGATGTGTCCTAATGGTTCTTACACCGCCACCTGATTACACAAGGGTCTTCTTGGCTTGTAGCATTGGACTGAGCACAGTTCTCTTAGTTTTTGTTTACTCTAGGAGCACCCTTCCCCTCGTTGGCGACAATTTGCATAGCTTACCACACGGCGGCTTTTACAAAGACGGCACAAAGACGATCCAATACGGCGCCCCTTGCAAGCTCAACTCCTTAGAGAATCATTACAGTTTACGAAATCAACCCTGGGCCTACGTTCTACTTCTGCTGCTGGCCATATTCCTGAGCGAAAGATTTTACAACAGAGGGGTCTGTTCCTGTGGTAGAGCACATGCACGATAACACAATTCTCACAATCATACTTGTAACGATAATTAGCTGTATTGCATTGCTAGCTAAGCCGCACAATCCGTGCTCCATTGCAATCACAGGGGAAGCAGTCAGAGTAGTAAATTGTGAGTTATCGCGAGATTTGTTAGAATTCATTCGGAGTGCCAAACCTGCAGGTTCTTGCTGACCCTTAGGTTTACGGGTGTTGTAGTTGATATACGTAAAGACAAGGGATATGCCGCCGAAAGAAGCGCCAGAATCTTCGAACCCTCCACCGGCTCCCCCGCCCCCACTGCTTGATGATGCGAATAGGAGACAGGAGCAGCCAAGGCCTGTGAGGAGTGAAGAAGATAGATCCATACAGGCCAGGCTAGATGCGTTGACAGAGATGCTTAGGGGCGAACGAAGCGCAATCAAGGTGACCAATGCAAGCTTTGAGACTGGACGACCGCCACTCCGGCCCACTGAAGACATGAGAGGTGATGTTACCAACATGTACAACAGGCCCTCCACAGATTTCCTTTGGAACTTGAAACCCAAGAAGGTTTCCAATAACATGGCCACCTCAGAGGATATGGTTAAAATCAAGGTTGCTCTGGAAGGGATGGGAGTGCCAACTGAGAGCGTGACGAACGTGATTATGCAGCTGTGTGCCTACTGCGCCAGCACCAGTAGCTCTGGATTTCAGAACCCGAAAGGGACTTTTGAGTGGCCGGGGGGCGCGATCATGGTGGATGACGTCATTGGTAAGGTTGTGGAAATTGCGGGGCTGAGGCGAGTTTGCAGACTGTATGCTCCAGTCACTTGGAACTACATGCATATTCATCAATGCCCACCTGCTGATTGGGCAGCCATGGGGTTCTCCGAGGAAACCAAATACGCAGCCTTTGATTGTTTCGACTATGTTCAAAATGGAGCAGCGATCCAGCCATTGGGGGGAGTGGTGCCAAAGCCCACACCGGCAGAACATGTGGCCTACCAGACATACAAACAGTTGGCCTTGGACAAAGCCGGTGCCGAACGCACATATGCGAACATGGATTCAGCTATTACTGGGGGGCGGCATGGGCCCGAGATTGTGCGTAACTACAATAATGCGAATAATAAACGTCAATGAAGAAGCTGGGGAGTAAGGGGCAGATAATATTACTATTGTGTGCTATGTTTGCTAGTAGGGGTAATGCTATACCCATACATATTATTGTTCATATTTACAAGCGTGGTTTTCCTAAATTGGTGGGAAAAGGCACCTCCACATATGCCAAGAAGCGTCGAGCGCTTTCCATAGGTCGTTGTGAGCGTTGCTACCGTGTTTACCCGCCTTTACCGTTTTCCAAGAAGTGCGATAACCGCACATGCGTGCCTGGTATTTTTAGTAATATTAAGGTGGTCAACTTCATCAAGTTTGGGAGTAGCCGAGGCGATACCCATCCTGGTTACAATTTTTAATGCTGCCATTAAAACTTAAGTAATGTATGAGTGTGTAGCTTTGTAAACAATAAAGTTTTAAGATATTTTTCCTTTTT

>JVC-Ph [organism= Jasmine virus C] isolate TZS, complete genome

GGATAAACATACTATACCTCATTATAACGCACGATATATTCCCATAACAAGGCATACACGAGCTTTAGAAAAATGGCTCTCACATACAGAACGCCAATGGAGGATATAGTATCAGCATTTGAACCAGCCGTACAGGCCGCCGTTGCTAGTACTGCAGGCAGTAGATACAAGCAAATGGAAGAGAACAACTTTGATCTCTTCAATTTTCACATGGATGCTGTCGCAAAGCAGAAACTATCAAAGGCTGGCATATATCTTAGTCCATACTCTGCCGTTGTACATTCCCACCCAGTTTGTAAAACTTTAGAGAATTATATTTTGTATAAAGTTCTACCTAGCTACTTATCAAGTAAATTTGTTTTTGTAGGTATTAAAAATAATAAGCTTTCTATTCTTAAAGCCCGGAACCCGAAGCTGGATTTGGTACAAGCTGTCAATCGGTATGTCACAAGCGCTGATAAAGCCAGATACGGCAACGAATTTGTTTACCGATCGAGCAAACCACATGAGGGGTTACTGCGGCATGCCCGTAATTTAAGTAGCGTCACACTGAAAGACTTGGTGCCGCCTTTAATGGTGCAGGGTTGTAAAGAAGTGTTTCTCCACGATGAACTGCACTATTATGGTGTGAATGATCTGTGCACCCTACTTGAGGTGCTCAAGCCAAATACTCTGCTCGGTACTGTGGTATATCCACCTGAACTGCTTGTGGGCGTCAAGACAAGCCTGAATAAGTGGTGCTATGATTTTGAAATTGTCGGGTCAGACTTGATGTTCTACCCTGATGGGGTTCGCGCAGAAGGTTACATGCAGCCACTCAGTGGCGGGTTCCTCCTGAAGAGTAATAGGATCGAACTGCAAAACGGCGATGTGTATTGCGTGGATATACTGTGTAGTAAATTCGCGCATCATTTGGTTGCAATCACAAGGGGTGATGACGTGGTACCTAAATATAGGGCCTTTTCCCCTTTTGACGCAGTTGGGCAAAAAGAGTTGCAACCAATCATGACCACTAAATCACCGTGTATACCCGTCAGCTTCGAATTGGTGAGCAGGATTTACCGGTACTTAAGAACATTGAAGAAGCCAGATAAACAGTCAGCAATGGCTAAGCTTAGCCAGATTTGTGTGGAACCGACGGGCTTTGAAATTAGCTTCGTCCAAGAATTTGCTGATTTAGTCATTGGCACCGGTACCATTAGAACGAACATTGATGCGGAACGTGTGAGAGGTTTTTTTGGCAGGCTAGGTCTGATGTTGCCAGGGGGTTTAGCAAGCCTTTTTGGGGTTTCAAAGGAAGTGGCACTTGAGTCGTTCATCGAGAGATTGACACCATACACAATTGGTGTCAAGCTCAAGACTGTAACATGGGGAACTGATTTGCTTGAAATGCTGCTTCCTGACGTGGATGTAGAACCCATGTTTGATGTGATTGAGGAATATGGCGTACAATTCACGCAGGGAAGGGTTGCTGGCATCTATGATCGAGTGCCGCAACCGTACGTTGGAGTTTGCGGTAGTGAAGCGGAACGCGGATGCACTTACACTTTCTTTGGCGTGGAACAGGTTCTACAGTTTTTGCCTGCACTCGTTGTAAAGATTCAAGGTGTTGAAAATTGCAGAAATTGCGACTCTGCAAAAGTGGAGGTGGCCGTCAGAGAACTTGAAAGGAAAGGCACGGTATTGGGCTTCAAGGTTCTACAGGTTGTTGGTGAAGTTTCGATGATATGCTCGGGGGTAGCTGCCAGAGTTCAGAGGCTCGTAAGGAGAGGTCGTTCGACATTGATCTTCGACCTTGGGTTGAAGTGGTTCTTCACTGGCGATGCACAGGATACGCTCTTCATTGAGAATGTAGCTAATGACATACCCCGATTGAGTGGGTACTCACAGTGTTGGAGGAATGTTGTTCAAGACGTGCTGCAGGGGGGGTCGCGTCTTCGGAAAACCCGTAACACGAGGGCTTATACGCACCTGATTCGAGACCAGATTGGTAGTAAGACCACATGGAGCCCAGTTAATCGTAGTTCGCAAAAAACTTGTGTCTCAGAGGTTGCGAATGATAGGGGCAGTTCCACAGGTTGTGACATGTGTGACGCTTGCATGCAGATTAGCCCCCTAAAATATGCCGAACATCTATTCACGGCGCGAGATTCTTTGAAAAACCGAAAAGCTACATGGTACTCCAAAAATTGTTCCGAGTACAAGTACAATGGGGGAAGGCACAAGAGTGTTGGTTGGCCTGAGGAGATGGTACTCTGGATGCAGGCGAATGAGATACCCGCAAAATATGATTGCTGCCTGGTGCAGAAGTATGCAGAAGGCGCTAAGCTAGGCTTGCACGCTGATGATGAAGATATTTTTGAGCCAGGAGAGAGTATATTGACCGTTAATTTGTCTGGAAAGGCGACTTTTCTCATCAAGTGTTGTGAGGAGCTTCGGAAAATTCACTGCAATGGGCCAGGGATGTTCACAATGCCGGAAGGTTTCCAGTTTCGACATAAACATGGAGTCCAGGACACAGCTGGAGATCGATGGAGTGCCACCTTCAGATGCCTGAGAAGAACTATACCTAATACCCATAAACTTGAATCAGGGATAGTGGAGCCTGTGATTCTGCCTAAGGAGGAGTCGGGCGTCGTTGGGGCGTTAGAGGGTTTCGATGTGAATGTTGCGGGTTGTAATCGGCAGGTGGTTGATTGGAAGTTTGGCGCTTCCTTTGGAGTGGTAGAGACCCCAGGAGATGGATCTTGTTTCTGGCATGCAATGTCACACTTCCTAGAAATCTCCAGCACGCAAATCAAGACTGTAAGTATGAAATGCCAGGATCTGCCATCACCTCTACAGAAAGATTTGGCAGTTGAGGCAAAAGAGGGTGAATTTGCGTCTGACGCAAGCATTTACGCTACGGCCATAATGCATGGGGTGCGGATTTCAGTGCTTGATGCCAGGGATCAAAAATTGCATGTCTTTGAGCCCAGCAAGATGCGGACTGATGCATTCTTGTACTTGAGAGGATTCCACTTTGAACCCATCGTACTCAAGAATGGTTGTGTGGTTAAAGCCATTGCAAGCTTACTAGAAAGACGTGAGCAGGATGTGCTGGCTGTAGTTGAGCGGAGAGCAGCTGTAGGCATAAGGGAAGAAGTTTGGAGGGGCCTAGGTTTGTGCCTCAGCACGATCCAGGTGCTGTTGGAGTTGTTTGACATACGCGGCGTGATAAAGACTGATGACAGTGAAATTGTCATGAATGCGAAAAGTCGTATTACGGGTTTTTTTGAATTGAGAGATGATCACATGACCCATATCAAACGAAAGAAAGATGCTGCAACTAGTGATTTGCAAATTGCAAAGAATTACAAGTTGTTTTCTCCAGAAGAGCTGGTAGAATGTTTCGCCATGGGGACAAAAGTTGGATACATGATTAGGCTACCCGCGGCCACCATTCTTGCGAACAGCTTCAATAGCGGTAGCACAGGTGTGCTTCTATCAGACATCTTCAATGATCGACCCAATTTCAAATTGGAGTTTGTGCAGGCCAATGGGACCATCGTGGATATAAATGTAGTCTGTGGTACTTTCGGCTCAGGCAAGAGTTATGCCTTCAAACAAGTTCTCAAGAAGGCTAGTGGGAAGATAGTGGACTATGTGGCTCCGCGAAGGGTTCTGAAGGATGCAATGTCCAAGGAGGTGGGGCACGAGAAGAAGGCGAGGAAGGCAGGTCAAGAAAATTGGAATTTCACAACTTTTGAAAAATTCCTAGATCGATGCAAATACTTGGTGCCAGGCCAGCTCGTTTGTTTTGACGAGTTCCAGCTGTACCCCCCGGGCTATTTTGATTTAGCTTTTGCTCTAGCGCCTATTGGAGTTAATTATCTTCTTTTGGGAGACCCATGCCAGAGCGATTATGATTGTGAGCGGGATCGCAGCAATTTCATCGGAGCCACAAGGAATGTCGATCGCACGCTCGATGGTCAGACCTACAAGTACATTACTAAAAGCAAGCGATTTGTAAATGCGACTTTCTGTGGGAGGTTACCTGCAGTTGTGGATAGTGATGTGCTTTGCACGGAGGAACCTTATTCCATACGCGAGGGGGTAGAAACAATCCCAGAGGTCTTATCTCAATTTGGTGAGGTGGTACTCGTTAGTTCTTTTGATGAGAAGAAAGTTGTTCAGAGTTATGCACCTGAGGCCAAAACCCTCACTTTTGGTGAGAGCACAGGTATGACCTTTGAACGTGGCTCTATTCTTATTACATCTGTTAGCGAGAGAGCCAGTGAGGTGAGATGGGTAACTGCACTAAGTAGGTTCAGGCGCAACTTATGCTTTATCGTCTGTGCACCCTGTCACTTCGAAGGGTTGAAAATTTCCTATCGTAATAGGTTTTTGTGGAAGTTTTTAACACAGAGCGCCTCTGTGGATGATCTAAAACCCTACTTACCTGGGAACCCTGTGTTCTTGGAAGAGTACATGAGTCGCATCGGGAAGGACGAAGGCGTTCGGGAAGAGAAATTGCTGGGAGACCCTTGGCTCAAGGGGATGGTGGACTTAATGCAGCAAGAGGATGTTGAAGAGATCGTGGTACTTAATGAGGTGTGTGAGGAGGAATGGTTTAAAACGCATCTCCCTAGGGCAGAGCTTGAGAGTGTTCGTGCCCGGTGGGCTCACAGATTCCTTCTCAAAGAATTGCGGGAGCATCGCATTGGTTGCCTCACGTCGGAACAGTTTACTGACGAGTACAGCAAACAGATTGGAGGGGTCCAACTCAGTAACGCAGCAGAAAGATTTGAAGCTATTTATCCAAGGCACAGGGCCTCAGACACTGTCACTTTTCTCATGGCTGTAAAGAAAAGATTGCGGTTTTCTAAACCTGCGGTCGAATGCGCCAAGTTGGCTAATGCTAGGAATTATGGAAAATTTTTGTTGTCGAAATTCCTCGAGAAGGTTCCACTGAAAAGGGCCCATGACCAGATGGCTTTCGAATGCGCCAGGCAGGAATTCTTCACGAAGAAAGTGTCTAAGAGTGCTGCAACGATTGAAAATCACTCAGGCAGGTCTTGCCGTGATTGGCTTGTTGATGTTGGTCTGATCTTCTCAAAGAGCCAACTCTGTACTAAATTTGACAACCGCTTCAGGGTAGCGAAAGCAGCGCAGAGTATAGTTTGTTTCCAGCATGAGGTGCTCTGCAGATTCGCCCCGTACATGCGATACATAGAGAAGAAGCTGCATGAGGCGCTACCAGACAAGTTCTACATCCATTCCGGGAAAGGACTTGAGGAGTTGGATGAGTGGGTTAAGAAAGGCAATTTTGGAGGGCTTTGCACGGAATCTGACTATGAAGCTTTTGACTCTTCCCAGGACCAGTACATCGTTGCCTTTGAAATTGAATTGATGAATTATCTAGGACTCCCGAGAGACCTGATCAATGATTACATCTACATCAAAACTCATCTCGGGTCGAAGTTAGGCAACTTTGCGATTATGCGGTTTTCGGGTGAAGCGAGCACCTTCTTGTTTAACACTATGGCGAATATGCTCTTTACCTTCTTGAGATACGAGCTTAGAGGGAATGAGTACATATGTTTCGCTGGGGATGATATGTGTGCTTCCAAGAGATTAACTATCAAGCGTGAGCATGAACAATTCCTCGGAAGATTGCGCTTAAAGGCTAAAGTTCAGTTCACAGAAAAACCGACTTTCTGCGGCTGGCATCTTTGTCCAGATGGGATTTATAAAAAGCCTCAGCTAGTCATGGAAAGACTCTGCATTGCTCGTGAGAATAACAACCTGGGTGATTGCATCGATAACTACGCTATAGAGGTTGCTTTTGCTTATAGAATGGGGGAAAGGGCTGTGAATCGCATGGATGAAGAGGAGCTTGATGCCTTTTACAATTGCGTCAGATTGATTATAAGGAATAAACACCTGCTTAAGTCTAGCATTCGTGAAACCTTTGAGCGTGGTGAGCAGTAGAGGCTTAGGTTTCAACCATTGTATTGATAGATGGAATTGCTGTTGAATAAATTGGCGTCTTTTGGCTTTACTAGGATAGGTAGTTCTTTGCGTTTGCCCATAGTTGTCCATTGTGTGCCCGGCGCAGGAAAAAGTTCGCTGATTCGTGCGATACTAGAGGAGTCCACAGAACTGAGAGCTTACACCTTCGGACAGGCGGATCAACCAAACCTGATTGGCAACTACATTCGTCCTTTCACCAGTGACTCGATATTGGATCAAAGAACCATCATCGACGAGTACACATTGAGCCCACAACCAATTCAGAGCGTCCTCGCTCTATTTGGTGATCCGTGCCAGCCAGGGGTGGACCAGGGACTTGTCGCAAATTTCCTCGGAAACTTCTCTAGAAGATTTGGCTCAAACACCGCCGCCTTACTCCAAAAGTTAGGGTTCAACGTACGTGCAGAGGGAGAGGACACGGTCCAAGTGCTGGATATTTTCAAGGCCGAGCCCACTGGAGTGATCATTTGCTTTGAGCAGGAGGTCCAGAAACTTCTTTGTGCCCACAACCTTGAGTACCACACCATTCAGGAGATCCAAGGTTCAACCTTCCAGGAGGTCAGCTTCATCGTGTCAGGACCGTTCCAGATTGAAAGGGCGCGTGAACACTTTCTTTGCCTCACAAGGCATCGGAGGACCTTGAATATACTGTGTCCAAATGCCACTTACACCACCTCCTGACTACACCAAGGCAATCATCTGTGCGGTTGTGGGGATTTCACTTGCACTTGCCTTAGGCCTTTTCACTCGCTCGACTATACCATTCGCAGGTGACCAGCTGCACAGCTTGCCACACGGGGGTTGTTACCAAGACGGGACCAAGAAGATTTTTTACAACCGCCCAAGAAAGCTCAATTCCATTGAGCAAGCCGTGGTGAGTCGTGAGATTGTCTTCATTGTCATAATCTCTCTAGTTGGCCTCCTCCTTGCGACGAGCTTCAGAAGGCGAGCAATTTGTGGTGCATGCGGACATGCGCGGCATTGATTTATTTGCGATTGGGCTAATTTGCTTCGCTTTAACCTTGTATATACTGAGTGTTTTTAATTCTGGTACTTGTGTGGTTGTTATCACTGGTGAGTCCATTAGAGTGCATAATTGTCCAGTCAGCCCTGAGTTGTTTCACAGCATAGCTGCCACGAAACCGTTCAGGGGTTAGAGTACTTTTAGGTCATTAAGTATTAGTTGATATACTTCAAGATGAGTTCCACTTCAGATGACAATTCCAAAGGCAAGAAGCCTGTGGTGATCCCTGAGACCCAATCCGGCCAGCCACCGCCAGAGCAACCTGCTCGGGAGGGTACAGACTCCCATAATTATGAGTTGGATGCCCAAGAGGAGCAACTTGAAGCGCGGATGCAGAAACTAAAACAGTTTCTTAAGCAACAACAACGGGCCACTCAGGTGACCAATCCAAGCTTCGAGCTCGGGCGGCCGAAACTCAAAATGCTCGACGGTGTTCGGAGTGACCCAACAAATCTCTACAACAAGCCGACGATCGATCAGCTGTGCTCGATAAAGCCCAGGGCCATATCCAACAACATGGCCACTTCTCAGGACATGGCAGCGATAACTGTAGCAATCGAGGCTCTGGGAGTTCCCTCGGAACACATTCAGACGGTGCTAATACAGGCAGTGGCCTACTGCAAAGATGCTAGCAGTTCCACATATTTAGACCCTCAAGGGGTCTTCGAGTGGGGAAGTGGTGCTGTGATGGCTGATTCCATTCTGGCCATACTGAAGCGAGATGCGGGCACGCTAAGGAGGGTGTGTAGGTTGTACGCACCTGTGACTTGGAACCATATGCTTGCACACAATTCACCACCGTCGGACTGGGCTGCAATGGGCTTCCAATACACAGAACGCTTCGCAGCGTTCGACTGTTTTGATTATGTCGAAAACTCAGCTGCCGTGCAACCATTCGAGGGATTGATCCGAAGACCAACCCCAGCTGAAAAGATCGCTCACAACACCCACAAGAGACTGGCACTAGATCGCGCAAACCGAAATGAAAAATTCGCCAATCTCGAAGCAGAGGTCACTGGGGGCCACATCGGGCCAGAAATTGAACGAGGAATTTGGAAGAGATGAGGTACGAACTTGAAGTTGCACTAGTAATTAATAGGGTATTTCAGGCAAGAGGTGTGCATAACCTTGCCTTAGCACTTTATATTTCCAAAAAGGCCGTAGGGCCATGCTTGAATTATGGGAGATCAACCTACGCCCGTAGGCGTAGAGCGAGGTCCATCTCGCGGTGCTACCGTTGTTACCGAGTGTACCCACCCTTGAGTGGAAACACAAGGTGTGACGGGAGAACTTGTTTCCCCGGTATCAATTATAGGGTTGACGTCGAGGAATATATAAAATTTGGAGTAGCTGCGGCGATACCAAATTTCGAGCTCTAATTGCGACTAAAGCCTAAATAATAGATACCGTCGTAATTTTAAAAATAATGTGTTTTTAACTATTTTTTCC

>StCV1-Ph [organism= Stevia carlavirus 1] isolate TZS, complete genome

GGATAAACAAACATACCTTCCTCTGATATATAGCAACTAAACATCCGTTCAGCTACTATACATTGCAATGGCCCTCACATACAGGAGTCCATTAGAGGAAAACTTTGCGTCTTATGATTCCAGCGTCCAAGCAGCTATTGCTAGTACCTCTGCATCATTCTACAAAGATTTGGAGCAAGAGAATTTCCGTTTCTTCAATTATTACGTGCGCCCTGAGTGCAAGAAACACCTAATTGATTCGGGAATTTATGTCAGTCCTAATGCAGCTGTGCCACACTCGCATGCCACCTGCAAGACACTGGAAAATCATTTTTTGTATATAGTACTGCCACCACTCGTAGACAATTCCTTTTTCTTTGTCGGAATTAAAGATTCGAAAATAGGTCTACTTAAATCAAGAAATAGTCAGTTAACTATGGTCAATAAGTTAAATAGGTATGTCACTAGTTTAGATAGGATCCGGTATGGCCCTGATTTCGTTGTGCGAGCGACCAAACCCATACTCGGCATGAAGCGGCACCAGCCAGTTTTGGAAGATTGCACTTTGAAAGATCTGGTGCCACCATTGATGGAGCGATCAGCGAAAAGATTATTCCTGCATGACGAGTTACACTACTGGAGTCATAGGGATTTGATCACTTTTTTAGAAGTTCTCAAACCAGAAGTGTTGCTAGCCACATTTGTATTCCCTCCTGAGATCTTATCCGGTTCCGAGCAGAGTTTGAATAAGTGGTGCTACACCTTTGAGATGATTAAAGGAGACCTGATCTTTTACCCAGATGGGGTGCGCACGGAAGGGTATCAGCAACCACTGAGCAGCGGTTATCTGTTGAAGACCAGACGTCTCATACTCAACAATGGAGATTTTTACAATGTGGATGTCGTGCAGAGTAAGTTTGCTCATCATTTGATTAGCATAACTAAAGGCAGATACGCTGGCCCAACAATTAGAGCATTCGGCCCATTCCAAGCCACCTCTTGTAAAGGGCTTGAGCCGCTTACCCGTAACGTCACGAATTGCTTCCCGATTTCATTTGAGGTTGTGTCCCGAATTTATCGGTACTTAAGGACCTTAAAGAAACCTGATCCACAATCAGCCATGGCAAAATTGAGTCAGCTACTTCCGGAGCCTGATGGTATCGAAATTAAGTTCCTCCAAGAATTTGCAGATCTGGTCATAAATACGAAAACTGTGCACACGATGATTCAAGTAGATCATCTGACTCTATTTATGAGCCGCTGGCTGAAGAAAATGCCCAGCGTCATAGCTTCTAAGATCCGACTTGTGCAGAGTGTGAGTCTGGATGACTTCATCTCCATGCTAGAGCCTTTCACGTTTCACGTTGAGCTGCTTGATGTTGATTGGTCACACGGGAGTTTGATTGGGAAATTCGAGGCGGAGGCGGAGCCAGATATTGATCTGATAGGGCTGATGGACGGTAAATTTTCCATGGGGATGCTCCCCGAATTCGCGCCCAGGGTGCCGGCCCCAATTCGATTGGTTAAGAAGTACCCATGGCCGGACATGAGGGTTGCGCTTGCTATTGGCAGGGAAACCCTGAGGAAATTCTTCATTACTTGTATTGGGAGGGAGTTTTTACACCCCACAACGCGAGAGTTCAATTGTACTGTAATGAGGGTTTTTATCGAAGCAAAGATTGTGGCTTCCAAAAACGTGGTACTGCCAAAAGGGAGCATGCTTAGCTTAATTCGAGAAAGGGATTGGGTCAAAGGATGTGTCAGCACGATTCGAAGTGTGTATGGGAGGAGATTCAAGCTGACATGCTCGGAGGATCGAATTTGCTGGCTTCTTAGGACCGAAAGGTTCCATGAACCTAGGCTCACGAATGTAAGTCACCATCATGGCACTTATCGGAGCACAGCAAATGAGTGGGCCAAAGTCGTTGCAGAGCTGAGCCGAGGTAGACCACTCCTGCAGAGAAGAACACCCCCAATCCAATATTTGCCACGCTCTGAAGTGGTGGTGCATGAGGAAGTGGTCCCAGAAGAAGACGCAGGGGTTACACCAGAGCAATCCGGTGAAGTGGGGGCGCACTCGGGGTGTAAATCGCTGAACTGCTCATGCGGCGTGCAAATGGATGTTGTGCGAATGATTGTTACCGGTGATCACTGTTTCAAGAGTCCAGACCGCTTGAAGAATAGGAACGTTGGGTGGTACTCAAAGCATGGACTCGATTATTTCTACACCGGGGGAACGCACAAGAATTTGGGGTGGCCCGCTTGGTTAGAACTCTGGATGGAGCTGAACTCAATTGACACGACATATTACAATAGTTGTCTGTTCCAGGTTTATGACAAGGCCGGTGCGATACCCTTCCATAGTGACGATGAGGACATTTTTGAGCCTAACTCTAAAATTTACACGGGCAACCTCAAGGGCGAAGCGGATTTCAAGGTTAAATGCAAAAAAGGTTGTGGCAATGTACACCTAACCCCAGGTGTAGGGTTCCAAATGCCGGAAGGTTTCCAAGAATCGCACAAGCACAGTGTCACGGACACAAGCCTAGGGAGGGAATCCGTTACCTTCCGTCGAACCACAAGGCAGGAGCCCGAGGTAGTTGATACCATGGAGTCAAGTGATGCTGACACGGAAGAAGACAATATTAAACTTGACTTGCATATTGGGTCAGTATCATACAAAACCCTAGAGCAGGATTGGAGGTACATTGCGAAGGAAGTCGCGGGCGATGGTTCTTGCTTTTGGCACTCATTGGAGGTGCATACAGGATTAGCAGCTATGGAAATAAAGCGTTTGTGCGCCAATGTGGAATTCCCTGTGCCAGAACTCCAAGGACACCTGATCAGACAGATGGGCGAGGGTGTGTACGCCGAGGAGGTGGCGATCATGGCCGCGGCCATGGTCCTGGGTGCCGTAATCGTCATTTGCAACAAGGAGAAGGGTCAAAGAGCTACATTCCTCCCGCACCGCACCACCGATAGAAAAATCCACCTGGAACTTGAGGCGGCCCACTATCGCCCTATTTTCCCTGCCAACGGCTGTTTAATCACTGCCATTGCGATGGGCCTACAGAGGAGGGAGTGTGATGTACTTAAGGTGATTGATGAACAGGTCGGCGACAGTAAAATCTGGCTGGGAAGTGGCGTTGCTCCAGGAGATCTGGAGTTCTTCTTCAAAATCTTTGACATATGCGCTCACATCGACACAGACTGTGGATCCAGGGTGCTCAACACCCAAGGTCGCTTCCCATTGAGTTTCAAATTACGGGAGGATCACATCACTTTCGTTGGCCGGAATACACAGGTCAGCATTGAGATAAATAGAGGTGAACAATATGGGCTGTGCATCACCGATCAGTCAATGATATTCATTGAGAAGGCAGGTAGTCAGCTTAAATACCATGCCACGAGAGAGAGGGCATCAGTTCTGGCCAAAAGCTTTGAGAGTGGAAGCACAGGGGTCCTCAATTCAAAATTGTTTAACGGAGCTGCTTGTCTGATTACTGCAGATAGTCCACTTGAACGGCACCTGGTGGTAACCGGGTTATTTGGGACATTTGGTGCAGGAAAGAGTACAGTGTTCAAAGGTTTCTTCGAATTGAACGAGGGGAAGGGTGTTTTTTACGTGTCCCCGCGGAGAGCCTTGGCAGACGAGTTTCGAGGGAAGGTTCAGCTTGATGGAAAAATTGGTAAATTGCGGTCGAAACACTGGAAGGTAATGACTCTTGAGATCTTCCTCAAGCGATATCATTTGGTCAAACCGGGCATGGCACTTATTATCGATGAGATTCGATTGTACCCGCCCGGTTATCTAGACCTACTGGGGTTGCTGATACCTGAAGGGGTGCACATCATCGTCGGTGGTGATCCTTGTCAAAGCGAGTATGATAGTGAGAAGGACCGGGCTTGGTTATCAATGTTGGAGAGGGATGTGGACCGGCTTTTGGCCAATCAGGATTACTATTATAACGTGTTGAGCCGGAGGTTTCAAAACCCTAATTTCTCTGGTAGGCTACCATGTGAACTGCCGATTGAAGTGCGGCGTGGCCAGATGCTTGAGCATTATCTAGCCTGTAGTTTGGAAGAGGTGAAGGCCATTGGCAGTAGCTATTGCCGCACATTTTTGGTTTCCTCCTTTGAGGAGAAGAAGATCGTGGAGTCGCATTTCTTCGATCAGCAGCCAAAGGTCTTCACGTTTGGGGAATCTACAGGGCTGAATCTCAAAAAGGGAACAATTCTTATCACGAACATCGCGCATCTGACTTCAGAAAGGAGATGGATAACTGCCTTGAGTCGATTCAGCAAAAATGTGTGCCTAGCGAATTTGACAGGTACCCCTTGGAGTAATTTGGTCACAGCTTACAAGGATCGGGTTCTTGCCCGTTTCCTCACGAGGACGGCCTCCATCAAAGATTTACACAGTTGGATACCTGGGAGGCCCGACTTCAGAGAGGGATTCGGAGACAATGTCGGGCGCAATGAGGGGGTTAAAGAGGAGAAGTTGGCAGGTGATCCATGGTTGAAGGGCATGCTAGATCTATTCCAGTTGGAAGATGTCGAGGAGGAGGAAGAGCTAATCGAGGAATGTCAAGAGGAGTGGTTCAAGACTCACTTGCCCCAAGCAGAGCTGGAGGGTGTGCGAGCGAGATGGGTGCACAAGATATTGGCCAAGGAGTTTAGAGAAGTTAGAATGGGGTACAATGTTTCTGAGCAATTCACCCATGAATACGCCAAAGAAAATGGCAAGATTCTCACCAATGCGGCAGAGCGCTTCGAGGCCATCTACCCGAGGCACAGGGCGAATGACACCGTTACCTTCTTCATGGCAGTAAAGAAGAGGCTTAGATTCTCCAAGCCTTCCACAGAGAAGGCCAAATTGATCGAGGCGCAGATGTACGGGAAATTTCTACTGAATGAGTTCCTCAAACGGATTCCTCTAAAGGGTAAACATGAACCCCATCTTATGAGAAAGGCGAAGGCTGATTTCGAAGAGAAGAAGGTGAGCAAGAGTGCGGCAACTATAGAGAATCATTCTGGCCGTTCCTGCAGGGACTGGTTGATCGACATTGGGTTGATATTCTCGAAGAGTCAATTGTGTACCAAGTTTGATAACAGATTCAGGGTGGCTAAAGCAGCACAGAGTATAGTCTGCTTCCAGCATGCAGTACTCTGTAGGTTTGCGCCATACATGAGGTACATTGAGATGAAATTGCAGCAGGCCCTGCCCAGTAATTACTACATCCATTCGGGAATGGGACTTGAAGAGCTGAATGCTTGGGTGAAGAAAGGGGGTTTCTCCGGAATATGCACTGAATCTGACTATGAGGCCTTCGATGCTTCTCAGGACCAGTACATGGTAGCGTTTGAAGTTGAAATCATGCGTTTCTTAGGCCTCCCCATGGACTTGATTGAGGACTACAAGTTCATCAAGACACACCTGGGCTCAAAGCTTGGTAACTTCGCCATCATGCGGTTCTCCGGGGAGGCAAGTACCTTTCTGTTCAACACAATGGCTAACATGCTTTTCACATTCTTGCGCTACGAGATCAAGGGATCTGAGTACATCTGCTTCGCAGGGGATGATATGTGCGCATCAGAGAGGTTGGCCACAAAAAAAAACCATGAGGGTTTCCTTGCGAAACTCAAATTGAAGGCCAAGGTGTTCATGGTGGATAAACCCACTTTTTGCGGTTGGCATTTGTGCCCAGATGGAATTTACAAGAAGCCGCAATTGGTCATGGAACGGATGTGCATAGCCAAGGAGAAGAATAATCTCGCTAATTGTATTGATAATTATGCCATCGAGGTGTCCTTTGCCTACAGATTGGGTGAGCGCGCAGTTAACCGGATGGATGAAGAGGAGGTGGAACCGTTTTACAATTGCGTCCGTATAATTGTTAAGAACAAGCATTTGCTCAAATCAGATATAGCTAAACTCTTCTCCAGGGCCCTAAATGATTGATAGCTTAGGTTTGTGCTTTTAGGATCGTTTATGGATGTGTTGATTAAATTTGCGAGCAAGTATAATTTTGAGCGTTTGTATAGTAAATTAGATAGGCCTATAGTTTTCCATTGTGTGCCGGGTGCGGGCAAGAGTAGTTGTATTAGGGAAATCCTTGCATTTGATTCAAGATTCGCTGCATTCACCTTGGGGATTGAGGATCCCCCAAGCTTAACAAACAACAGGATCAAGAAGTACTCCGGTGAGCTGTCAGACGACTTTCTCAATATTTTGGACGAGTACACGTTGGAAGGGGTAGATACAAGAGGATTCATAGCAGTCTTTGGAGACCCTATTCAATCAGCGGAGGATTACAGCTTAAGGGCGCACTTCGTTTGCCGCACTAGCTTGAGATTTGGGAAGTGCACCGCACAGCTACTGTGTGAACTGGGCTACGAGGTTAAGGCTTCTGGGGAAGACGTCATTCAGATTGCAGGGTTGTATGAGATTGACCCCAAGGACAACATCATTTTCTACGAGGAGGAAGTCGGCTGCTTACTGAGGAGGCACTGTTTGGAAGCGTATCATATCAAAGAACTTGTTGGACCAACTTTCGACCGTGTTACTTTCGTGACAAGCCATTCAAAGATACCAAGGGAAGCCCGTGCAACTGTTTTCCAGTGCCTCACTCGACACAGAAGAAGTCTATTGATCATGTGCCCGAATGGCTCTTACACCTCCTCCTGATTATACAAGATCCGTGCTTTGTTGTGCCATCGGTTTGAGTTTGGTCCTTTTAGTTCTTGTGTATACGCGTAACACTTTGCCTCCAGTCGGGGATAATCTGCATAGTCTACCTCACGGGGGGTCTTACCGGGACGGAACAAAAACAATCCTCTACAACTCCCCGAGTAAGCTGAACTCACTCGAGAAAGGATACGGGTTGGCTAATCAGCCGTGGGCCTACGTAATACTGATCACTGTCGCGATCGTCGTAAGCGAAGTATTCTACAGGCGGAGGATTTGCTCGTGTGGGCGTGCACATGCATGAAAATTCGATCCTTTTCGCTGTGCTGATTATTATTGTTGGCTGTATCATCACCCTCTTGCAACAGAACAAGCAGTGCTACATTGTAATTTCAGGGGAGGCCGTAAGGGTTATTGGTTGTGAGATAACAGAGGAGTTAGTGAGATTCGCAAAGGAAGTGAAACCTGCGGGCTCCTGTTGACCCTTAGGTAATCGGGCTGTAATATCGATATAAATAATTCAGATAGATATGCCGCCCAAGGAAGGACCATCAGTGCTCCCAGAGCAGCAAGCAGCTGCGCCACCCCCAAACAATGAGCAGAGGGCAGAGCGGCCCCAGGTTCGAAGTGGAAATGCGGATGATCGAATAATTTTGGAACGCCTCCAAGCACTCACTGATTTGCTGAGGCGGGAACGTAGCGCAGTGCCCGTTACCAATGCAAGTTTCGAGACTGGGCGCCCCCCATTACGGCCTACTGAGGATATGAGGGGGGATGTCACCAATATGTACAACTTACCCTCAACTGACTTGCGGTGGAGCATCAAGCCAAAGAAAGTGTCCAATAACATGGCGTCCTCAGAGGAAATGGTGAGGATTAAGGTTGCGCTTGAGGGACTTGGAGTCCCCACGGAGGAGGTCACCAGCATCATCATACAGATGTGCGTGTACTGTGCTAACACGAGTAGCTCTGAGTTCCAGGACCCGCAGGGAACTTTCGAGTGGAAGGGGGGAGCTATAATGATTGATGACGTTATCGGCACCGTGGCCAAATTAACCAAACTGCGTAGAGTGTGCCGTCTGTATGCACCAGAGACCTGGAATTACATGCATATTCACCGTAGTCCTCCGGCAGACTGGGCAGCCTTGGGGTTTAAGGATGAAACGAAATATGCAGCCTTCGACTGCTTTGATTATGTGCAAAACCCAGCAGCTGTGCAACCACTTGGGGGAGTTACGCCGAAACCAACGCCCGCGGAGCACGTGGCTTACCAGACTTACAAACAAATGGCTCTAGACAAGGCCGGGGCGGAACGGACGTACGCTAATGTGGATGCCTCCATAACTGGCGGGCGACATGGACCTGAGACTATTCGTAATCATAACAATGCGAACAACAAACGACAATGAAGGAGTTGAGCGACAAAGGCAAGGTAATTTTAATATTGTGTAATGTGTTTGCTGAGCGCGGACCTGCAGTCCCGCTCCCTATCGTAGTTAATATCTATAAGCGTGCTGGTTTCCTTAAGGTAGTTGGTAATGGGACTTCGACGTATGCACGACGCAGAAGAGCGTACTCTATCGGACGCTGTGAGCGATGCTACCGAGTGTACCCACCTTTGTGGTTTTCCAAGAAGTGCGATAACCGCACATGTGTGCCTGGTATTTCAAGTAACATGAAAGTTGTGAATTACATCAAGTATGGGAGTAGCCGAGGCGATACCCACTCTGGTTGTAACTTYTAAAGCTGCCATTAAAGCTTAATTAATGTATGAGTGTGTAGCTAATAATAAACAAATAAGTTTTAATAGATTTTTCCT

>RNA1-ZRGX1 [organism= Broad bean wilt virus 2] [isolate= ZR-GX1] segment RNA1, complete genome

GTTTTAATAAATTATTGAAACAAACAGCTTTCGTTCACTAAAACAGCTTTCGGTTACTAAACTGCTTTCTTGTCTTTGAATTCTCAACAGCTTTCGGTTACTTTAAACAGCTTTCAGTTACAAAACAGCTTTCTTGCTTTTTATTTCAATCTTTCTTGTTTTCTTATTTTAGAGATGGATTTCGGTATGATGCAAATGGTTGTGGGCTTTTTGAAAACTTCAATGGGTTTGCAATCCATTAAGGATTTAGTTAGAAAGGCCAAAATAGATGAAAGGGAGAAGGAGCTTCTTCATATTCATCTTTGCTTTTTCCATGCTAATCAAATGGCAATTGATTATAATGAGGGCATGGACATGGAGCAGATGTACAGTCCTATGGCCATAAAATATCGGGCTGCTATCCTTGCACGACATGTGCAATTGATGGTGGAAACTGGTAGGTATGATAGGAAACGCATGTTGGATTACAACAACGAGACATGCGTTAACTGGTTTTCGTGTGGCTTCGTGGCAAGTGAGAATGCCGCGACCACAATGGACGAAAACCAGGAAGAGGGAATTGTCAATGAATTTCTCCGGGGCCGCACCATTGGTGGTGAAAGGGAATCCTTTGGAGAAGGAGGAGATGCTGCTAACTTGGCATTTGGCCAGGGGCTTTATGAGTATGCGTCTCGCATTAGTGATGCTATAGTTTCAGCTATCTCAGGTTCAGTCAAACGAGGAATTGATGAGTTCCTAGACAGAGTTTATCAGGTTATGGCTCAAGTATTTTCGGCATGGATGCCAAGGATAAGAGCCGCTTTCCAGTGGTTTGAAAACACAAAAGAGGTCATTAAGAGATGGGCAAATACCATGCATGAGAAAATCAATTGCGTGCTTGTGGGCATGGAGGACTGCCTGTACATGGGAGCTGGATTGGTAGCAGCTACTTGTATTGTCACACTGTTAGAAAAGTTTATGGTTGCGATTGGAATATTGACCAAACCGTGTGGTGCGGCTACTTTGTTTCTAACTACAGCCATGGCTGCCATTTCGGCGACTTACGTCTGCACCAAGGCGGTGGAAAAGTCCGTGATGCTCACAAATTTGTTACATTTTGTGACCACAAATTGCCAAGTTGTGCTGAATGCACTTTTCAATTATGAAATGATGGCTAGGAATCAATCCCAACCAGCCGACTCGGACCAAAATAGTGAGAGTGTAGGTCAGTTTGGGGTGTCGACTATGCTTCAAGATGTTGCTAGTTTGATGTCTACATGGTCATCAGGATCAATTACAGAGGTTGGGAGAACTTTCGGCGCAATTTCTCAAATAAAAAATGGAATCGTGGCCCTAAGAGACATGATTTATTTTATATTTGAGAAGTTGAGTGAGTTGGCCCACAAAGTGCTTGGCTTTGAATCCCAGGTCTTAGCAGATTTATCCATTTTACTTGGGGAGAATGTGGCTGATTGGTTGTCAGAGTGCGACTGTATGCTAGCCTATATGTTGGAATTTAATTCCAGGAATAGGGAGATATTTGATAGGCTATCCCAGTTAATTGAGAAAGGTAGGATGATTAGAACTGGAGTCTTGCGAACTAATCATCGGGGCTCTTCTCAAGTTATGTCACTTGTAACTAAAGCATTGGAGAAATTGATAGAGTTGCACAATTCTATTGTTATGTCAGGATCTAACACCACGAGGAAATCTCCTTTTATGGTCTTCTTTACCGGAGCTTCTGGCACAGGCAAGACTTCTGTGGTGCAAAGAGTGGCTATAAATTGGTTGCAAGAAGAGCAACTAGGCACTAGTGAGGTGTACGCTCGTAATGGGCAAGACCCTTTCTGGTCTGGTTATAAACGACATGCCGTAGTCACCTACGATGATTTTGGAGCAGTTCCTGGAACCGTTTCCAATGAAGCTGAGATCATAAATGTCGTCTCAAGAAACCCCTACGCGACTGTTATGGCAGGGTTGGCAGAGAAAGGAATGTATTTTGACTCAAGGTTAATTTTAGCCAGTAGCAACTTTCTAGCGGCCAACCCAGAATCGGGAGTGCATGATTCTGAAGCCTATGAGAGGCGAAGACATGTGGTGATCAGGGTTTCTTTAAAACCAGGAATCCCTTATAATGCTGGAGACCCTTGCGCAAATCAAACCTATACTTTGCTAGAGTCCAAGTCTCCTTTTCGGGAAATGCAAACTTTTGAGTCGTATGCTGAATTGTGGTCCTATTTGTACACTAGTTTCAAGGCTCACGAGGAACAGGAGGAGATGTACTTGAATTCTCTTCCAATATTAGATTCTGATAAAAAAGAAGCATTAGAGGGCCTAATTGGACTGACAGTGATAGCCACATCGTTTGCTCCTAAAGCTGTCATGCAATATGGAGTTGACAATTTTCCTGGTCATCATTATTTGATATCAGATGGAGAAAGATGCTTTTATTGGCATGGAGAAGGATCCGTTAAAGTGGTAGATGTTGACAAGATGCATCTGGCCAAACAGGATGTTGCCCAACTTAAGCAGCAAGGACTTGCAACCGCAATGATGTACAAGGATTTGGCCAAAGCCTTTCCTACAATTAATTCTTTGGCAGTTTTGTATGCAAAGAACATTGTGGTGAAGCGTTGGATTGGCCCAGACTTAGAGCCTACAAAGAATTGTGAAGACATCTATATGCGCGAGCAAATTGGGAAATTACCTGAGTGGCAGCGAGCATATCTTCATGTGCTCAGTAAATATCTTTCAGTCCAAAGCCCGCGGGGTTGGTTTATGGAGTGCCTAGAGGAGACGAAAAGAAATCTGAAAACCACTTACTTGTGGGAGTATAAGCAATGGCCACTACCGCTGAAGTTAGCACTTGGATCTCTGATTGCTATATTAGCGGGGGGTGCTATTTGGTATTCTCTGCAATCTTTGTGGTGTATGACAGGAGATGCCTCCTTTATAGCTGGGGCTGCTACTGTATTCTCAGTCTCTTCGATGGCAGGTCAAAGTGACATACCCAATCGTGACAACTCGGAGAGGTCTTTCCGAAATAGAAAGATCCGTGCAAGAACTTGGCAAGGACAGAGTTCATGTTTTGGTGATTCAGCTTTGTGGATTGCAGAAACGTGTGTCGCCACATTGTCTTTTTCAAATGTGAGAACACAGGTGTGTTTGGCCCCAGGAAGAGGCTTCTTTGGAGTCAATCATTGTTTGGCTGCTATACCTAATGGTATCATGGTCAAGTTGGATTCCAGCATAGGCGTTACTTATTTTGTGTGGGAGAAGGAAAAGTTACTGACATTTGAAGGTAATGAAATCGCATTGTATGCAACTAGTACGTTACCTAAAACAGTGGACTCCCTCCTTGGAAGAATTCATTTTGATGTGGAAACGCTTCCAAAAACATTTGGGGCTGTTTTTTTCTCATTCAAATATGATCCCATGACACAACAGATGGTGCCTGAACTAGGGAGTGTCACATGTAAGGTCCAGAACAAAGCTTACACGTTGGCCCATGGCGAGTATAGAAGGGAAATTCCCCAAAGCTTGTCTTATGAAGCCAGCACCGTGGCGGGCGATTGTGGGTCTTTGATTCTTGCTGAGATAGAAGGTAAATTTAAACTTGTTGGGATGCATGTGGCGTTCAATGGAAAGGAAGGTAGTGCTAGTTTTATACCTTATCATGCTTGCTTGAACCAACAAGTGGGGCAAGGAGATTTCGTGTTAAAATACCAAGAATGGGCTGAGCCAAAGATTATTGGGCCTGGCTGTAGAGCCATGGGCTTAATTGATCCTGAGCATGCTTTGTCCGCGAGTGGGAAAACCAGCTTTGTTGAAACACCTGAAGAGTGGCATCTGGATTTTCCATGTGATAAAATTCCTAGTGTGTTGACACGAGCGGATCCTAGATTGGCTGGAACGATTCATGCGGATTATGATCCATTTGCAGTGGGCATGAGCAAGTATGCTAAAGAAGCTGGACCTTTTGAGGCAGAGAGTCTCAAACAAGTGTGCTTGGGTATATCTGAGGTGTGGGAAGATGCTTCAGCTGAATTCCCTATGGAAGAAGTGGACTTGGATACAGCCATCAATGGGTTGGAAAATGTGGAGTTTTTTGACGCCCTCGTTCTCGGAACATCTGAAGGATTTCCTTATCGTTTGGACAGAGGTCCCGGTGATAAAGGTAAGAGCAGGTATGTTTCAGGTGAGAGTGGTAGTCTTAAAATAACGGATGAGCAAATGTTATCTGATATTGCATGGTTTGAGGAGACTAGCAAAGTGCAAGTTCCTGATCTCTATTGCATAGAATGCGTGAAGGATGAGCGTCTACCCATCCGCAAGGTCCTGCATGAACCTAAGAGTAGACTGTTTACAGTGCTACCTATGTCATTCAATATAGTTGTGCGGAAGAAATTCCTAAATTTTGTGAGGTTTTTCATGAAACGAAGAGATGTGCTTCCAGCACAAGTAGGAATAAATCCTTACTCTCGAGAGTGGACTCGCGTGGCAAATAAGCTTCGGAGCAAAGGAAATAACATACTGTGTTGTGACTATAGTCGGTTTGATGGATTCCTACCCAAGTGCATCATGAAAGAGATAGGTGGCATGATTGCTAGAGTTATGAAAGTGGATCAAGCGACAAAACGGCAGATTGAAAACATTATGCTGGCTTGCACAAGTCGTTATGCTATGTGCAACCGAGTTCTTTACAGAGTGGAGAATGGAATACCTTCTGGTTTTCCATTGACAGTGATTGTTAACTCCATCTTGAATGAAATTCTGGTGAAATATGCTTACTGGCATTGTTTCGCAGACAATTTGCAAGTTCAAAGCAATTTTGACGCCCATGTGTCTATGGTTGTATATGGAGATGACAATTTGATTTCAGTGTCAGATGCAATTAGCTCTAGATTCAATGGAAGCTACTTAGTGTCTTTCATGGAAGGATTGGGTGTCAAGGTTACGGATGGAGTCGACAAAACCAAGGTAGGAATTGAATTCAGAAGCCTTGACAATTGTGATTTCTTGAAACGCTCTTTTAAGATGAGTCGTGACGGGACGTGGCGTAGTCCTATGGCGAAAGAGAGTTTGTGGCCACAACTCCATTTTGTAAAAGCGAAGAAAATTGAGATGGCAGAAGCCTATATCAACAATTGTAACAACATTCTAAGGGAATTGTGGTTGCATGATGTAGAAGAGGCAAGAGGCTTTCGCAATAAAGTGCTTAGGAACTTGAAATGGATCGGTCATGACCAGCTACTCAATATGCAGCAATTGGCTGTGTTTCATGATGAGCAAATGAATGGTGTCGCTGATTTCATGACGGCATGCACTACAGTTGACAATTTTTCCTTGATGGATCCTCTAATTCCGGGGGTGCTACCTGTTAAGACACATGAAATTATGCCTAGGGTTTTTGTTGCGGCGGAGAAGCATTTCGAAGGCAATTTCGAAGAGTACTATACGATATCGATCACCACCAGCCGCAAATTTGAGGAGGACAAGGGATTTGTGCTAATTTTTCCTTATGGTGTTGGTAGAGGAGGGCTTCCAACTACGCAATTTATGAGGGAAAATGTTTTGAGAAAAGGATGCTCAATACAGAAGAAATTTAAGCAAGCTTACGAGAGTGGAAGGAAATTGTTGTTTATATCTCAAAGTTCTGTAATCCCCGCCTATGTGTTCTCCGTCATGCTTCTATACTCCATAGGGGCAATCAACAGACTCTCTAGTAATAAAGCTCTGACACAAGCCATGCAAACTTGCAAACGCTTGGAATATTTGCCCAAGGAATTTGGAGAGTTTTTCTGAACGCTTATTTGCAATCACAATGTGCAACATTTATATATATATCTACTACTTGTACTGTGTAGTTAGTACTACGCAAGGTTGAAATGCCTTCCTCATGTAAATTTCAGTAGTGGAGG

>RNA2-ZRGX1 [organism= Broad bean wilt virus 2] [isolate= ZR-GX1] segment RNA2, complete genome

GTTTTAAAAAATATTTAAAACAAACAGCTTTCGTTCCTTTCAACAGCTTTCAGTCACTTTCTTGCTTTCAAACAGCTTTCTTTCAACCAGCTTTCTGTTACGAAATTGCTTTCTGGCTACTGAAATTTTCAAATTGAACCCGGAAAAAGGGGGTGTGATTTAAAGCGCACCATATGATTTGAAGGTTTCTCTTTCGTTATGCACCCAGAACACGTAGCCCTTTTGGATACATTAGGTTCTGAGATCATAACATGTCTTTTTCTAGGTTTTTTAAGCAACTTAATTTTTCCTTCAGCTAGTGGTTTTCTTTTGTGGGCTTTCTTTTTGTACAGTTGTTGGCATGTGTTGAAAATTGAATTTAAGTATATTGTTAAACCCTTCTTTGAAACAATATATACGAATAGTTTTCAATATCACACCATTGACTGGGAGAACGCGTACACGGCGAGATCCAGAAGTTTGTGGGAACAGATAACAAATTATAATTACTGCTTTAATTTCCCCCAGACCAGTACAGAAGAGTATCCTTCCGTGTATTCACCAAGGTTCACTTTTGAGGAGCTCAAAATAATGGAAGAGGCCAACATAACGCCAGTGCACACCATTCCCAAAGAAACTTTGCTCAAGAGAGCAAGTGACTATAAACTGGCAGTGGAGGGTAAAAAATCCATTTTGCCCAAAGTACAAGACCTTTATGAGACGGATAAGTGGCATTCTTTTAGAAGTAAGTTATCTAAAAATTGCCCCAGTTATGTGATCACATCAGAGATGGCAGTGGGAGCAATGTCAGGTGCGGGCAACACCAAATTGTCAATACCAATTGTGGAAAAATACTCTGAGGAAGTTGCAGATGACAGATTGCCAGAGCATGTTCGTGCTAAAGCTGACCAGATTATGGTGGCAGCTATTGAACTTGTTGCTGATGGCTTTGCTTCTGTCAATTCGGATGTCACAATGGCTGGAGCACTGTACGATAAACGTCACAGGACTATTGCAAGTTCCTTTAAGGGAGCTTTTGCCTCTAGGGCCAGTGGTGTGCCCTCTCATGTAGTGTACTTTCCAATGCACAGAGTTCCGGTGAGTGATGACCCAAACACAACGCTTGAGTTGTCAATGGTTAGTCGGGACACGGATTTTGATGAGTGTTATACTCTGGCGAACGTGTCCGCCCGAACTTTATATGTGCGAGCTAGAGGCCCGGAAAAGGTCACTGAGACGCGCCACTTGCTCAAAGCAAAGACCGAAGATGTTGTGAAAGCTCGTCAGTTTGCGAGTGAAGCACAAGTGGCTTTTGCCACACCAAGATTGTTTCCTGAAGTCAATTTAGACAATTATAAACTGCCTGGACCAAGCAATCTGCTGCAAACTGAAGCGGTCACGACCAGTAAAGGAATTTTGTTCCCAAAACCGAGGTTTAAAGGAAATGAAGTGGTTTTGAACTATACTGGATCTCACAATTATTTGGAGAAGATTTCTAAAGATCCAGGGGGAAAAACCCAAAATGGAAAATATCATGTGAGCTCTGTGGAAGATTTAGGTTGCCTTTCTGATGAAGATGGAAAGGATTATAGATTTGGCCAAGGATTAATGGAAGAGGATGTGTTGGATGTTCAAACTAACAACTTCGCCATTAGTTCAGCGACTGAGACTATGAGGCTTCTTTTCAGTGGTTTTGCCACAATACCACTTAATGTTGTCCCTGGCACTAAACTTACTGTTGCCTATTTGAATGAGCTTTCACGACATAGTGCAGTGCACACAGGGTTGCTGAATATGTTGAGCAAAATTCCAGGGTCTTTGAAAGTTAAGATTAATTGTCAGGTCGCCCCAACTTGTGGCATTGGCCTTGCAGTTAGCTATGTGGAGGGCAATGAGAGTGCTCAACTTGGATCGAATTTGGGTCGATTGCTAGGTATTCAACATTACAAGTGGAACCCTGCTATAGAGCCATTCGTGGAATTCATGTTCAAGCCTTTCTCTTGTGCAGATTGGTGGAATATGCATTATTTGGGTTCCTCTAAGTATTCCCCAGTCATGGTCATCCAGACTCTGTCAAAGTGGCTTAATGCACCAAAAGTTGATGCGCGATTGAGTTTTGCAATTTACTATGAGCCCAATGTGATCATGCCAAAGCAAATAGCCAGCATAGGACAAGCTCCTTCATTCATGTTTAGGAAGGAGCTTGGGACTCTGTCATTCAAACAAGGGCAGCGTATGGCATATGCTTTTGAGGTCAATTTTGGTAAGCCACAAACTGATGGGAAGGAAGTCACTCTAACGTTTGCATCTTCCTATTGTGGTCTTAGTCAATATATGCAGGCGGATGTCATATTGGATTTCACGCTCATGAGTAGTCCGATGATAGGAGGGACTTTTTCCATTGCATACGTCGCAGGTGCTTTTATAGAGAGAGTAGACAATATGCAAACTTTGGATTCATTGCCACATGTGGATTTCACATTCTCATCTGGATCCAAGAGTACTAGATCAGCACGCTTCCCCAAGGAAGTTTTTGGTGTTTACCAGGCTTTGGATAGATGGGATCTTGATGCCACCCGAGGAGATGATGTTTCAGGAAACTTTGTGATTTACCAAAGAGACACTGTCTCTAGCGCTTTGGAGGGTGACTTAACTTTTAGAGTGGCAGCTCGCCTCTCAGGTGAAGTGCAATTTTATGGAGTTAGTGTTGGATATCCAACTACAGTAACTCGCATTGGCAAGGGCAAAACACAGAGCAGATCATTGGGGCCTGATTTGCGAAAACCACTGCGATACATGATGGGTCAGTCCCATATGTCTCCAGGGGATTTTAAGTCAGTTCGCTTTGTTATGGGGTATTGGAAATATAAAACTGGTGTCTACCCAGGGAGTAAGGCTGATGAGGACATACATCCCTTTTCCCTCAAAATGCGGTTGGATGGATCTAAGAGTAGTGAGCATTTTGAAATCATACACTCTCCATTTGTTCGAATGCTGCAAAATTGCGCTTGGATGAAGGGCACACTGAATTTCCATGTCGTTGCACGTGCTAGCTCAGACTATATGAGCTATCGTAGAACTTCACAACTATTGGTATCAGCCCATGAAAACAGTCTTAGCTCGAATCAGTTTTACTGTGGAACTTTCAATGGTCCGAGTGGAGAGTTAATGTTTTTTAGGGAAGTTGTTGGGCCCGTGGAAGGGTTTGCATCAATGGGCTGGAATGTGCGAGGCAGTAAAAAGTTTTATAAAGTGAATATAGAATTAGGAAATGTTCATGAGTATGAGACTGTGATTCTGTATGGGCAATTTGAACAAGATGTGGAATTTGCAGGGCAGCAAAAAGGAGGACATTACTCCTTGGAAAAGGAAGTTCCTATCTTTAAATCCATAAAATTTTAAATTAATTAACTTGAATTTTGCATACTGTACAATGGCAGTTTGATTTAATAAACAGGCGTAGGCCCCAAGCCTCCCATTAAAATGGGTTCCATGGTTTTAGGTGTTAATTTTATGTTGTAAATTTACTACTTTTTGAAGCGTTAAGATTTAATGCTAGATTGAAATGTCTTATTTTAAATTTCAGTAGTAAAGC

>RNA1-ZRGX2 [organism= Broad bean wilt virus 2] isolate ZR-GX2 segment RNA1, complete genome

GTTTTAATAAAATATTGAAACAAACAGCTTTCGTTCCGAAAACAGCTTTCAGTTACTAAACAGCTTTCGTTCCGATTAAACAGCTTTCAGAAACAAACAGCTTTCAACTTCATTTGTAGATTAACTTTTTGCGTGACTTTGGGAAGAACCCGGAAAAAGGGAGTGTGATTTAAAGCGCACCATATATTCCAAAGATCATTTTGAATTTCATTTTGCGATCTTAATTTTAAGATGGATTTCAGTGTGATGCAGGTTGTTGTGGGTTTTTTGAAGACTTCGATGGGTTTACAATCCATCAAGGATATTGTTCAGAAAGCACAAGTGGCGGAGAAGGATAAAGTGTTGCTTCATATCCACCTGTGCTTTTTCCATGCTAATGAAATGGCACGGGATTTGAATGAAGGAATGAGCATGAGCCAGATACAAAATTCTGCAGCAATAAAATATCGAGCTGCAATTGTAATGCGCCATGTCAAGATGAATGTGGAGACAGGGAAATACGACAGGGAGCGCATGTTGCAGTATAACAATGAAACATGTGTCAACTGGTTTTCATGTGAATTTTCAGAGGATACAGCCACGGACACATCAGGTGGAAGTGTTCAAGAAGAGGAAATCGTCAATGAATTTCTTAAGAGCCGTATCAGCACTGGGGAAGCTGGGCAAGCGTCTACAGGAAAAGGCCCGAAGTATGCTTTTGGCCAAGGGTTGTATGAGTACGCAACTCGAATTGGTGATGTGATAGTTGCGGCTATCTCAGGTTCAATTAAGAAAGGGATAGATGAGTTCTTGGATAAAGTCTATGCGGTTATGACACAAATATTTGCTGCTTGGATGCCCAAGATCAGAGCCGCTTTTCAGTGGTTTGAGAACATTAAAGATGTTATAAAGAAGTGGGCAAACACCATGCATGATAAGATTAATTGTATTTTGGTCGGTATGGAGGATTGCTTGTACATGGGAGCTGGGCTTGTTGCAGCCACGTGCATTGTGACGCTGCTTGAGAAATTCATGGTAGCCATTGGTATTTTAACAAAACCATGTGGAGCGGCAACTCTTTTCCTAACAACTGCAATGGCGGCCATTTCAGCTACATATGTTTGCGCTAAGGCTGTGGAGAGATCAGTTATGTTAACTAGCCTGTTGCAATTTGTGACTTCCAATTGTCAAATCGTGTTGAACGCTTTGTTTAATTACGAGATGACGAGGAAAGAGCAATCTTCAGGCCATGATAGGGACGAAGCTCCGGCAAGCCTTGGTCAATTTGGAGTTTCCTCAATGTTGCAAGATGTCGCCAACTTGATGTCGACGTGGTCTATGGGAACTGTGACAGAAATTGGAAGAACATTCGGAGCCATCTCGCAGATCAAGAATGGAATTGTGGCTCTAAAGGATATGGTGCACTTTGTTTTTGAGAAATTGAGTGAGTTAGCTCATAAGGTGCTAGGATTCGAGTCCCAGGTGTTAGCGGACCTTTCAATTTTGCTTGGTGAGAATGTTGCAGACTGGTTGTCTGAGTGTGATTGCATGGTGGCCTACTTGCTTGAATTCAACTCTAGGAATAGAGAAATTTTTGATAGGCTTTCACAATTGATTGAGAAAGGTAGGTTGATAAGGGCTGGTGTGTTGCGCACGAGTCATCGTGGCTCATCGCAAGTTATGGCACTTGTTACCAAAGCTTTGGAAAAGCTGATTGAATTGCACAATTCTGTTGTGATGTCGGGATCAAACACCACTAGGAAGTCCCCGTTTATGATTTTTTTCACAGGGGCTTCTGGTACAGGGAAAACATCGGTTGTTCAGAGAGTCGCCATAAATTGGTTGCAAGAAGAGCAGCTTGGCACAAGTGAAATTTATGCACGCAATGGTCAAGACCCATTTTGGTCAGGTTATAAGAGGCATGCAGTCGTCACATATGATGATTTTGGTGCTGTGCCAGGTACAACCTCAAATGAAGCTGAGATCATAAATGTGATCTCAAGAAATCCATATGCGACAATGATGGCAGGGTTGGCTGAGAAAGGAATGTACTTTGATTCAAGATTGGTTTTGGCTAGTAGTAATTTCTTGGCTGCCAACCCCGAGTCGGGAGTTCATGACTCTGAGGCATATGAGAGGCGGAGGCATGCTGTTGTTAGAGTGTCTCTTAAACCTGGAGTGCCGTACAATGCAAATGATCCGTGTGCCAATCAGACATACACTCTTCTTGAGTCCAAGACACCTTTTAGAGAAGTTCAAACTTTTGAGACATATGCAGAGTTGTGGTCTTACTTGTACACAAATTTTAAGGAGCATGAGGAGCAAGAGGAGTTGTATTTGAAATCCTTACCTATTCTGGATTCTGATAAGAAGGAGGCTTTGGAAGGCCTTGTAGGCCTTACAGTGATAGCCACCTCTTTTGCTCCGAAGACTGTGATGCAGTTTGGTGCAAGCAAATTCCCGGGTTATCATTTTTTGATTTCAGATGGGGAGAGATGCTACTTTTGGCATGGAGATGGTTCAGTTGAGATAGTCAGCGTGGATCAAATGCATTTGAGCAAGCAGGACATCGCACAATTGAAGCAGCAGGGTTTGTCAACTGCAATGATGTACAAGGATTTGGCCAAAGCTTTTCCAACTCTGAATTCACTAGCTGTGTTGTATGCCAAAAATATTGTGGTGAAGCGCTGGGTGGGACCAGATCTAGAACCAACTAAGAGTTGTGAAGATGTGTACATGCGGGAGCAGATCGGAAATTTGCCAAAATGGCAGAGAGCATATTTGCATGTGCTCAGCAAGTATTTGACAGCTCAAAGTCCACGAGGTTGGTTCATGGAATGTTTGGAGGAGACAAAGAAAAATTTGAGGGCAACTTATCTGTGGGAGTATAAACAGTGGCCATTGCCTCTGAAATTGGCTCTGGGTTCTTTGATTGCCATTTTGGCAGGAGGAGCTATTTGGTATTCATTGCAATCCTTGTGGTGCATGTCTGGGGATGCTTCTTTTATAGCAGGAGCTGCCATGGTTTTTTCAGCATCTTCGTTCACGGGACAAAGTGATATACCCAATCGAGATAACTCGGAGAGGTCATTTAGAAACAGGAAGATACGTGCAAGAACCTGGCAAGGTCAAAGCTCATGCTTTGGAGACTCAGCATTATGGATTGCGGAGACGTGCGTGGCAACACTAACGTTTTCCAACGTGAGGACACAAGTATGCCTAGCCCCAGGTAGAGGTTTTTTTGGAGTGAATCATTGCTTAGCGGCAATTCCTGCGGGAGTTATGGTAAAGATGGACTCGAGCATAGGGGTCACATATTTCATATGGGAGAAGGAAAAGTTGTTGCAATTTGAAGGCAATGAGGTGGCATTGTACATGACAAGCACATTGCCCAAAACTGTGGATTCTCTTCTGAGCAGAATTCATTTTGATGTGGAAACTTTGCCTAAGACCTTTAGTGCTGTTTTCTTTTCTTATAAGTATGATCCAATGACTCAGCAAATGGTACCCGAACTTGGGAGTGTGACGTGTAAAGTTCACAACAAGGCTTACACATTGGCTCATGGGGAGTACAGGCGGGAGATCCCTCAAAGTCTCTCTTATGAAGCCAGTACTGTTGCTGGTGATTGTGGTTCTCTGATAATGGCTGAAATTGAAGGGAAATTCAAGCTAGTGGGTATGCATGTGGCATTCAATGGCAAAGAAGGGAGTGCAAGTTTTATGCCCTACCATGCTGGCTTGGATCAAAAGGTTGGTCAGGGAGACTTTATGCTTAAATATCAGGAGTGGGCTGAGCCGAAAATTTTGGGGCCGGGTTGTAGAGCAATGGGTCTTATAGAGCCTGAGCATGCTTTGGCAGCCAGTGGGAAAACAACATTTGTGGAAACCCCGGAGGAGTGGCATTTAGATTACCCATGTGATAAACTTCCAAGTGTGCTTGCCCGAGGGGACCCTAGATTGGCAGGGACGGTCCATGCAGATTATGACCCTTTTGCTTCCGGTATGAGCAAATACGCAAAAGAGGCGGGCCCCTTTGATGCTGCAAGCCTTAAGCAAGTGTGTTCAGGGATAGTTGAAATCTGGGAAGATGCTTCAGCAGAATTTCCCATGGATGAGGTTGATCTGGACACTGCCATTAATGGTTTGGAGAATGTCGAGTTCTTCGATGCTTTGGTATTAGGGACATCTGAGGGATTTCCTTATAGGTTGGATCGGGGCCCTGGCGATAAAGGAAAGAGTAGATATGTGTCCGGTGAGAGTGGAAATTTGAAGATAACTGATGAAGGAGTTCTCTCGGATATAGATTGGTTTGAGGAAGTGAGCAAAACACAGGTACCAGATCTTTATTGCATTGAATGTGTGAAAGATGAGAGGTTGCCGATTAGGAAAGTGCTCCATGAGCCTAAGAGCAGATTATTCACAGTCCTTCCAATGTCTTACAACATTGTTATCCGGAAGAAATTCTTAAATTTTGTTAGGTTTTTCATGAAGAGGAGGGATGTTTTACCAGCTCAAGTTGGCATTAACCCTTACTCGCGTGAATGGACTCGAATGGCTAACAAGTTGTTGAGCAAAGGGAATAACATTTTGTGCTGCGATTATAGCAGATTTGATGGCTTTTTGCCCAAATGCATCATGAATGAAATAGGGGACATGATAGCCAGGGTAATGAAGGCGAATGAGGAGTCTAAGGCGCAAATTAAGAACTTGATGCTTGCATGTACTAGTCGGTATGCAATGTGCAATCGAGTTTTGTACAGAGTTGAGAATGGCATTCCCTCTGGTTTTCCATTGACTGTCGTCGTGAACTCTATTTTGAATGAAATATTGGTCAAGTATGCTTACTGGCATTGTTTTGAGGATAATCCAAATGTGCAAAGCAACTTTGACGCACATGTGTCAATGGTGGTTTACGGTGATGACAATTTGATTTCTGTATCAGATGCTATAAGTTCAAAATTTGATGGGAATTTCCTTGTGAATTTCATGGAGAGTTTAGGGATAAAAGTGACTGATGGCATCGATAAGACGAAGATAGGGATTGAATTCCGGAGATTGGAGAATTGTGATTTCTTGAAGCGCTCATTTAAGATGAATCCAGATGGAACATGGCGTTGCCCTATGTCCAAAGAAAGCTTGTGGCCACAGTTACATTATGTCAAGGCAAAGAAACTGGAAATGGCTGAAGCTTATATCAACAATTGCAACAACATTCTTCGTGAATTATGGCTGCATGATGTCAAGGAGGCAAAGGAATTTCGCAACAAGGTGTTGAGGAGCTTGAAATGGATTGGTCATGACCAATTGCTCAATATGCAACAGCTAGCAGTGTTCCACAGTGAACAAATGAATGGAGCTAGCGACTTTTTGTCTGCTTGTGTGACAGTTGATAGCATTTCCTTGATGGATCCTTTGGTACCAGGTATGTTGCCAGTTAAGACTTGTGAGATCATTCCCCGTGTTTTCGTGGCAGCAGAGAAGCATTTTGGAGGAAACTTTGAAGATTACTTCACAATTTCAATAACCACGAGTCGCAAATTTGAAGAGGATAAAGGCTTTGTTCTTTTGTTCCCTTATGGAGCTGGTAGAGGAGGTTTGCCAACAACGCAGTTCATGAAGGAAAATGTTGTAAGAAAAGGATGCTCGATACAGAAGAAGTTCAGGCAAGCCTATGAAAAAGGAAACAAGATTTTGTTCATATCGCAGAGTTCAGTAGTGCCTGCTTACGTCTTCGCTGTGATGCTTTTGCATTCCATTGGAGCAATTAACAGGTTGACAAGCAACAAGGCTTTAACTCAAGCTATGCAAACTTGCAAAAAGTTGGAATATCTACCTAGAGAGTATGAAGAGTTTTTTTGATTGCTTGTATGTTGTATGTATGTATGTTATGTAAATCACTACTTATATTGTATGGTAAGTACTATGCAGGGTTGAAATGCCTTTTCCTAAGTAAATTTCAGTAGTGGGGG

>RNA2-ZRGX2 [organism= Broad bean wilt virus 2] [isolate= ZR-GX2] segment RNA2, complete genome

GTTTTAATAAAATATTAAAACAAACAGCTTTCGTTCCGAAAAACAGCTTTCAAATTTCAAACAGCTTTCAGACACTTTGGGATTTTCAGATTGAACCCGGAAAAAGGGAGTGTGATTTAAAGCGCACCATATCATTTGAAAATTTCGTTTTGATTTTTACTTTCTTTGCTAGTGTTATTTATGCAAATATGAGGTTTTGTTGTGATGAATCCTGAGTTAGCAGCTGTGTTAGATAGGTATCTATCTGAGATCGCAAGTAGTTTATTTTTAGGTTGGATTATAAATCTCCTCTTAGTTTTCTTTTGTTCCGCTAAGAGTTGTTTCTTGCTGTGGGCCGCATTTCTTTACATCAATTATTACATATTGAGATTTGAATTTGCATATATCGTTGCGCCCTTCCTTAAAACGATATATTCAAATAGTTCTCAATACCACACTGTTGATTGGGAAAACGCTTACACGGCACTTCCCAAAAATTTGTGGGAACAAATAACTGATTACAATTACTGTTTCAATTTCCCAAAACCCATTGGAGAGGGCTTTGTGTCGGTTTTTTCGCCGCGTTTTACGCTTGAAGAACTTATTGCAATGAATGAGGCAAATATCACTCCAGTTCACACAATTCCGAGAGAAACCTTGCTCAGAAGAGCAAGTGACTATAAATTGGCTGTGGAGAGCAAAAAGTCCATACTGCCCAAAGTTCAAGATTTATATGAGATGGACAAATGGCATGCTTTGAAGAGTAGGTTGAACAAGAATGCGCCTAGTTATGTTGTGACTTCAGAGATTGCAGTTGGAGCTATGTCAGGCGCTGGGAATGTAAAATTGGCGCTGCCCGTGGTGGAAAAATACACTGAAGAAGTAGCAGATGACAGATTGCCTGACAAAGTTCGCGCCAAAGCTGATCAAATAATGGTCGCGGCCATTGAGTTGGTGGCAGATGGCTTCGCCTCAGTTAATTCTGATGTTACTATGGCAGGTGCGCTTTATGATAAGCGCCACAAGACAATTGCTAGTTCTTTCAAAGGAGCTTTTGCATCCAGAGCGAGTGGAGTCCCTTCTCATGTCATTTATTATCCAATGCATAGAGTTCCTTCAAATGATGATCCTAATACAACTTTGGAACTTTCAATGGTTAGTCGCGATTCCGATTTTGATGAGGGTTTCACGTTGGCTAATATCTCAGCACGCACTCTTTATGTTCGTGCAAAAGGGCCTGAAAAGGTGACTGAGACAAGGCATCTCTTGAAGGCCAAGACTGAAGATGTGGTGAAAGCACAACAGTTTGCGAGTGAAGCACAAGTTGTGTTTGCCACTCCTCGGCTCTTTCCTGAAGTCAACCTGGATAACTACAATTTACCTGGGCCTAGCAATGTGCAGCAAACAGAGGCAATTACCACCAATAGGGGAATTCTTTTCCCTAAGCCAAAATTCAAAGGGAATGAGGTGGTGCTCAACTACACAGGGCCAACAAAAGTTAGAAATGTTAGTGTGCAGAGGTCTGGGCAGCAAGAGTTCAGCAGCAAACCATATGTGGAAAGTGTCGACGACCTTGGATGTTTATCAGACGAGGATGGCAAGGATTATAGATATGGCCAAGGCTTGATGGAGGAGGACGTTTTGAACGTTCAGACCAACAATTTCGCCATTGAGTCTGCTACAGAGACCATGCGCTTGTTGTTTAGTGGCCACGCGAGCATTCCTCTGAACGTTATACCTGGAACGAAGCTTACTGTGGCCTATCTTAATGAATTATCCAAGCATAGTGCTGTGCATACTGGTTTGTTAAATATGCTTAGCAAGGTCCCAGGTTCTTTGAAGGTCAAGATAAATTGCCAGGTTGCTCCTACATGTGGGATTGGATTGGCAGTCAGTTATGTTGAAGGCAATGAAAGTGCAAACTTGGGATCTAGCCTGGGGCGCTTGTTGGGCATTCAGCATTACAAGTGGAATCCAGCTATAGAGCCTTATGTGGAATTTGTTTTCAAGCCCTTTTCCTGCGCAGATTGGTGGAACATGCATTATTTAGGATCATTCAAATATGCACCTGTGATGGTCATCCAAACATTATCCAAATGGTTGAATGCGCCAAAAGTGGATGCCAAGATGAGCTTTGCCATTTATTATGAACCCAGTGTGATTTTGCCCAAACAAATAGCAACCTTGGATCATGCCCCAGCATTTATGTTTCGCAAGGAACTGGGGACTCTAGCTTTCAGGCAAGGGGAACGGGTGGCATATTCTTTTGAAGTTAATTTTGGTAAACCTCAGACGGACGGAAAGGAAGTGACATCGACTTTTGCCTCATCTTATTGTGGTTTGAGTCAGTACATGCAATCTGATGTGATCTTGGATTTTACTCTTATGAGCAGTCCTATGATTGGAGGCACTTTTTCAATTGCATATGTTGCAGGTGCATATATTGAGAAAGCTGGGAACATGCAAATTCTTGATTCATTGCCCCATGTTGATTTCACATTTTCATCAGGTTCCAAGAGCACGCGTTCTGTGCGATTTCCGAAAGAAGTTTTTGGGGTACATCAGGCATTGGATAGGTGGGATCTGGATTCAGCAAGGGGAGATGATGTTTCAGGTAATTTTGTGATTTACCAGAGAGATGCAGTCTCAAGTGCTCTTGAAGGGGAATTAACGTTCCGAATTGCTGCTCGCTTGTCTGGAGACATCAATTTTGTTGGTGTCAGTGCAGGCTATCCAACAACAATTACGCGAATTGGCAAAGGTAAGGCGCAAGGAAGATCGCTGGATCCTGAAATTAGAAAGCCTCTAAAGTACATGATTGGTCAGTCTCATTCAACACCACAGGATTTTAGTTCAGTGCGCTTTGTGATGGGCCGCTGGAAATACAAAGCTGGTTTGTATCCAGGGAGCAAATCAGATGAAGATATTCATCCATACTCCCTCAAAATGCGCCTCGATGGCTCGAAGAGCAGCGAGAATTTTGAAATTATTCATTCCCCTTTTGTTCGGTTGTTGCAAAATTGTGCATGGATGAAAGGAACTTTGAAATTCTATGTTGTGGCACGAGCTAGCTCTGATTACATGAGTTACAGAAGGACCTCTCAATTGACAGTTTCAGCTCATGAGAATAGTCTTAGCTCTAACCAATTCTACAGTGGAGTTTTGACAAGTCCTAGTGGCGAATTGGGTTTTTCCAGAGAGGTTGTAGGCCCAGTGGATGGCTTTGCATCTATGGGCTGGAACGTGCGTGGGAGTAAGAAGTTTTACAAAATTCATGTGGAAATGGGGAATGTTCATGAGTATGACACTGTGATGTTATATGGGCAATTTGGCCCGAATATGGAATTTGCTGGCCAACAGAAAGGCGGTCATTATTTGCTGGAGAAGGAAACTCCGACATTTAAGGCATTCAAATATTGATAAACAAGTTTGTTTTCTTTGTGTGTGTGCTCAAATAAAAAGGCGTAGGTCACTAGCCTCCCATTAAAATGGGTTCCATGACCTGTTAAATTTAGAAAATTACTATTTGTTAAACATATGTGTGTTATGTTGGGCAAAAGCTGCCTATGTAAGAGCTTTAATAGTGTTTC

>RNA1-ZRHB [organism= Broad bean wilt virus 2] isolate ZR-HB segment RNA1, complete CDS

GTTTTAATAAAATATTGAAACAAACAGCTTTCGTTCCGAAAACAGCTTTCAGTTACTAAACAGCTTTCGTTCCGATTAAACAGCTTTCAGAAACAAACAGCTTTCAACTTCATTCGAAGATTAACTTTTTGCGTGACTTTGGGAAGAACCCGGAAAAAGGGAGTGTGATTTAAAGCGCACCATATATTCCAAAGATCATTTTGAATTTCATTTTGCGATCTTAGTTTTAAGATGGATTTCAGTGTGATGCAGGTTGTTGTGGGTTTTTTGAAGACTTCGATGGGTTTACAATCCATCAAGGACATTGTTCAGAAAGCACAGGTGGCGGAGAAGGACAAAGTGTTGCTTCATATCCACCTGTGCTTTTTCCATGCCAATGAAATGGCACGGGATTTGAATGAAGGAATGAGCATGAGCCAGATACAAAGTTCTGCAGCAATTAAATATCGAGCTGCAATTGTAACGCGCCATGTCAAGATGAATGTGGAGACAGGGAAATACGACAGGAAGCGCATGTTGCAGTATAACAATGAAACATGTGTCAACTGGTTTTCATGTGAATTTTCAGAGGAGACAGCCACGGACATATCAGGTGGGAGCGTTCAAGAAGAGGAAATCGTCAATGAATTTCTTAAGAGCCGTATCAGCACTGGGGAAGCTGGGCAAGCGTCCACAGGAGAAGGCCCGAGGTATGCTTTTGGCCAAGGGTTGTATGAGTACGCAACTCGAATTGGTGATGTGATAGTTGCGGCTATCTCAGGATCAATTAAGAAAGGGATAGATGAGTTCTTGGATAAAGTCTATGCGGTTATGACACAAATATTTGCTGCTTGGATGCCCAAGATCAGAGCCGCTTTTCAGTGGTTTGAAAACATCAAAGATGTTATAAAGAAGTGGGCAAACACCATGCATGATAAGATTAATTGCATTTTGGTTGGTATGGAGGATTGCTTGTACATGGGAGCTGGGCTTGTTGCAGCTACGTGCATTGTGACGCTGCTTGAGAAATTCATGGTAGCCATTGGTATTTTAACAAAACCATGTGGAGCGGCAACTCTTTTCCTAACAACTGCAATGGCGGCCATTTCAGCTACATATGTTTGCGCTAAGGCTGTGGAGAGATCAGTTATGTTAACTAGCCTGTTGCAATTTGTGACTTCCAATTGTCAAATCGTGTTGAACGCTTTGTTTAATTACGAGATGACGAGGAAAGAGCAATCTTCAGGCCGTGATAGGGATGAAGCTCCGGCAAGCCTTGGTCAATTTGGAGTTTCCTCAATGTTGCAGGATGTCGCCAACTTGATGTCGACGTGGTCTATGGGAACTGTGACAGAAATTGGAAGAACATTCGGAGCCATCTCGCAGATCAAGAATGGAATTGTGGCTCTAAAGGATATGGTGCACTTTGTTTTTGAGAAATTGAGTGAGTTAGCTCATAAAGTGCTAGGATTCGAGTCCCAGGTGTTAGCGGACCTTTCAATTTTGCTTGGTGAGAATGTTGCAGACTGGTTGTCTGAGTGTGATTGCATGGTGGCCTACTTGCTTGAATTCAATTCTAGGAATAGAGAAATTTTTGATAGGCTTTCACAATTGATTGAGAAAGGTAGGTTGATAAGGGCTGGTGTGTTGCGCACAAGTCATCGTGGCTCGTCGCAAGTTATGGCACTTGTTACCAAAGCTTTGGAAAAGCTGATTGAATTGCACAATTCTGTTGTGATGTCAGGATCAAACACCACTAGGAAGTCCCCGTTTATGATTTTTTTCACAGGGGCTTCTGGCACAGGGAAAACGTCGGTTGTTCAGAGAGTCGCCATAAATTGGTTGCAAGAAGAGCAGCTTGGCACAAGTGAAATTTATGCGCGCAATGGGCAAGACCCATTTTGGTCAGGTTATAAGAGGCATGCAGTCGTCACATATGATGATTTTGGTGCTGTACCAGGCACAACCTCAAATGAAGCTGAGATCATAAATGTGATCTCAAGAAATCCATATGCGACAATGATGGCAGGGTTGGCTGAGAAAGGAATGTACTTTGATTCAAGATTGGTTTTGGCTAGTAGTAATTTCTTGGCCGCCAACCCCGAGTCGGGAGTTCATGACTCTGAGGCATATGAGAGGCGGAGGCATGCTGTTGTTAGAGTGTCTCTTAAACCTGGAGTGCCATACAATGCAAATGATCCGTGTGCCAATCAGACATACACTCTTCTTGAGTCCAAGACACCTTTTAGAGAGATTCAAACTTTTGAGACATATGCAGAGTTGTGGTCTTACTTGTACACAAGTTTTAAGGAGCATGAGGAGCAAGAGGAGTTGTATTTGAAGTCTTTACCTATTCTGGATTCTGATAAGAAGGAGGCTTTGGAAGGCCTTGTAGGCCTTACAGTGATAGCCACCTCTTTTGCTCCGAAGACTGTGATGCAGTTTGGTGCAAGCAAATTCCCGGGTTATCATTTTTTGATTTCAGATGGGGAAAGATGCTACTTTTGGCATGGAGATGGTTCAGTTGAGATAGTTAGCGTGGATCAAATGCATTTGAGCAAGCAGGACATCGCACAATTGAAGCAGCAGGGTTTGTCAACTGCAATGATGTACAAGGATTTGGCCAAAGCTTTTCCAACTCTGAATTCACTAGCTGTGTTGTATGCCAAAAATATTGTGGTGAAACGCTGGGTGGGACCAGATCTAGAACCAACTAAGAGTTGTGAAGATGTGTACATGCGGGAGCAGATCGGAAATTTGCCAAAATGGCAGAGAGCATATTTGCATGTGCTCAGCAAGTATTTGACAGCTCAAAGTCCACGAGGTTGGTTCATGGAATGTTTGGAGGAGACAAAGAAAAACTTGAGGGCGACTTATCTGTGGGAGTATAAACAGTGGCCATTGCCTCTGAAATTGGCTCTGGGTTCTTTGATTGCCATTTTGGCAGGAGGAGCTATTTGGTATTCATTGCAATCCTTGTGGTGCATGTCTGGAGATGCTTCTTTTATAGCAGGAGCTGCCACGGTTTTTTCAGTATCTTCGTTCACGGGACAAAGTGATATACCCAATCGAGATAACTCGGAGAGGTCATTTAGAAACAGGAAGATACGTGCAAGAACCTGGCAAGGTCAAAGCTCATGCTTTGGAGACTCAGCATTATGGATTGCGGAGACGTGCGTGGCAACACTAACGTTTTCCAACGTGAGGACACAAGTATGCCTAGCCCCAGGTAGAGGGTTTTTTGGAGTGAATCATTGCTTAGCGGCAATTCCTGCAGGAGTTATGGTAAAGATGGACTCGAGCATAGGGGTCACATATTTCGTATGGGAGAAAGAAAAGTTGTTGCAATTTGAAGGCAATGAGGTGGCATTGTACATGACAAGCACATTGCCCAAAACTGTGGATTCTCTTCTGAGCAGAATTCACTTTGATGTGGAAACTTTGCCTAAGACCTTTAGCGCTGTTTTCTTTTCTTATAAGTATGATCCAATGACTCAGCAAATGGTGCCTGAACTTGGGAGTGTGACGTGTAAAGTTCATAACAAGGCTTACACATTGGCTCATGGGGAGTACAGGCGGGAGATCCCTCAAAGTCTCTCCTATGAAGCCAGTACTGTTGCTGGTGATTGTGGTTCTCTGATAATGGCTGAAATTGAAGGGAAATTCAAGCTAGTGGGTATGCATGTGGCATTTAATGGCAAAGAAGGGAGTGCAAGTTTTATGCCCTACCATGCTAGCTTGGATCAAAAGGTTGGTCAGGGAGACTTTATACTTAAATATCAGGAGTGGGCTGAGCCGAAAATTTTGGGACCGGGTTGTAGAGCAATGGGTCTTATAGAGCCTGAGCATGCTTTGGCAGCCAGTGGGAAAACAACATTTGTGGAAACCCCGGAGGAGTGGCACTTAGATTACCCATGTGATAAACTTCCAAGTGTGCTTGCCCGAGGGGACCCTAGATTGGCAGGGACGGTCCATGCAGATTATGACCCTTTTGCTTCCGGAATGAGCAAATATGCAAAAGAGGCGGGCCCCTTTGATGCTGCAAGCCTCAAGCAAGTGTGTTCAGGGATAGTTGAAATCTGGGAAGATGCTTCAGCAGAATTTCCCATGGATGAGGTTGATCTGGACACTGCTATTAATGGTTTGGAGAATGTCGAGTTCTTTGATGCTTTGGTTTTAGGGACATCTGAGGGATTTCCTTATAGGCTGGACCGGGGCCCTGGCGATAAAGGAAAGAGTAGATATGTGTCTGGTGAGAGTGGAAATTTGAAAATAACTGATGAAGGAGTTCTCTCGGATATAGATTGGTTTGAGGAAGTGAGCAAAACACAGGTACCAGATCTTTATTGCATTGAATGTGTGAAAGATGAGAGGTTGCCGATTAGGAAAGTGCTCCATGAGCCTAAGAGCAGGTTATTCACAGTCCTTCCAATGTCTTACAACATTGTCATCCGGAAGAAATTCTTAAATTTTGTTAGATTTTTCATGAAGAGGAGGGATGTTTTACCAGCTCAAGTTGGCATTAACCCTTACTCGCGTGAATGGACTCGAATGGCTAACAAGTTGTTGAGCAAGGGGAATAACATTTTGTGCTGCGATTATAGCAGATTTGATGGCTTTTTGCCCAAATGCATCATGAATGAAATAGGAGATATGATAGCCAGGGTAATGAAGGTGAATGAGGAGTCTAAGACGCAAATTAAGAACTTGATGCTTGCATGCACTAGTCGGTATGCAATGTGCAATCGAGTCTTGTACAGAGTTGAGAATGGCATTCCTTCTGGTTTTCCATTGACTGTCGTCGTGAACTCTATTTTGAATGAAATATTGGTCAAGTATGCTTACTGGCATTGTTTTGAGGATAATCCAAATGTGCAAAGCAACTTTGACGCGCATGTGTCAATGGTGGTTTACGGTGATGATAACTTGATTTCTGTGTCAGATGCTATAAGTTCAAAGTTTGATGGGAATTTCCTTGTGAATTTCATGGAGAGTTTAGGGATAAAAGTGACTGATGGCATCGATAAGACAAAGATAGGGATTGAATTCCGGAGATTGGAGAATTGTGATTTCTTGAAGCGCTCATTTAAGATGAATCCAGATGGAACATGGCGTTGCCCTATGTCCAAAGAAAGCTTGTGGCCACAGTTACATTATGTTAAGGCAAAGAAACTGGAAATGGCTGAAGCTTACATCAACAATTGCAACAACATTCTTCGTGAATTATGGCTGCATGATGTCAAGGAGGCAAAGGAATTTCGCAACAAGGTGTTGAGGAGCTTGAAATGGATTGGCCATGATCAATTGCTCAATATGCAACAGCTGGCAGTGTTCCACAGTGAACAAATGAATGGAGCTAGCGACTTTTTGTCTGCTTGTGTGACAGTTGATAGCATTTCCTTGATGGATCCTTTGGTACCAGGTATGTTGCCAGTTAAGACTTGTGAGATCATTCCTCGTGTTTTCGTGGCAGCAGAGAAGCATTTTGGAGGAGACTTTGAAGATTACTTCACAATTTCAATAACCACGAGTCGCAAATTTGAAGAGGATAAAGGCTTTGTTCTTTTGTTCCCGTATGGAGCTGGTAGAGGAGGTTTGCCAACAACGCAGTTCATGAAGGAAAATGTTGTAAGAAAAGGATGCTCGATACAGAAGAAGTTCAGGCAAGCCTATGAAAAAGGAAACAAGATTTTGTTCATATCGCAGAGTTCAGTAGTGCCTGCTTACGTCTTTGCTGTGATGCTTTTGCATTCCATTGGAGCAATTAACAGGTTGACAAGCAACAAGGCTCTAACTCAAGCTATGCAAACTTGCAAAAAGTTGGAATATCTACCTAGAGAGTATGAAGAGTTCTTCTGATTGCTTGTATGTTGTATGTATGTATGTTATGTAAATCACTACTTATATTGTATGGTAAGTACTATGCAGGGTTGAAATGCCTTTTCCCAAGTAAATTTCAGTAGTGAGGG

>RNA2-ZRHB [organism= Broad bean wilt virus 2] isolate ZR-HB segment RNA2, complete CDS

GTTTTAATAAAATATTAAAACAAACAGCTTTCGTTCCGAGAAAACAGCTTTCAGTCATTAATACAGCTTTCAGTTACAAAACAGCTTTCGGATACTCTGGAGTTTTCAAATTGAACCCGGAAAAAGGGAGTGTGATTCAAAGCGCACCATATACTTTGAAAATTTCTTTTCTTTTCTTGTTTTCATCAACGTATTCCACACCCAAAATTTTGTTTTTGGTATGTTGTAATGCGTCCCGAGCTTGTTGCAGTTTTAGATAGACATTTTTCGGAAATCATAAGTTGCTTCTTCTTGGGTTGGGTAATAAACTTTTTGCGTGTTTATTTTTGTTCTGCAAACAGTGCATTCTTATTGTGGGCAGCTTTCTTATACGTTTCTTACTATATATTGAGATTCGAATTTGCATATATCGTTGCGCCCTTCTTTAAAACGATATACGTAAATAGTTCTCAGTATCACGTTGTAGATTGGGAAAACGCGTACACAGCCGTTCCTAAAGGATTGTGGGAGCAAATCACTGATTATAATTATTGTTTCAATTTTCCCTCTCCGTCTGTAGAGGGATTTGTGTCTGACTTTTCACCGCGATTTACACTCAAAGAGCTTGAAATCATGAGCGAAGCAAACATTACCCCAGTGCATACAATTCCAAAAGACACCTTGCTTAAGAGAGCAAGTGATTACAAACTGGCTGTGGAGAGCAAGAAGTCCATTTTACCTAAAGTGCAGGACTTGTACGAGATGGACAAGTGGCACACTCTGAGGAGCAAACTGAGTAAGAATGCTCCTAATTATGTCACGACATCTGAAATAGTAGTTGGTGCAATGTCAGGTGCGGGAAACACAAAATTGGCAATACCAATTGTGGAAAAGTATACTGAGGAAGTGGCAGACGACAGGTTGCCTGATAAAATTCGTGCCAAGGCTGACCAGATAATGGTTGCGGCTATTGAATTAGTGGCAGATGGTTTTGCCTCGGTCAACTCAGACGTGACCATGGCTGGTGCGCTTTATGATAAACGCCACAAAACCATTGCGAGTTCTTTCAAGGGTGCTTTTGCATCCAGAGCTAGTGGAGTCCCTTCACACGTCATCTATTTTCCGATGCATAGGGTTCCTGCAAGCGATGATCCAAATACAACTTTGGAACTCTCAATGGTGAGCCGGGATTCAGATTTCGATGAAAGCTACACATTGGCCAACATTTCAGCTCGTACTCTTTATGTTCGTGCAAAGGGACCTGAAAAGGTTACTGAGACAAGGCATCTTTTGAAAGCCAAAACTGAGGATGTGGTCAAAGCGCGCCAATTTGCTAGTGAGGCACAAGTTGTATTTGCCACGCCCCGGTTATTTCCTGAAGTGAATTTGGATAACTATAATTTACCTGGGCCAAGTAATGTGCAGCAAACAGAGGCCATAACAACTGATAAAGGGATATTGTTTCCAAAGCCAAAGTTTAAAGGAAATGAAGTGGTGCTAAACTACACAGAACCAGCGAAGACTAGGAATCATGATTCACATAGATTTGTCAAGAAAGACACTTCTGATGAGCAATTAGTTAGGGGCGTTGAAGATCTTGGGTGTTTGTCTGATGAGGATGGCAAGGATTACAGATATGGTCAGGGTTTGATGGAAGAAGATGTTCTGAATGTGCAAACGAACAATTTTGCTATAGAATCAGCCACAGAAACTATGCGTTTGCTGTTCAGTGGACATGCAAGTATTCCTTTAAATGTGATACCTGGGACCAAAATTGCCGTGGCCTACCTGAATGAATTGTCCAAACATAGTGCTGTACATACTGGTTTGCTAAACATGTTGAGTAAAATTCCAGGTTCCTTGAAGGTCAAAATTAATTGTCAAGTGGCACCAACATGCGGTATAGGGTTGGCAGTTAGTTATGTGGAAGGCAATGAAAGCGCAAATTTGGGTTCAAGCCTGGGACGCTTGTTAGGTATTCAACATCACAAGTGGAATCCGGCCATAGAACCATTCGTGGAATTCATTTTCAAGCCTTTCTCCTGTGCAGATTGGTGGAACATGCACTACTTGGGATCCCTCAAATATGCTCCTGTGGTGGTCATACAAACATTGTCCAAGTGGCTAAATGCTCCGAAGGTTGATGCTAGAATTAGCTTCGCAGTTTACTATGAGCCCTCCATTGTGTTGCCCAAACAAATTGCAACTTTGGAGCATGCCCCAGCGTTTATGTTTCGCAAGGAGCTAGGAACTCTGGCTTTCAAACAAGGGGAGCGTGTGGCCTACTCTTTTGAAGTCAATTTTGGCAAACCACAGACCGATGGAAAGGAAGTGACTTCAACTTTTGCCTCTTCTTATTGTGGTCTCAGCCAATATATGCAATCGGATGTCATTTTGGATTTTACTCTTATGAGTAGTCCTATGATTGGTGGAACTTTCTCGATTGCTTATGTAGCTGGTGCTTACATTGAAAAGATTGGGAACATGCAAGTTCTTGACTCATTGCCCCACATTGACTTTACATTTTCTGCAGGGTCTAAGAGTACCCGCTCTGTGCGGTTTCCCAAAGAGGTTTTTGGGGTGTACCAGGCACTTGATAGGTGGGACTTGGATTCAGCTAGAGGAGATGATGTCTCAGGCAACTTCGTGCTTTACCAGAGGGATGCAGTTTCGAGTGCTTTGGAAGGAGAGCTTACCTTCAGAATAGCTGCTCGTTTGTCCGGAGATATCAATTTCACAGGAGTTAGTGCAGGCTATCCAACAACGATCACGCGTATAGGGAAAGGCAAAACTATAGGGAGATCACTTGAACCTGAAATTAGGAAACCTCTGAAATACATGCTTGGTCAAGCACATGCAACGCCAAAGGATTTCAGCTCAGTGCGTTTTGTGATGGGTCATTGGAAGTACAAGGCAGGTTTGTATCCCGGAAGTAAGTCAGATGAGGACATTCATCCATTCTCCTTGAAGATGCGCCTTGATGGATCCAAGAGTAGTGAGAATTTTGAGATTATACATTCCCCTTTCGTGCGCCTGTTGCAGAACTGTGCATGGATGAGAGGCACTTTGAAATTTTATGTCGTTGCCCGTGCTAGCTCTGACTACATGAGTTACAGGAGAACTTCTCAATTGACGGTTTCAGCTCACGAGAATAGTCTCAGTTCAAACCAATTCTACAGTGGAGTTTTGACGAGCCCAAGTGGCGAATTGAGCTTTTCCAGGGAAGTGGTTGGTCCAGTTGACGGCTTTGCCTCTATGGGTTGGAATGTTCGTGGGAGCAAGAAGTTTTACAAGGTGCATGTGGAAATGGGAAATGTTCATGAATACGATACCGTGGTGTTGTACGGACAATTTGGCTCTGATGTCGAGTTTGCTGGCCAACAAAAAGGAGGTCACTATTTGTTAGAGAAGGAAACTCCTATTTTCAAAACAATCAAGTATTAATAATTTTAGTAGTTATTTGGCGCTTGAATTTCAATAAAAAGGCGTAGGTCATTAGCCTCCCATTAAAATGGGTTCCATGACCTTTGTTTGTTTGATTTGTAATATTTACTACTTTTAAAACACATGTGTGCTGTGTTAGGTTAAAGCCTCCTATATATATATGCTTTAGTAGTATTTT

>RNA1-DZ [organism= Broad bean wilt virus 2] [isolate= DZ] segment RNA1, complete genome

GTTTTAATAAAATATTAGAACAAACAGCTTTCGTTCCGAAAACAGCTTTCAGTTACAAAACAGCTTTCGATCCGACAAAACAGCTTTCAGAAACAAACAGCTTTCAGTTTTACTTGAAGGTTTTCTTTTGCGTGACTTTGGAAAGAACCCGGAAAAAGGGAGTGTGATTTAAAGCGCACCATATATTTCAAAGATCATTTTGAATTTCATTTTGCTACCTTAATTTTAAGATGGATTTTAGTATGATGCAAGTGTTGGTGGGATTTTTGAAAACTTCGATGGGTTTACAATCCATCCGAGATATTGTGCGGAAGGCACAGGTGGAGGAGGAAAATAAAACTTTACTCCACATCCATTTGTGTTTTTTCCACGCTAATGAAATGGCGCGAGATTGGAATGAAGGTATGGACATGGACCAGATACATAGCTCTGCAGCTATAAAATATCGGTCCGCAATTGTGATGCGCCATGTTAAGATGAATGTGGAAACCGGAAGATACGATAGGAGGTGCATGTTAGAGTATAATAACTCGACCTGTGTCAACTGGTTTTCATGTGAATTTTCAGAAGAACAGCAGGAGGAAAACCCGGATGAGGGAGCCCAAGAGGAAGAAATTGTCAATGAATTCCTTGAAAGGTGTAATATTACAGAAGAAGAAGGTGCGCAAGCGCCTGCTGAAGCTGGCTCAAGCTATGCTTTTGGCCAAGGGTTGTACGAGTATGCTACTCGTATTGGCGACACAATAGTCGCAGCAATTTCAGGCTCTATTAAGAAGGGCATAGATGAATTTTTGGAAAAAGTTTATGCGGTCATGATGCAAGTGTTTGCTGCTTGGATGCCCAAGATTAGAGCTGCCTTCCAGTGGTTTGAGAATATCAAGGAAGTGATAAAGAAGTGGGCAAACACTATGCACGACAAGATAAATTACATTTTGGTCGGAATGGAGGATTGCCTATATATGGGAGCTGGACTTGTTGCAGCCACGTGTATAGTCACGTTGTTAGAAAAGTTCATGATAGCGATTGGTATACTCACAAAACCGTGTGGGGCAGCGACCATATTCTTGACAACTGCAATGGCAGCTATTTCGGCCACATATGTTTGTGCCAAAGCTGTGGAGAAGACAGTTATGCTTACGAACTTATTGCATTTCGTGTCCTCCAATTGTCAAATTGTATTGAACGCATTGTTCAATTATGAAATGACTAGGAAGGAACAATCCTCAGGCAATGGGGAAAATGAAACTCCAGTTGGCCTAGGCCAGTTTGGAGTGTCTTCAATGCTGCAGGACGTTGCCAATTTAATGTCGACATGGTCTATGGGAACAGTCACAGAAATTGGACGAACATTTGGAGCAATCTCTCAAATTAAAAACGGGATCGTGGCCTTAAAGGACATGGTTGTCTTTGTGTTTGAGAAATTGAGTGAATTAGCTCACAAAGTGTTAGGATTTGAGTCCCAGGTGCTGGCGGATCTTTCTGTTTTGCTCGGGGAAAATGTTGCAGATTGGTTGACTGAGTGCGATTGCATGGTAGCCTATATGTTGGAGTTTAACTCCAGGAATAGGGAGATCTTTGATCGCTTATCGCAACTGATTGAAAAGGGCAGATTGATAAGGGCAGGAGTGCTGAGGACGAGCCACCGTGGTTCATCGCAAGTTATGGCACTCGTTACTAAAGCTTTGGAAAAGCTGATTGAATTGCACAACTCGGTTGTGATGTCAGGATCAAATACAACTAGAAAATCCCCATTTATGATTTTCTTCACAGGAGCGTCGGGCACGGGGAAGACTTCAGTGGTTCAGAGGGTGGCCATCAATTGGTTGCAGGAGGAACAGCTTGGTACAAGTGAGATTTATGCACGCAACGGGCAGGATCCATTTTGGTCTGGCTACAAGAGGCATGCAGTTGTCACCTATGACGATTTCGGAGCTGTGCCAGGCACAACTTCAAATGAAGCAGAGATCATCAATGTAATTTCAAGGAATCCGTACGCCACAGTCATGGCGGGACTAGCAGAGAAAGGAATGTACTTCGATTCGAGGTTGGTTTTGGCTAGTAGCAATTTTCTAGCTGCCAATCCAGAGTCTGGAGTTCACGATTCCGAAGCATATGAAAGAAGAAGACATGCTGTTGTGAGGGTGTCTCTCAAACCTGGAGTGCCCTATAATGCTAATGATCCTTGTGCCAATCAGACATACACTTTGCTTGAGTCTAAAACACCCTTTAGGGAGATACGGACTTTTGAAACGTATGCGGAGCTGTGGTCTTTTTTGTACACAAGTTTCAAGGAGCATGAAGAGCAAGAAGAGCTGTATTTGAGATCTTTGCCTATTCTAGATTCAGACAAAAAAGAAGCACTGGAAGGTCTTATAGGTCTCACGGTGATAGCCACTTCGTTTGCTCCAAAGGCTGTCATGCAATTTGGAACCATCAAATTTCCAGGCTACCATTTTTTGGTTTCGGATGGAGAAAGATGTTACTTTTGGCATGGAGATGGCTCAGTGGAGACTGCCAGTGTGGAGCAGATGCAGCTTAACAAGCAGGATATTGCGCAGCTAAAACAACAGGGTCTATCAACAGCAATGATGTACAAGGATTTAGCCAAAGCTTTCCCAACTTTGAATTCATTGGCTGTATTGTATGCCAAGAACATTGTGGTGAAACGTTGGATAGGACCAGATTTGGGACCAACAAAGAATTGCGAGGATGTGTACATGCGTGAGCAGATTGGAAATTTGCCGAAATGGCAGAGAGCATATCTGTATGTGCTCAGCAAGTACTTGACAACCCAAAGTCCGCGAGGCTGGTTCATGGATTGTTTGGAAGAGACTAAGAAGAACTTGAAAGCGACATACTTGTGGGAATACAAGCAATGGCCATTGCCCTTGAAACTAGCCTTAGGCTCGCTTATCGCCATCATAGCAGGAGGAGCCATCTGGTACTCATTACAATCTTTGTGGTGTATGTCAGGGGATGCCTCATTTATAGCGGGGGCTGCTACGGTTTTTTCTGTATCTTCATTTGCAGGACAAAGTGACATACCCAACCGGGATAACTCAGAAAGGTCATTCAGAAACAAAAAGATACGTGCCAGGACTTGGCAAGGCCAAAGCTCATGCTTCGGGGACTCAGCTTTGTGGATTGCGGAGACGTGTGTGGCAACGCTAACATTCTCCAATGTTAGGACGCAAGTGTGCTTGGCTCCTGGCAGAGGTTTTTTTGGAGTGAACCACTGTCTGGCAGCAATTCCTGCAGGAATCATGGTCAAAATGGATTCAAGCATAGGGGTGACATATTTCATATGGGAAAAGGAAAAGTTGTTGCAATTTGAAGGCAATGAAGTGGCATTGTATATGACAAGTACATTGCCCAAAACTGTGGATTCTCTTTTAAATAGAATTCATTTTGATGTGGAAACCTTGCCTAAAACATTCAATGCAGTTTTCTTTTCTTACAAATATGACCCAATGACCCAGCAGATGGTGCCTGAGTTAGGGAGTATAACATGTAAAGTTCACAATAAGACCTACACATTGGCTCATGGAGAGTACAGGCGTGAAATTCCCCAGAGCCTTTCATACGAAGCTAGCACTGTAGCTGGTGATTGTGGCTCGTTGATAATGGCTGAGATAGAAGGAAAATTCAAGCTAATCGGTATGCATGTTGCTTTCAATGGCAAAGAGGGAAGTGCGAGCTTCATACCGTATCATGCCAGCTTAGATCAAAAGGTTGGCCAAGGGGATTTCGTGTTGAAATATCAAGAATGGGCCGAACCAAAAATTTTGGGTCCGGGTTGCAGAGCGATGGGTCTTATAGATCCTGAGCATGCTCTAGCTGCTAGTGGTAAGACAACATTTGTGGAAACTCCAGAAGAGTGGCACTTAAATTACCCGTGTGATAAACTTCCAAGTGTGCTTGCTCGAGGAGATTCTAGACTAGCAGGCACAATCCATGCAGATTACGACCCATTTGCTTCTGGCATGAGCAAGTATGCCAAGGAAGCAGGTCCATTTGACGCAGCAAGTCTCAAGCAGGTGTGTTCGGGAATAGCCGAAATTTGGGAAGATGCTTCAGCTGAATTTCCCATGGATGAAGTCGACTTGGATACTGCTATTAATGGACTTGAAAACGTTGAATTCTTTGACGCTTTAGTTCTGGGAACTTCTGAAGGATTCCCCTACAGGTTGGATCGAGGCCCAGGAGATAAAGGCAAGAGTAGGTATGTGACAGGCGAGAGTGGAAATTTGAAGATTACAGATGAGGGAGTACTTTCGGATATTGATTGGTTCGAGGAAGTGAGTAAAACGCAAGTGCCAGATCTTTATTGCATTGAGTGCGTTAAAGATGAAAGATTGCCGATTAGGAAAGTGCTGCATGAACCTAAAAGCAGATTGTTCACAGTCCTCCCAATGTCTTACAATATAGTCGTTCGGAAAAAATTTTTAAATTTTGTCAGGTTTTTCATGAAAAGGAGGGATGTGTTGCCAGCCCAAGTTGGCATAAATCCTTATTCACGAGAATGGACTCGAATGGCCAACAAATTGTTGAGCAAAGGGAACAACGTTTTGTGCTGTGATTACAGCAGATTCGATGGTTTTTTGCCTAAGTGTGTCATGAATGAGATAGGAGACATGATAGCCAGAGTTATGAAGGCAAATGAAGAATTAAGGACACAGATCAAGAATTTGATGCTTGCTTGCACTAGCCGTTATGCCATGTGCAATCGTGTCTTATATAGGGTCGAGAATGGCATTCCTTCTGGATTCCCTTTGACTGTCATTGTGAACTCCATTCTGAATGAGATTTTGGTCAAGTATGCTTATTGGCATTGCTTTGCGGACAATCCAAATGTGCAAAGCAATTTTGATGCGCACGTCTCTATGGTGGTTTATGGCGATGACAACTTAATTTCTGTGTCAGATGCCATAAGCTCAAAGTTTGATGGGAGATTTCTTGTGACTTTTATGGAAGGCTTGGGCATCAAAGTGACAGATGGCATTGACAAGACAAAGATTGGCATTGAATTTCGGAGATTGGAGGACTGTGATTTTCTGAAGCGTTCTTTCAAAATGAGTCCAGATGGCACGTGGTGCAGCCCCATGTCCAAAGAAAGCTTATGGCCACAGCTCCACTTTGTTAAGGCAAAGAAGCTTGAAATGGCGGAAGCTTACATCAACAACTGCAACAACATTCTTCGAGAATTGTGGTTACATGATGTTAAGGAAGCAAAGGAATTCCGCAACAAGGTGCTCAGAAGTTTAAAGTGGATTGGACATGACCAATTACTTAATATGCAGCAATTGGCTGTCTTCCACAGTGAACAAATGAATGGGGTTAATGATTTCTTGTCTGCGTGTATTACAGTTGATAGTATTTCTTTAATGGATCCCCTAGTCCCAGGTATGTTACCGGTTAAGACCAGTGAAATTGTCCCTCGGATTTTTGTGGCAGCGGAAAAGCACTTTGAAGGGAATTTCGAAGACTTTTTCACAATTTCAATTTCCCCAAGTCGTAAATTTGATGAAGATGGTGGGTTTGTTCTCTTGTTCCCTTATGGGGCTGGCAGAGGAGGCTTGCCAACGACACAATTTATGAAAGAGAACGTTATCAGGAAGGGATGCTCGATACAGAAGAAATTCAGGCAAGCGTATGAGAAAGGAAACAAGATTTTATTTATCTCACAAAGTTCAGTGGTTCCTGCTTACGTTTTTGCCGTGATGCTTTTGCATTCCATTGGGGCAATTTCAAGAATGACGAGCAACAAGGCTTTTACCCAAGCAATACAAACGTGTAAAAAATTGGAGTATTTTCCTAAAGAGTATGCAGAGTTTTTTTAATAACTATGTTTCGTGTTTGTGTATTTTATGTAAATCCCTACTTATATTGTATAGTGTGCCCTATGCAGGGTTGAAATGCCTTTTCCCAAGTAAATTTCAGTAGTGAGGG

>RNA2-DZ [organism= Broad bean wilt virus 2] [isolate= DZ] segment RNA2, complete genome

GTTTTAATAAAATATTAAAACAAACAGCTTTCGTTCCGAAAAACAGCTTTCAAATTTCAAACAGCTTTCAGACACTTTGGGATTTTCAGATTGAACCCGGAAAAAGGGAGTGTGATTTAAAGCGCACCATATCATTTGAAAATTTCGTTTTGATTTTTACTTTCTTTGCTAGTGTTATTTGTGCAAATATGAGATTTTGTTGTGATGAATCCTGAGTTAGTAGCTGTGTTAGATAGGTATCTATCTGAGATCGCAAGTAGTTTATTTTTAGGTTGGATTATAAATCTCCTTTTAGTTTTCTTTTGTTCCGCTAAGAGTTGTTTCTTGTTGTGGGCCGCATTTCTTTACATCAATTATTACATATTGAGATTTGAATTTGCATATATCGTTGCGCCCTTTCTTAAAACGATATATTCAAATAGTTCTCAATATCACACTGTTGATTGGGAAAACGCTTACACGGCACTTCCCAAAAATTTGTGGGAACAAATAACTGATTACAATTACTGTTTCAATTTCCCAAAACCCATTGGAGAGGGCTTTGTGTCGGTTTTTTCGCCTCGTTTTACGCTTGAAGAACTTATTGCAATGAATGAGGCAAATATCACTCCAGTTCACACAATTCCGAGAGAAACCTTGCTCAAAAGAGCAAGTGACTATAAATTGGCTGTGGAGAGCAAAAAGTCCATACTGCCCAAAGTTCAAGATTTATATGAGATGGACAAATGGCATGCTTTGAAGAGTAGGTTGAGCAAGAATGCGCCTAGTTATGTCGTGACTTCAGAGATTGCAGTTGGAGCTATGTCAGGCGCTGGGAATGTTAAATTGGCGCTGCCCGTGGTGGAAAAATACACTGAAGAGGTAGCAGATGACAGATTGCCTGACAAAGTTCGCGCCAAAGCTGATCAAATAATGGTCGCGGCCATTGAGTTGGTGGCAGATGGCTTCGCCTCAGTTAATTCTGATGTCACTATGGCAGGTGCGCTTTATGATAAGCGCCACAAGACAATTGCTAGTTCTTTCAAAGGAGCTTTTGCATCTAGAGCGAGTGGAGTCCCTTCTCATGTCATTTATTATCCAATGCATAGAGTTCCTTCAAATGATGATCCTAACACAACCTTGGAACTTTCAATGGTTAGTCGCGATTCTGATTTTGATGAGGGTTTCACGTTGGCTAATATCTCAGCACGTACTCTTTATGTTCGTGCAAAAGGGCCTGAAAAGGTGACTGAGACAAGGCATCTTTTGAAGGCCAAGACTGAAGATATGGTGAAAGCACAACAGTTTGCGAGTGAAGCACAAGTTGTGTTTGCCACTCCTCGGCTCTTTCCTGAAGTCAACCTGGACAACTACAATTTACCTGGGCCTAGCAATGTGCAGCAAACAGAGGCAATCACCACCAATAGAGGAATTCTTTTCCCAAAGCCAAAATTCAAAGGGAATGAGGTGGTGCTCAACTACACAGGGCCAACAAAAGTTAGAAATGTTAGTGTGCAGAGGTCTGGGCAGCAAGAGTTCAGCAGCAAACCATATGTGGAAAGCGTTGATGACCTTGGATGTTTATCAGATGAGGATGGCAAGGATTATAGATATGGCCAAGGCTTGATGGAGGAGGACGTTTTGAACGTTCAGACCAACAATTTTGCCATTGAGTCTGCCACAGAGACTATGCGCTTGCTGTTTAGTGGCTACGCGAGCATTCCTTTGAATGTTATACCTGGAACGAAGCTTACTGTGGCCTATCTTAATGAATTATCCAAGCATAGTGCTGTGCATACTGGTTTGTTAAATATGCTTAGCAAAGTCCCAGGTTCTTTGAAGGTCAAGATAAATTGCCAGGTTGCTCCTACATGTGGAATTGGATTGGCAATCAGTTATGTCGAAGGCAATGAAAGTGCAAACTTGGGATCTAGCCTGGGGCGCTTGTTGGGCATTCAGCATTACAAGTGGAATCCAGCTATAGAGCCTTATGTAGAATTTATTTTCAAGCCCTTTTCCTGCGCAGATTGGTGGAACATGCATTATTTAGGATCATTCAAATATGCACCTGTGATGGTCATCCAAACATTATCCAAATGGTTGAATGCGCCAAAAGTGGATGCCAAGATGAGCTTTGCCATCTATTATGAACCCAGTGTGATTTTGCCCAAACAAATAGCAACCTTGGATCACGCCCCAGCATTCATGTTTCGCAAGGAACTGGGGACTCTGGCTTTCAAGCAAGGAGAACGGGTGGCATATTCTTTTGAAGTCAATTTTGGTAAACCTCAGACAGACGGAAAAGAAGTGACATCGACTTTTGCCTCATCTTATTGTGGTTTGAGTCAGTACATGCAATCTGATGTGATCTTGGACTTTACTCTTATGAGCAGTCCTATGATTGGAGGCACTTTTTCAATTGCGTATGTCGCAGGTGCATATATTGAGAAAGTTGGGAACATGCAAATTCTTGATTCATTGCCCCATGTTGATTTCACATTTTCATCAGGTTCCAAGAGCACGCGTTCTGTGCGATTTCCGAAAGAAGTTTTTGGGGTGCATCAGGCATTGGATAGGTGGGATCTGGATTCAGCAAGGGGAGATGATGTTTCAGGTAACTTTGTGATTTACCAGAGAGATGCAGTCTCAAGTGCTCTTGAAGGGGAATTAACGTTCCGAATTGCTGCTCGCTTGTCTGGAGACATCAATTTTGTTGGTGTCAGTGCAGGCTATCCAACAACAATTACGCGAATTGGCAAAGGTAAGGCGCAAGGAAGATCGCTGGATCCTGAAATTAGAAAGCCTCTAAGGTACATGATTGGTCAGTCTCATTCAACACCACAGGATTTTAGTTCAGTGCGCTTTATGATGGGCCGCTGGAAATACAAAGCTGGTTTATATCCAGGGAGCAAATCAGACGAAGATATTCACCCGTACTCCCTCAAAATGCGCCTTGATGGCTCGAAGAGCAGTGAGAATTTTGAAATTATTCATTCCCCTTTTGTTCGGTTGTTGCAAAATTGTGCATGGATGAAAGGAACTTTGAAGTTCTATGTTGTGGCACGAGCTAGCTCTGACTATATGAGTTACAGAAGGACCTCTCAATTGACAGTTTCAGCTCATGAGAATAGTCTTAGCTCTAACCAATTCTACAGTGGAGTTTTGATAAGTCCTAGTGGCGAATTGGGTTTTTCCAGAGAGGTTGTAGGCCCAGTGGATGGCTTTGCATCTATGGGCTGGAACGTGCGTGGGAGTAAGAAGTTTTACAAAATTCATGTGGAAATGGGGAATGTTCATGAGTATGACACTGTGATGTTGTATGGGCAATTTGGCCCGAATGTGGAATTTGCTGGCCAACAGAAAGGCGGTCATTATTTGCTGGAGAAGGAAACTCCGACATTTAAGGCACTCAAATACTGATGAACAAGTTTGTTTTCTTTGTGTGTGTGCTCAAATAAAAGGGCGTGGGTCACTAGCCTCCCATTAAAATGGGTTCCATGACCTGTTAAATTTAGAAAATTACTATTTGTTAAACATATGTGTGTTATGTTGGGTAAAAGCTGCCTATGTATGAGCTTTAATAGTATTTC

>RNA1-XC1 [organism= Broad bean wilt virus 2] [isolate= XC1] segment RNA1, complete genome

GTTTTAATATTTATATTAAACAACCAGCTTTCGTTCGGAAAATAGCTTTCAGTTACAAAACAGCTTTCAGTGACAAACAAACAGCTTTCAGTTACTTGATTTGAGAAGGAACCCGGCAAAAGGGAGTGTGATTTCAAGCGCACCATAGTTTCTCAACATCAATCTACGATCTTTTCTTGGTTACTTATTTTTAAGATGGATTTCAGTTTGATGCAAACAGTTGTGGGTTTTTTGAAGACTTCGATGGGCTTACAGTCCATCAAAGACATAACACGGAAGGCACGCATAGATGAAAAAGATAAGACACTTCTTCAGATTCATCTTTGCTTTGTGCATGCAAATGAGATGGCTATTGAATATAACTCAGGTCTTGATGTGACCCAGATACATAGCCCTTCGGCTATAAAGTATCGTGCTGCGATACTTGCGCGTCATGTCCACCTGATGGTAGAAACAGGTAGGTATGACCGAAAGCGAATGCTTGAATACAACAACGAGACTTGTGTGAATTGGTTTTCTTGTGGTTTTGTTGTTGAAGAACCAGTTGAAACCCAAAGCAATGAGCAAGAGGAGGAGGACATTGTATCTGAGTTCCTCAGCCAGTGCAATATTGAAGAAGATGTGGCTAGCGGGGTCGGTTGCTCCAAGTCCAGAATAGCATTTGGGCAGGGCCTTTACGAGTATGCAACCCGCATTAGTGACACGATTGTGGCTGCTATCTCTGGTTCTGTAAAGAGAGGAATTGACGACTTCCTGGACCGGGTCTACAAGGTCATGGCCCAAGTTTTTGCAGCTTGGATGCCTAGAATCAAAGCAGCCTTCCAGTGGTTTGAAAACACAAAGGAGGTTATCAAGAAATGGGCAAATACAATGCATGAGAAGATAAATTGTATATTGGTTGGGATGGAAGACTGCCTGTACATGGGTGCAGGGCTAGTTGCAGCTACGTGCATAGTCACTTTGCTTGAAAAATTTATGATCACGATAGGAATTTTGACGAAACCTTGTGGAGCTGCCACATTATTTCTCACAGCTGCAATGGCAGCAATTTCAGCAACATATGTGTGTGCGAAAGCAGTGGAAAAATCAGTGATGCTTTCTAACTTGCTGCATTTTGTCACATCAAATTGCCAAATTGTACTAAATGCCTTGTTCAATCATGAAATGGCAACACGTGAGGCTGCAACATCGGAAGAGCAGGACAAGAGCATTGTTAGTTTTGGTCAATTTGGCATGTCAACTATGTTGCAGGATGTGGCTAACTTGATGTCCACATGGTCCACGGGGTCCATCACTGAGATAGGAAGAACTTTTGGGGCCATTTCGCAAATCAAGAACGGTATAGTAGCCTTGCGAGATATGGTCTACTTTGTTTTTGAGAAGTTGGGTGAGTTGGCCCATAAAGTCCTAGGATTCGAATCTCAAGTTTTGGCAGATTTATCTGTGCTCCTTGGAGAGAATGTGGCGGATTGGTTGTCTGAGTGTGATTGTATGGTTTCTTACATGCTGGAATTCAATTCAAGAAACAGAGAAATCTTTGATCGCTTGGCCCAATTGATTGAGAAAGGAAGATTGATTCGTACAGGGATACTCAGAACGAGTCATCGTGGCTCATCACAAGTTATGTCACTCGTGACAAAGGCTCTGGAGAAGCTCTTAGAATTGCATAACTCTATAGTTATGTCAGGTTCCAATACTACTCGCAAAGCGCCCTTTATGGTGTTTTTCACTGGTGCATCAGGAACGGGTAAAACCTCTGTGGTGCAGAGGATTGCCATCAACTGGCTGCAAGAGGAGCAACTCGGAACTAACGAAATCTATGCAAGGAATGGGCAAGACCCGTTTTGGTCTGGTTACAGGAGGCACGCTGTTGTGACATATGATGATTTTGGTGCTGTGCCAGGCACCGCCTCAAATGAGGCAGAAATAATAAATGTTGTTTCCCGGAATCCCTATGCAACTGTGATGGCCGGGTTGACAGAGAAAGGGATGTATTTTGACTCCCGGTTAATCTTGGCGAGCAGTAATTTCTTAGCTGCAAATCCAGAGTCTGGTGTTCATGATGCAGATGCCTATGAGAGGAGAAGGCACGTTGTCGTAAGGGTTGCTCTCAAGCCAGGGGTTCCTTATAACCCTAGCGACCCTTGCGCCAATCAGGTGTACACGATTGTTGAATCGAAGTCCCCTTTTCGGGAGTTGCAAGTGATTGAGACGTATGCCGAATTGTGGTCTTATTTGTACACTAACTTTAAGGCTCATGAGGAGAATGAAGAAGCTTTTCTCAAATCTTTGCCTATTTTGGATTCTGACAAAAAAGAGGCTCTAGAAGGTTTGATTGGGTTGACAGTCATTGCCACTTCTTTTGCACCCAAAGCTGTAATGCAGTATGGTGTGGACAGATTTCCGGGTTACCACTACTTGATATCGGATGGCGAGAGGTGCTATTTTTGGCACGGTGAGGGCCAGGTTGGCATTGTAGCTGTGGAGGCCATGAATTTGAGCAAACTGGATGTGGCTCAACTGAAGCAACAGGGCCTTTCTACAGCAATGATGTACAAGGATTTGGCAAAGACTTTCCCAACGTTGAATTCTTTGGCAGTCTTGTACGCAAAGAACATTGTGATCAAGAAGTGGATAGGGCCGGACCTTGAGCCAACGAAGAATTGTGAGGATGTTTACATGAGAGAGCAAATTGGTAGGTTACCACAATGGCAGAGGGCATATTTGCATGTACTCAGCAAGTACCTAGCAGTGCAGAGCCCAAGAGGCTGGTTTATGGAATGTCTTGAGGAAACGAAGAGAAACCTCAGAGCAACTTATGTGTGGGAGTACAAGCAATGGCCTTTACCCTTGAAGTTGGCACTGGGCTCTCTTGTCGCCATACTTGCAGGTGGGGCCATTTGGTACTCTCTTCAGTCTCTGTGGTGTATGTCAGGAGACGCTTCTTTTATTGCGGGGGCTGCAACAGTCTTTTCTGTTTCTTCTATGACGGGACAAAGTGATATCCCCAACAGGGATAATTCTGAGAAGTCATTTAGAAATAGAAAGATTAGAGCTAGAAGTTGGCAAGGCCAGAGCTCATGCTTTGGAGATTCTGCTTTATGGATTGCTGAGACATGTATGGCAACTTTGTCATTTTCCAATGTTAAATCTCAAGTTTGTCTTGCACCAGGCAGGGGTTTCTTTGGTGTGAATCATTGCCTCTCTGCTATTCCAAATGGGATTATGGTTAAGGTTGAGTCTAGCATTGGTAATACTTACTTTATGTGGGAGAGAGAGAAGCTCACGACTTTCGAGGGGAATGAAGTTGCTCTCTACACGACAAACACCTTGCCCAAAACGGTGGATTCATTGCTTGGACGAATTCATTTTGATGTGGAAACTCTGCCCAAAACTTTCCAAGCTGTGTTTTTCTCATACAAGTATGACCCCCTGTTGCAGCAGATGGTGCCAGAGCTTGGAAGTGTGACGTGCAAAATTCAAGACAAAGCTTATACTTTGGCTCATGGCGAGTATCGGAGGGAGATTCCACAAAGTTTGGTGTACGAAGCAAGCACTGTGGCTGGAGATTGTGGTTCTCTAATATTGGCTGAAATTGATGGGAAATTCAAGCTTGTTGGGATGCATGTGGCTCACAATGGCAAAGAAGGGAGCGCGAGTTTTATCCCTTATCATGCAAAATTGGATCAGAGAATCGGACAAAATGGCTTCATTCTCAAGTACCAGGAATGGGCTGAACCCAAAATTCTTGGACCAGGCTGTCGGGCAATAGGTCTCATAGATCCCGAGCACACATTGGCAGCAAATGGAAAGACGAGTTTTGCCGAAACGCCCAAAGAATGGCACTTAGATTATCCATGCGACAAAATTCCCAGTGTTCTAACTAGGGCAGATCCCAGACTTGCAGGTACTATTCATGCTGATTATGACCCATTTCCCTCAGGGATGAGCAAATATGCCAAAGAAGCAGGACCGTTTGAGGCAGATAGCCTGAAACAGGTCTGTTTGGGAATTTCGGAAATTTGGGAAGATGCGGCTTCAGAGTTTTCAATGGAAGAGGTTGACTTAGATACAGCCATCAATGGATTGGAAAATGTTGAATTTTTCGATGCTTTAGTGCTAGGGACCTCAGAAGGTTTTCCTTATAGGCTTGATCGGGGTCCAGGGGATAAAGGAAAGAGTAGGTATGTGTCTGGTGAAAGTGGGAGTTTGAAGATAACGGATGAAGGAATGTTGGCAGACATTGCTTGGATTGAAGAAACTTGTAAGAGCCAGGTGCCGGATCTCTACTGCATAGAGTGTGTGAAAGACGAGCGTCTGCCAATAAGGAAGGTGCTGCAAGAACCCAAGAGCAGGCTTTTCACTGTCCTTCCCATGTCCTACAACATTGTTGTTAGGAAGAAATTTTTAAATTTTGTCAGGTTTTTTATGAAGAGGAGAGATGTTCTGCCAGCACAGGTGGGTGTCAATCCCTATTCCAGAGAATGGACAAGGATTGCCAACAGGCTTCGTAGCAAAGGGAACAACATCCTTTGTTGTGACTACAGCCGGTTCGATGGGTTTCTACCCAAATGTGTGATGAAAGAGATCAGCGGGATGATAACCAGAATCATGAAAGTTTCTGGTGATTCAAAGGCCCAGATTGAAAACTTGATGCTGGCGTGTACCAGCAGGTACGCCATGTGCAATCGAATTCTGTATAGAGTTGAAAATGGGATACCATCTGGATTCCCTTTGACTGTAATTGTCAACTCAATACTGAATGAGATACTTGTCAAATATGCCTTCTGGCATTGTTTCTCTGATAATCCAAACGTGCAGTGCAATTTTGATTCGCATGTGGCTATGGTTGTGTATGGTGATGACAACTTGATCTCAGTCTCGGATGCCATTAGCTCTCAATTTAACGGGGACTTTTTGGTGAATTTCATGGAAGGTTTAGGCATCAAGGTCACTGATGGAGTTGATAAAACAAAAATTGGAATTGAGTTCCGGCGATTGGAGAATTGTGACTTTTTGAAGCGCTCTTTCAAGTTGAATCGGGATGGAACATGGCGAAGTCCAATGGCGCGTGAGAGTTTGTGGCCACAACTGCATTATGTTAAAGCAAAGAAAATTGAATTGGCAGAAGCCTATATCAACAATTGCAATAACATCTTGCGGGAATTGTGGTTGCATGATGTTGAGGAGGCTAAGACGTTCCGAGGCAAGATTCTTAAAAATCTCAGGTGGATTAGCGGAGATCAGTTATTGAATATGCAGCAGTTGGCAGTCTTCCATGACGAACAGATGAATGGGCATAGTGACTTTATGACTGTATGCACAACTAAGGACAACTTTTCCTTAATGGATCCAATTGTTCCTGGTATGCTACCAGTCAAAACTCATGAAATTGTTCCCCGAGTCTTCGTTGCGGCGGAGAAACATTTTACTGGTGATTTTAACGATTTTTTCACCATTTCTATAACGACAAGCCGCAAACTGGAGGAAGATAAAGGATTTGTCTTGGTTTTTCCATATGGCCCTGGCAGAGGTGGGTTGCCAACCATTCAGTTTATGAAAGAGAATGTCCTGCGGAAAGGTTGCGCCATTCAAAAGAAATTCAAGCAGGCTTATGAAGGAGGAAAGAATGTGTTGTTCATTTCTCAAAGTTCAGTCATTCCAGCATATGTGTTTTCAGTTATGCTTTTACATTCCATCAAAGCTATTAATAGAATAACCAGTAATAAAGCTTTGACACAAGCTATGCAAGCGTGTAAAAAGTTGGAGTATCTACCTAAAGAGTATAGCGATTACTTTTAAATATCGTTAATGTGATCTACTACTTGTTGTGTGTGTAGTGTGTACTATGCCGGGTTGAAATGCCCTTCCTCATAAGTATTTCAGTAGTAGAGG

>RNA2-XC1 [organism= Broad bean wilt virus 2] [isolate= XC1] segment RNA2, complete genome

GTTTTAATAAAATATTAAAACAAACAGCTTTCGTTCCGAAAAACAGCTTTCAAATTTCAAACAGCTTTCAGACACTTTGGGATTTTCAGATTGAACCCGGAAAAAGGGAGTGTGATTTAAAGCGCACCATATCATTTGAAAATTTCGTTTTGATTTTTACTTTCTTTGCTAGTGTTATTTATGCAAATATGAGGTTTTGTTGCGATGAATCCTGAGTTAGTAGCTGTGTTAGATAGGTATCTATCTGAGATCGCAAGTAGTTTATTTTTAGGTTGGATTATAAATCTCCTCTTAGTTTTCTTTTGTTCCGCTAAGAGTTGTTTCTTGTTGTGGGCCGCATTTCTTTACATCAATTATTACATATTGAGATTTGAATTTGCATATATCGTTGCGCCCTTCTTTAAAACGATATATTCAAATAGTTCTCAATATCACACTGTTGATTGGGTAAACGCTTACACGGCACTTCCCAAAAATTTGTGGGAACAAATAACTGATTACAATTACTGCTTCAATTTCCCAAAACCCACTGGAGAGGGCTTTGTGTCGGTTTTTTCGCCTCGTTTTACGCTTGAAGAACTTATTGCAATGAATGAGGCAAATATCACTCCAGTTCACACAATTCCGAGAGAAACCTTGCTCAGAAGAGCAAGTGACTATAAATTGGCTGTGGAGAGCAAAAAGTCCATACTGCCCAAAGTTCAAGATTTATATGAAATGGACAAATGGCATGCTTTGAAGAGTAGGTTGAACAAGAATGCGCCTAGTTATGTTGTGACTTCAGAGATTGCAGTTGGAGCTATGTCAGGCGCTGGGAATGTAAAATTGGCACTGCCCGTGGTGGAAAAATACACTGAAGAAGTAGCAGATGACAGATTGCCTGACAAGGTTCGCGCCAAAGCTGATCAAATAATGGTCGCGGCCATTGAGTTGGTGGCAGATGGCTTCGCCTCAGTTAATTCTGATGTTACTATGGCAGGTGCGCTCTATGATAAGCGCCACAAGACAATTGCTAGTTCTTTCAAAGGAGCTTTTGCATCCAGAGCGAGTGGAGTCCCTTCTCATGTCATTTACTATCCAATGCATAGAGTTCCTTCAAATGATGATCCTAATACAACCTTGGAACTTTCAATGGTTAGTCGCGATTCTGATTTTGATGAGGGTTTCACGTTGGCTAATGTCTCAGCACGTACTCTTTATGTTCGTGCAAAAGGGCCTGAAAAGGTGACTGAGACAAGGCATCTCTTGAAGGCCAAGACTGAAGATGTGGTGAAAGCACAACAGTTTGCGAGTGAAGCACAAGTTGTGTTTGCCACTCCTCGGCTCTTTCCTGAAGTCAACCTGGACAACTACAATTTACCTGGGCCTAGCAATGTGCAGCAAACAGAGGCAATTACCACCAATAGAGGAATTCTTTTCCCAAAGCCAAAATTCAAAGGGAATGAGGTGGTGCTCAACTACACAGGGCCAACAAAAGTTAGAAATGTTAGTGTGCAGAGGTCTGGGCAGCAAGAGTTCAGCAGCAAATCATATGTGGAAAGCGCTGATGACCTTGGATGTTTATCAGATGAGGATGGCAAGGATTATAGATATGGCCAAGGCTTGATGGAGGAAGATGTTTTGAACGTTCAGACCAACAATTTCGCCATTGAGTCTGCTACAGAGACTATGCGCTTGCTGTTTAGTGGCTACGCGAGCATTCCTCTGAACGTTATACCTGGAACGAAGCTTACTGTGGCCTACCTTAATGAATTATCCAAGCACAGTGCTGTGCATACTGGTTTGTTAAATATGCTTAGCAAAGTCCCAGGTTCTTTGAAGGTCAAGATAAATTGCCAGGTTGCTCCTACATGTGGAATTGGATTGGCAGTCAGTTATGTCGAAGGCAATGGAAGTGCAAACTTAGGATCTAGCCTGGGGCGCTTGTTGGGCATTCAGCATTACAAGTGGAATCCAGCTATAGAGCCTTATGTGGAATTTGTTTTCAAGCCCTTTTCCTGCGCAGATTGGTGGAACATGCATTATTTAGGATCATTCAAATATGCACCTGTGATGGTCATCCAAACATTATCCAAATGGTTGAATGCGCCAAAAGTGGATGCCAAGATGAGCTTTGCCATTTATTATGAACCCAGTGTGATTTTGCCCAAACAAATAGCAACCTTGGATCACGCCCCAGCATTCATGTTTCGCAAGGAACTGGGGACTCTAGCTTTCAAGCAAGGAGAACGGGTGGCATATTCTTTTGAAGTCAATTTTGGTAAACCCCAGACAGACGGAAAGGAAGTGACATCGACTTTTGCCTCATCTTATTGTGGTTTGAGTCAGTACATGCAATCTGATGTGATCTTGGATTTTACTCTTATGAGCAGTCCTATGATTGGAGGCACTTTTTCAATTGCATATGTTGCAGGTGCATATATTGAGAAAGCTGGGAACATGCAAATTCTTGATTCACTGCCCCATGTTGATTTCACATTTTCATCAGGTTCCAAGAGCACGCGTTCTGTGCGATTTCCGAAAGAAGTTTTTGGGGTGCATCAGGCATTGGATAGGTGGGATCTGGATTCAGCAAGGGGAGATGATGTTTCAGGTAATTTTGTGATTTACCAGAGAGATGCAGTCTCAAGTGCTCTTGAAGGGGAATTAACGTTCCGAATTGCTGCTCGCTTGTCTGGAGACATCAATTTTGTTGGTGTCAGTGCAGGCTATCCAACAACAATTACGCGAATTGGCAAAGGTAAGGCGCAAGGAAGATCGCTGGATCCTGAAATTAGAAAGCCTCTAAGGTACATGATTGGTCAGTCTCATTCAACACCACAGGATTTTAGTTCAGTGCGCTTTGTGATGGGCCGCTGGAAATACAAAGCTGGTTTATATCCAGGGAGCAAATCAGATGAAGATATTCACCCGTACTCCCTCAAAATGCGCCTTGATGGCTCGAAGAGCAGCGAGAATTTTGAAATTATTCATTCCCCTTTTGTTCGGTTGTTGCAAAATTGTGCATGGATGAAAGGAACTTTGAAGTTCTATGTTGTGGCACGAGCTAGCTCTGATTACATGAGTTACAGAAGGACCTCTCAATTGACAGTTTCAGCTCATGAGAATAGTCTTAGCTCTAACCAATTCTATAGTGGAGTTTTGATAAGTCCTAGTGGCGAATTGGGTTTTTCCAGAGAGGTTGTAGGCCCAGTGGATGGCTTTGCATCTATGGGCTGGAACGTGCGTGGGAGTAAGAAGTTTTACAAAATTCATGTGGAAATGGGGAATGTTCATGAGTATGACACTGTGATGTTGTATGGGCAATTTGGCCCGAATGTGGAGTTTGCTGGCCAACAGAAAGGTGGTCATTATTTGCTGGAGAAGGAAACTCCGACATTTAAGGCATTCAAATATTGATAAACAAGTTTGTTTTCTTTGTGTGTGTGCTCAAATAAAAGGGCGTAGGTCACTAGCCTCCCATTAAAATGGGTTCCATGACCTGTTAAATTTAGAAAATTACTATTTGTTAAACATATGTGTGTTATGTTGGGTAAAAGCTGCCTATGTATGAGCTTTAATAGTGTTTT

>RNA1-XC2 [organism= Broad bean wilt virus 2] isolate XC2 segment RNA1, complete genome

GTTTTAATAAAATATTGAAACAAACAGCTTTCGTTCCGAAAACAGCTTTCAGTTACTAAACAGCTTTCGTTCCGATTAAACAGATTTCAGAAACAAACAGCTTTCAACTTCATTCGAAGATTGACTTTTTGCGTGACTTTGGGAAGAACCCGGAAAAAGGGAGTGTGATTTAAAGCGCACCATATATTCCAAAGATCATTTTGAATTTCATTTTGCGACCTTAATTTTAAAATGGATTTCAGTACGATGCAGGTTGTTGTGGGTTTTTTGAGAACTTCGATGGGTTTACAATCCATCAAGGACATTGTCCAGAAAGCACAGGTGGCGGAGAAGGATAAAGTGTTGCTTCATATCCACCTGTGTTTTTTCCATGCCAATGAAATGGCACGGGATTTGAATGAAGGAATGAGCATGAGCCAGATACAAAGTTCTGCAGCAATAAAATATCGAGCTGCAATTGTAACGCGCCATGTCAAGATGAATGTGGAGACAGGGAAATACGACAGGAAGCGCATGTTGCAGTATAACAACGAAACGTGTGTCAACTGGTTTTCATGTGAATTTTCAGAGGAGACAGCCACGGACACATCAGGTGGAAGTGTTCAGGAAGAGGAAATCGTTAATGAATTTCTTAAGAGCCGTATCAGCATTGGGGAAGCTGGGCAAGCGTCTACAGGAAAAGGCCCGAGGTATGCTTTTGGCCAAGGGTTGTATGAGTACGCAACTCGAATTGGTGATGTGATAGTTGCGGCTATCTCAGGCTCAATTAAGAAAGGGATAGATGAATTCTTGGATAAAGTCTATGCGGTTATGACACAAATATTTGCTGCCTGGATGCCCAAGATCAGAGCCGCTTTTCAGTGGTTTGAGAACATCAAAGATGTTATAAAGAAGTGGGCAAACACTATGCATGATAAGATTAATTGTATTTTGGTCGGTATGGAGGATTGCTTGTACATGGGAGCTGGGCTTGTTGCAGCTACGTGTATTGTGACGCTGCTTGAGAAATTCATGGTAGCCATTGGAATTTTAACAAAACCATGTGGAGCGGCAACTCTTTTCCTAACAACTGCAATGGCGGCCATTTCAGCTACATATGTTTGCGCTAAGGCTGTGGAAAAATCAGTTATGTTAACTAGCCTGTTGCAATTTGTGACTTCCAATTGTCAAATCGTGTTGAACGCTTTGTTTAATTATGAGATGACGAGGAAAGAGCAATCTTCAGGCCATGATAGGGATGAAGCTCCGGCAAGCCTTGGCCAATTTGGAGTTTCCTCAATGTTGCAGGATGTCGCCAATTTGATGTCGACGTGGTCTATGGGAACTGTGACAGAAATTGGAAGAACATTCGGAGCCATCTCGCAGATCAAGAATGGAATTGTGGCTCTAAAGGATATGGTGCACTTTGTTTTTGAGAAATTGAGTGAATTAGCTCATAAAGTGCTAGGATTTGAGTCCCAGGTGTTAGCGGACCTTTCAATTTTGCTCGGTGAGAATGTTGCAGACTGGTTGTCTGAGTGTGATTGCATGGTGGCCTACTTGCTTGAATTCAACTCTAGGAATAGAGAAATTTTTGATAGGCTTTCACAATTGATTGAGAAAGGTAGGTTGATAAGGGCTGGTGTGTTGCGCACGAGTCACCGTGGCTCATCGCAAGTTATGGCACTTGTTACCAAAGCTCTGGAAAAGCTGATTGAATTGCACAATTCTGTTGTGATGTCAGGATCAAACACCACTAGGAAGTCCCCGTTTATGATTTTTTTCACAGGGGCTTCTGGTACAGGGAAAACATCGGTTGTTCAGAGAGTCGCCATAAATTGGTTGCAAGAAGAGCAGCTTGGCACAAGTGAAATTTATGCGCGCAATGGACAAGACCCATTTTGGTCAGGTTATAAGAGGCATGCAGTCGTCACATATGATGATTTTGGTGCTGTACCAGGTACAACCTCAAATGAAGCTGAGATCATAAATGTGATCTCAAGAAATCCATATGCGACAATGATGGCAGGGTTGGCTGAGAAAGGAATGTACTTTGATTCAAGATTGGTTTTGGCTAGTAGTAATTTCTTGGCTGCCAACCCCGAGTCGGGAGTCCATGACTCTGAGGCATATGAGAGGCGGAGGCATGCTGTTGTTAGAGTGTCCCTTAAACCTGGAGTGCCGTACAATGCAAATGACCCGTGTGCCAATCAGACATACACCCTTCTTGAGTCCAAGACACCTTTTAGAGAAATTCAAACTTTTGAGACATATGCAGAGTTGTGGTCTTACTTGTATACAAGTTTTAAGGAGCATGAGGAGCAAGAGGAGTTGTATTTGAAATCTTTACCTATTCTGGATTCTGATAAGAAGGAGGCTTTGGAAGGCCTTGTGGGCCTTACAGTGATAGCCACCTCTTTTGCTCCGAAGACTGTGATGCAGTTTGGTGCAAGCAAATTCCCGGGTTATCATTTTTTGATTTCAGATGGGGAGAGATGCTACTTTTGGCATGGAGATGGTTCAGTTGAGATAGTCAGCGTGGATCATATGCATCTGAGCAAGCAGGACATCGCACAATTGAAGCAGCAGGGTTTGTCAACTGCAATGATGTACAAGGATTTGGCCAAAGCTTTTCCAACTCTGAATTCACTAGCTGTGTTGTATGCCAAAAATATTGTGGTGAAACGCTGGGTGGGACCAGATCTAGAACCAACTAAGAGTTGTGAAGATGTGTACATGCGGGAGCAGATCGGAAATTTGCCAAAATGGCAGAGAGCATATTTGCATGTGCTCAGCAAGTATTTAACAGCTCAAAGTCCACGAGGTTGGTTCATGGAATGTTTGGAGGAGACAAAGAAAAATTTGAGGGCAACTTATCTGTGGGAGTATAAACAGTGGCCATTGCCTTTGAAATTGGCTCTGGGTTCTTTGATTGCCATTTTGGCAGGAGGAGCTATTTGGTATTCATTGCAATCCTTGTGGTGCATGTCTGGAGATGCTTCTTTTATAGCAGGAGCTGCCACGGTTTTTTCAGTATCTTCGTTCACGGGACAAAGTGATATACCCAATCGAGATAACTCGGAGAGGTCATTTAGAAACAGGAAGATACGTGCAAGAACCTGGCAGGGTCAAAGCTCATGCTTTGGAGACTCAGCATTATGGATTGCGGAGACGTGCGTGGCAACACTAACGTTTTCCAACGTGAGGACACAAGTATGCCTAGCCCCAGGTAGAGGTTTTTTTGGAGTGAATCATTGCTTAGCGGCAATTCCTGCGGGAGTCATGGTAAAGATGGACTCGAGCATAGGGGTCACATATTTCATATGGGAGAAAGAAAAGTTGTTGCAATTTGAAGGCAATGAGGTGGCATTGTACATGACAAGCACATTGCCCAAAACTGTGGATTCTCTTCTGAGCAGAATTCATTTTGATGTGGAAACCTTGCCTAAGACCTTTAGTGCTGTTTTCTTTTCTTATAAGTATGATCCAATGACTCAACAAATGGTGCCTGAACTTGGGAGTGTGACGTGTAAAGTTCATAACAAGACTTACACATTGGCTCATGGGGAGTACAGGCGGGAGATCCCTCAAAGTCTCTCTTATGAAGCCAGTACTGTTGCTGGTGATTGTGGTTCTCTGATAATGGCTGAAATCGAAGGGAAATTCAAGCTAGTGGGTATGCATGTGGCATTCAATGGTAAAGAAGGGAGTGCAAGTTTTATGCCCTACCATGCTAGTTTGGATCAAAAGGTTGGTCAGGGAGACTTTATGCTTAAATATCAGGAGTGGGCTGAGCCGAAAATTTTGGGACCGGGTTGTAGAGCAATGGGTCTTATAGAGCCTGAGCATGCTTTGGCGGCCAGTGGGAAAACAACATTTGTGGAAACCCCGGAGGAGTGGCATTTAGATTACCCATGTGATAAACTTCCAAGTGTGCTTGCCCGAGGGGACCCTAGATTGGCAGGGACGGTCCATGCAGATTATGACCCTTTTGCTTCTGGTATGAGCAAATACGCAAAAGAGGCGGGCCCCTTTGACGCTGCAAGCCTCAAGCAAGTGTGTTCAGGGATAGTTGAAATCTGGGAAGATGCTTCAGCAGAATTTTCCATGGATGAGGTTGATCTGGACACTGCCATTAATGGTTTGGAGAATGTCGAGTTCTTTGATGCTTTGGTTTTAGGGACGTCAGAGGGATTTCCTTATAGGTTGGATCGGGGCCCTGGCGATAAAGGAAAAAGTAGATATGTGTCTGGTGAGAGTGGAAATTTGAAGATAACTGATGAAGGAGTTCTCTCGGATATAGATTGGTTTGAGGAAGTGAGCAAAACACAGGTACCAGATCTTTATTGCATTGAATGTGTGAAAGATGAGAGGTTGCCGATTAGGAAAGTGCTCCATGAGCCTAAGAGCAGGTTATTCACAGTCCTTCCAATGTCTTACAACATTGTTATCCGGAAGAAATTCTTAAATTTTGTCAGATTTTTCATGAAGAGGAGGGATGTTTTACCAGCTCAAGTTGGCATCAACCCTTACTCGCGTGAATGGACTCGAATGGCTAACAAGTTGTTGAGCAAAGGGAATAACATTTTGTGCTGCGATTATAGCAGATTTGATGGCTTTTTGCCCAAATGTATCATGAATGAAATAGGAGACATGATAGCCAGGGTAATGAAGGTGGATGAGGAGTCTAAGACGCAAATTAAGAACTTGATGCTTGCATGCACTAGTCGGTATGCAATGTGCAATCGAGTTTTGTACAGAGTTGAGAATGGCATTCCCTCTGGCTTTCCATTGACTGTCGTCGTGAACTCTATTTTGAATGAAATATTAGTTAAGTATGCTTACTGGCATTGTTTTGAGGATAATCCAAATGTGCAAAGCAACTTTGGTGCGCATGTGTCAATGGTGGTTTACGGTGATGATAATTTGATTTCTGTATCAGATGCCATAAGTTCAAAATTTGATGGGAATTTCCTTGTGAATTTCATGGAGAGTTTAGGGATAAAAGTGACTGATGGCATCGATAAGACAAAGGTAGGGATTGAATTCCGGAGATTGGAGAATTGTGATTTCTTGAAGCGCTCATTTAAGATGAATCCAGATGGAACATGGCGTTGCCCTATGTCCAAAGAAAGCTTGTGGCCACAGTTACATTATGTTAAGGCAAAGAAACTGGAAATGGCTGAAGCTTACATCAACAATTGCAACAACATTCTTCGTGAATTATGGCTGCATGATGTCAAGGAGGCAAAGGAATTTCGCAACAAGGTGTTGAGGAGCTTGAAATGGATTGGCCATGATCAATTGCTCAATATGCAACAGCTGGCAGTGTTCCACAGTGAACAAATGAATGGAGCTAGCGACTTTTTGTCTGCTTGTGTGACAGTTGATAGCATCTCCTTGATGGATCCTTTGGTACCAGGTATGTTGCCAGTTAAGACTTGTGAGATCATTCCCCGTGTTTTCGTGGCAGCAGAGAAGCATTTTGGAGGAAACTTTGAAGACTACTTCACAATTTCAATAACCACGAGTCGCAAATTTGAAGAGGATAAAGGTTTTGTTCTTCTGTTCCCATATGGAGCTGGCAGAGGAGGTTTGCCAACAACGCAGTTCATGAGGGAAAATGTTGTAAGAAAAGGATGCTCGATACAGAAGAAGTTCAGGCAAGCCTATGAAAAAGGAAACAAGATTTTGTTCATATCGCAGAGTTCAGTAGTGCCTGCTTACGTCTTCGCTGTGATGCTTTTGCATTCTATTGGAGCAATTAACAGGTTGACAAGCAACAAGGCTCTAACTCAAGCTATGCAAACTTGCAAAAAGTTGGAATATCTACCTAGAGAGTATGAAGAGTTTTTCTGATTGCTTGTATGTTGTATGTATGTATGTTATGTAAATCACTACTTATATTGTATGGTAAGTACTATGCAGGGTTGAAATGCCTTTCCCCAAGTAAATTTCAGTAGTGAGGG

>RNA2-XC2 [organism= Broad bean wilt virus 2] [isolate= XC2] segment RNA2, complete genome

GTTTTAAAAAATACTTAAAACAAACAGCTTTCGTTCCAATAAACAGCTTTCACCAAAGTTTTCAAACAGCTTTCAGCTTACAACGTTTGTTCGTGGAATTGAAAAGAACCCGGTAAAAGGGAGTGTGATTTAAAGCGCACCATAGAGTTCAATTTCATGTTTTGTGTGGCTCATTTTATGTGTATTCTCACATGATTTCAGAACGAGTTGCCTTCTGGGACTCAGTTATTAGTGAAATAATTTTGTGTTTCTCTCTGGGTTTTCTTGTAGCTCTCACTTCGAGCAATTCACTTGGAGCTAAGTTTTACGTTTGGGGTGTCTTTTTCTGGTTTTGCTGGCAAATTTTAAAGCTTGAGTTCGAATATATTGTTAAGCCCTTTCTTTCAACAATATATCAGAACAGTAGCCAATATTACGAGCCAGGGACCTTTTGTGGGTACACTGCCAGTCCAAAAGGGTTGTGGGAGCAAATTTTCGATTACAACTACTGCTTTCGGTTTCCAAGGCGTTATATTGAGGGATACCCTTCAGCATTTTCTCCAAGATTCACGCTTGCTGAGTTAATGGCTTTGAACGAGGCAAATATCACGCCTGTGCATACTATTCCCAAGGATACTCTTCTGAAGCGCGCAAGTGACTATAAACTAGCAGTGGAGAGTAAGAAATCCATTCTGCCCAAAGTGCAAGACCTTTACGAAACAGACAAATGGCACTCACTGAAGAGCAAATTTGGAAAGAGTGCCCCAAGTTACATTGTCACATCTGAAGTGGCAGTTGGTTCTATGTCTGGGGCAGGTAATACCAAGCTTTCTCTACCAGTTGTGGAGAAATATACAGAAGAAGTGGCGGATGATAGGTTGCCCGAGAGGGTGCGTGCAAAAGCGGATCAAATTATGGTGACAGCCATAGAGTTGGTGGCTGATGGCTTTGCATCAGTGAATTCTGATGTCACTATGGCAGGAGCTTTGTATGACAAGCGCCATAAAACTATTGAGAGTTCTTTCAAAGGTGCTTTTGCATCACGTGCTAGTGGAGTTCCTTCACACGTGGTGTACTTTCCGATGCATAGAGTGCCTTCAAGTGACGACCCCAACACCACACTTGAATTGTCTGTCGTTAGCAGAGACACTGACTTTGATGAGTGTTACACTCTTGCTAACATTTCTGCTAGGACCTTATACGTAAAAGCAAAAGGGCCTGAAAAGGTTACTGAAACACGCCATTTGCTCAAGGCTAAAACTGAGGATGTTGTCAAGGCGCGGCAATTTGCTAGTGAGGCACAGGTTGTTTTTGCGACACCTAGGCTTTTCCCTGAAGTAAACCTTGACAATTATAAGCTTCCAGGGCCAAGCAATGTGCAACGCACCGAAGCGATTAGCACGAGCAAAGGAATCTTCTTTCTGAAACCCCAATTCAAGGGAGACCAGATTGTATTGAATTACACTGGTCCAGAAAGCATTCAAACCATCAAGGGTCAGGGTGCCAGCAAGAGCAGAAAGGATCAAGGCACTACTCACAAGCGAGAGAAATTTGTTGGAAGGATCGAAGATTTGGGTTGCCTGTCGGATGAAGATGGACGTGATTACAGATTTGGCCAAGCATTGATGGAAGAAGATGTATTGAATGTTCAAACCAACAATTTCGCTATAAGCTCGGCGACAGAAACCATGCGCTTACTTTATAGCGGGCATACAACCATTCCTTTGAATGTTGTCCCTGGGACAAAGCTCACTGTGGCCTATCTCAATGAGCTTTCCAAACACAGTGCGGTCCATACAGGCCTTTTAAATATGCTGAGCAAAGTGCCTGGCTCAATCAAAGTCAAAATCAATTGTCAAGTCGCACCGACATGCGGAATAGGCTTGGTAGTGAGTTATGTTGAGGGTAACGAGAGTGCAAACTTAGGCTCAAATTTAGGAAGACTACTTGGAATTCAACATTACAAGTGGAATCCAGCGATTGAGCCCTACGTGGAGTTTGTCTTCAAGCCTTTCTCTTGTGTTGATTGGTGGAATATGCACTTCCTAGGCTCGGCTAAATTCTCTCCAGTTATGGTTGTGCAAGTTCTCTCCAAATGGTTGAATGCTCCCAAGGTAGATGCAAGAATGAGTTTTGCACTGTACTATGAGCCGCAGGTGATACTGCCCAAACAAATTGCCACAATTCAAGGAGCTCCCGCTTTCATGTTCCGAAAGGAGCTTGGGACGCTGGCTTTCAAGCAAGGGCAACGGGTGGCATATGCCTTCGAGGTCAACTTTGGCAAGCCCCAAACTGACGGCAAGGAAGTGACACTCACTTTTGCCTCATCCTATTGTGGAATGAGCCAGTATATGCAAGCGGATGTCTTGCTTGACTTCACACTTCTTAGCAGCCCAATGATAGGTGGAACTTTTTCTGTGGCATACGTGGCTGGGGCCTTTATTGAGAAAATTGATAATATGCAGGTTTTGGATTCCTTGCCTCACATCGACTTTACCTTTTCCGCAGGAGGGAAAAGCACTAGGTCCATACGATTCCCCCGGGAAGTATTTGGAGTCTACCAGGCTCTTGATAGATGGGATTTAGATGCAACTCGTTGGGACGACGTTTCTGGAAGTTTTGTGCTCTACCAGAGAGATACAGTATCAAGTGCTCTAGAAGGAGATTTGGTCTTTAGAGTTGCTGCTAGGCTCTCAGGGGTTGTTGAGTTTCATGGGGTGAGTGCTGGATACCCCACTACAGTGACTAGGATTGGCAAAGGGAAGAGCCAAGGCAGGTCCCTGGAACCAGAGGTGCGCAAACCTTTGCGCTACATGCTGGGGCAGGATCACACCACACCTGGAGACTTCAGCTCTGTGCGCTTTATGATGGGGAGATGGAAGTATGAAGCTGGTGTGTTCCCAGGAAGCAAAGCAGATGAAGATATTCATCCTTTCTCCCTTAAAATGCGGCTTGATGGATCTAAGAGTAGCAAACACTTTGAGATTATACATTCCCCGTTTGTCCGGCTATTGCAGAATTGTGCGTGGTTGCGCGGGACTCTAAAATTCTTTGTCGTGGCTCGGGCCAGTTCTGATTATATGAGTTATCGACGCACTTCACAGCTTCTTGTTACTGCTCATGAAAATAGCTTGAGTTCCAACCAATTTTACAGCGGAGTCTTAACAAGTCCCAGTGGAGAATTAAGTTTCTCAAGAGAAGTCGTGGGGCCTGTCGATGGATTTGCTTCAATGGGTTGGAATGTGCGTGGAAGCAAGAAATTCTACAAACTTAATGTGGAACTGGGAAATGTCCATGAGTATGAATCAGTCGTTCTTTATGGTCAATTCGGTCCAGATGTAGAGTTTGCTGGGCAACAAAAAGGGGGTCACTATTCCTTAGAGAAGGAAGTTCCTGTTTTCAAAGCTATAAAATATTGAAACTTTTCTGTTGTGATTAATAAAAGAAGGCGTTGGCCACAAGCCTCCCATTAAAATGGGTTCCATGGTCTTTACTTGTATGTATATATTTATCTACTACTTTAAGCATATTGTGTTGTATGCTGGGTTGAAATTCCCTCCTCGTGTAAATTTCAGTAGTAGAGG
